# Supplementary material for: The Role of Cytochrome P450 AbyV in the Final Stages of Abyssomicin C Biosynthesis
Source: Angew Chem Int Ed Engl. 2022 Dec 8;62(3):e202213053. doi: 10.1002/anie.202213053 (PMC10107801; doi:10.1002/anie.202213053)
Supplement: Supplementary file 1 — Supporting Information [file ANIE-62-0-s001.pdf]

Angewandte Chemie
Eine Zeitschrift der Gesellschaft Deutscher Chemiker

Supporting Information

The Role of Cytochrome P450 AbyV in the Final Stages of Abyssomicin C Biosynthesis

A. J. Devine, A. E. Parnell, C. R. Back, N. R. Lees, S. T. Johns, A. Z. Zulkepli, R. Barringer, K. Zorn, J. E. M. Stach, M. P. Crump, M. A. Hayes, M. W. van der Kamp, P. R. Race, C. L. Willis**

Contents

1. Supplementary Methods 4

1.1 Gene Cloning 4

1.2 Protein Expression and Purification 4

1.3 Protein Crystallization and Diffraction Data Collection 5

1.4 Structure Solution and Refinement 5

1.5 Spectrophotometric Analysis of Compound 3 Binding to AbyV 6

1.6 AbyV Epoxidation Activity Assays 6

1.7 Analysis of AbyV Epoxidation Activity Assays 7

1.7.1 LC-MS 7

1.7.2 ^{1}H NMR 7

1.7.3 ^{13}C NMR 7

1.7.4 *In situ* Monitoring 7

1.8 Docking 8

1.9 AbyV Chain A Loop Modelling 8

1.10 Molecular Dynamics Setup, Simulations and Analysis 9

1.11 Compound Synthesis 10

1.11.1 General Considerations 10

1.11.2 Compound Characterisation 10

1.11.3 Synthesis of AbyV Substrate 3 and Epoxide 4 11

2. Supplementary Tables 28

Table S1. Summary of X-ray data collection and refinement statistics 28

3. Supplementary Figures 29

Figure S1. Proposed Abyssomicin C Biosynthetic Pathway. 29

Figure S2. Size exclusion chromatography and SDS PAGE analysis of purified recombinant AbyV. 30

Figure S3. Spectroscopic characterisation of AbyV. 31

Figure S4. Synthesis of Proposed AbyV Substrate 3 and Epoxide 4. 32

Figure S5. Spectrophotometric Analysis of Compound 3 Binding to AbyV. 33

Figure S6. ^{1}H NMR Analysis of Assay Products 34

Figure S7. ^{13}C NMR (DEPT) spectrum of AbyV assay mix 35

Figure S8. Sequence Alignment of AbyV with its Closest Structural Homologues. 36

Figure S9. Conserved amino acids identified in Figure S7 mapped onto the crystal structure of AbyV. 37

4. Supplementary References 38

6. ^{1}H and ^{13}C NMR Spectra 41

1. Supplementary Methods

1.1 Gene Cloning

The gene encoding AbyV was amplified from *Micromonospora maris* AB-18-032 genomic DNA by polymerase chain reaction (PCR) using the primers 5'-AAGTTCTGTTTCAGGGCCCGATGATCCCGTCTGCGGCG-3' (forward) and 5'-ATGGTCTAGAAAGCTTTATCACCAGGTGACCGGAAGGCTTGGAC-3' (reverse), introducing appropriate overlap sequences (underlined) for insertion into the plasmid pOPINF, pre-cut with *Kpn*I and *Hind*III. Ligations used the In-Fusion cloning kit (Clonetech) following the manufacturers protocol. The resulting construct, pOPINF::*abyV*, encodes an N-terminally hexahisitidne tagged variant of AbyV. The sequence of this plasmid was verified by DNA sequencing and transformed into *E. coli* BL21 (DE3) cells for protein expression.

1.2 Protein Expression and Purification

LB medium (100 mL) supplemented with carbenicillin (100 µg/mL) was inoculated with a single colony of *E. coli* BL21 (DE3) pre-transformed with *abyV*::pOPINF and incubated overnight with shaking (37 °C, 180 rpm). This pre-culture was used as a 1% inoculum for 1 L of TB autoinduction medium supplemented with carbenicillin (100 µg/mL). The cell culture was incubated (37 °C, 220 rpm) until an OD_{600nm} of 1.8 was reached and the culture was then supplemented with 5-aminolevulinic acid (final concentration 0.5 mM) and incubated overnight with shaking (28 °C, 180 rpm). Cells were then pelleted by centrifugation (4000 rpm, 30 mins) and stored at -80 °C.

Frozen cell pellets were thawed on ice and resuspended in His-Load buffer (50 mM Tris-HCl, 500 mM NaCl, 20 mM imidazole, pH 7.5). The cell suspension was lysed using a Cell Disrupter (Constant Systems Ltd, 40 Kpsi head) at 25 Kpsi. Following lysis PMSF protease inhibitor was added (final concentration 1 mM). The resulting cell lysate was clarified by centrifugation, and the supernatants containing protein were applied to a 5 mL HP HisTrap column (GE Healthcare) pre-equilibrated with His-load buffer. The column was washed with 10 column volumes of His-Load buffer and bound protein was then eluted using an imidazole gradient of

20 to 500 mM over 60 mL with fractionation (1 mL) throughout. Fractions containing the target protein, as determined by monitoring the absorbance of the column eluent at 280 nm followed by SDS-PAGE analysis, were pooled and concentrated to 5 mL. Concentrated protein samples were centrifuged to remove aggregates, prior to application to a 16/60 Superdex 200 column (GE life sciences), pre-equilibrated with 300 mL of buffer (50 mM Tris-HCl, 500 mM NaCl, pH 7.5). Fractions containing the target protein, as determined by monitoring the absorbance of the column eluent at 280 nm followed by SDS-PAGE analysis, were pooled, concentrated to 10 mg mL^{-1}, and flash frozen in liquid nitrogen for storage at -80 °C before use.

1.3 Protein Crystallization and Diffraction Data Collection

Conditions supporting the growth of crystals of AbyV were initially identified using the sitting drop vapor diffusion method at 18 °C, employing commercially available crystallization screens (Molecular Dimensions Limited), mixing 0.5 µL of protein solution with 0.5 µL of reservoir solution. Diffraction quality crystals were grown using the hanging drop vapor diffusion method, employing reservoir solutions containing 0.1 M Bis-Tris pH 7.0, 25% PEG 3350, 0.2 M $MgCl_2$. Crystals took 14 days to grow to optimum size. Crystals selected for data collection were mounted in appropriately sized litholoops (Hampton Research), submerged in reservoir solution supplemented with 20% glycerol, and flash-cooled in liquid nitrogen prior to analysis. Diffraction data were collected at Diamond Light Source, UK on beamline I04-1 using a Dectris Pilatus 6 M pixel array detector. Collected data was integrated using the xia2 3dii pipeline.[1] 5% of the data were set aside for the calculation of R_{free}.

1.4 Structure Solution and Refinement

The structure of AbyV was determined by molecular replacement in MOLREP,[2]using chain A from CYP105D6 as a search model (PDB ID 3ABB).[3] This initial AbyV model was rebuilt and refined using a combination of COOT[4] and REFMAC[5]. The final model comprises 11-395 of the native sequence in Chain A, and 27-395 of the native sequence in Chain B, 2 heme prosthetic groups (1 per chain), 4 molecules of glycerol, 9 of polyethylene glycol (PEG), 1

chloride ion, 5 magnesium ions and 145 molecules of water. Electron density was not observed in the regions of both Chain A and Chain B corresponding to the AbyV BC loop (81-87 in chain A, 73-94 in chain B). Similarly, density was not observed for residues 372-384 of chain B, indicative of structural disorder. Data collection, phasing and refinement statistics for AbyV are provided in table S1. The structure of AbyV has been deposited in the PDB with code 7QAN. Protein structure figures have been prepared using PYMOL.[6]

1.5 Spectrophotometric Analysis of Compound 3 Binding to AbyV

The substrate dissociation constant (K_d) of **3** for AbyV was determined using absorbance spectroscopy by monitoring the displacement of water as the sixth ligand to the heme iron at 390 nm. All experiments were performed using a Varian Cary 50 spectrophotometer, by titrating aliquots of compound **3 (**0-160 μM final concentration) into a 1 mL buffered solution of 10 μM AbyV. All spectra were recorded using a 1 cm path length quartz cuvette in buffer comprising 20 mM Tris-HCl, 150 nM NaCl, pH 7.5, at a temperature of 25°C. Absorbance values at 390 nm were plotted against substrate concentration and fitted to a rectangular hyperbolic function by non-linear regression analysis using GraphPad Prism 8.

1.6 AbyV Epoxidation Activity Assays

In a total reaction volume of 200 µL (in potassium phosphate buffer (50 mM), at pH 7.5, with 1% acetonitrile), **3** or (12-^{13}C)-**3** (1 mM) was incubated with AbyV (10 µM), spinach ferredoxin-NADP$^+$ reductase (0.05 units, Sigma Aldrich), spinach ferredoxin (0.015 mg, Sigma Aldrich) and NADPH (1 mM, Sigma Aldrich) at 25 °C. At the appropriate time point the reaction was terminated by the addition of ice-cold acetonitrile (200 µL) and the reaction mixture clarified by centrifugation (14000 rpm, 4 min). The reaction mixture was then extracted with EtOAc (2 x 400 µL), and the organic extract dried under a stream of nitrogen. Control reactions were performed according to the same conditions but either without AbyV or with only **3** in buffer. Reactions for *in situ* NMR monitoring were performed according to the same conditions with 10% D_2O added to the mixture prior to transfer to an NMR tube.

No evidence of peroxide damage to AbyV was observed over the timeframe of the assays, as would be indicated by sample aggregation.

1.7 Analysis of AbyV Epoxidation Activity Assays

1.7.1 LC-MS

The dried assay extract was dissolved in acetonitrile (80 µL) and this solution was subjected to LC-MS analysis using a Waters 2445SFO HPLC system with a Waters 2298 diode array detector for UV between 200 and 400 nm. The system was equipped with a Phenomenex LUNA column (5 µm, C18, 100 Å, 4.6 × 250 mm) eluting with a linear gradient of 5-95% MeCN in H_2O with 0.05 % formic acid; flow rate: 1 $mL.min^{-1}$; detection: 254 nm; Mass spectrometry was performed using a Waters QM ESI spectrometer in positive and negative modes, with detection between 150 and 1200 m/z units.

1.7.2 ^{1}H NMR

The dried assay extract was dissolved in acetonitrile and purified by semi-preparative HPLC using a Waters 2445SFO HPLC system with a Waters 2298 diode array detector for UV between 200 and 400 nm. The system was equipped with a Phenomenex LUNA column (5 µm, C18, 100 Å, 4.6 × 250 mm) eluting with a linear gradient of 5-95% MeCN in H_2O with 0.05 % formic acid; flow rate: 1 $mL.min^{-1}$; detection: 254 nm; Collection of the product at t_R = 9.0 allowed an analytical sample of the proposed epoxide product to be isolated. The sample was dried under a stream of nitrogen and dissolved in methanol-d_4 and subjected to ^{1}H NMR analysis on a Bruker Avance III HD 700 spectrometer.

1.7.3 ^{13}C NMR

The dried assay extract (from assays conducted with (12-^{13}C)**3**) was dissolved in methanol-d_4 (500 µL) and subjected to ^{13}C NMR analysis on a Bruker Avance II HD 500 Cryo spectrometer.

1.7.4 *In situ* Monitoring

Reactions for *in situ* NMR monitoring were performed according to the conditions outlined in **1.6** with 10% D_2O added to the mixture prior to transfer to an NMR tube. ^{13}C (DEPT) analyses were carried out on a Bruker Avance III HD 700 spectrometer.

1.8 Docking

Molecular docking was performed to obtain initial poses of the two atropisomer substrates within the AbyV active site that can result in epoxidation of **3** at C-11/C-12. Conformations of the two atropisomers of **3** were those previously optimized with DFT at the B97D/6-31+G(d,p) level.[7] Docking into chain A of the AbyV structure was then performed using AutoDock Vina.[8] AutoDockTools 1.5.6 was first used to assign AutoDock atom types, remove non-polar hydrogens from the ligand and add polar hydrogens to the protein (with all residues in their standard protonation states). Both protein and ligand were treated as rigid during docking (and no oxygen was attached to the heme iron). Docking was then performed using a search grid of size 21.4 x 16.5 x 17.6 Å centered on the active site, and an exhaustiveness of 16, obtaining 20 poses for each substrate. For each atropisomer, a single pose was selected based on the best scoring pose with a distance between the compound I oxygen and the epoxidation site carbons <4 Å.

1.9 AbyV Chain A Loop Modelling

The flexible BC loop, largely not observed in the crystal structure, was modelled into chain A of the AbyV crystal structure to generate a complete structure for MD simulation. Loop modelling (and optimisation) for residues 80-88 was performed using MODELLER 9.20.[9,10] 200 loop models were generated and refined using the second highest MD refinement level. To obtain a loop conformation consistent with the loop being highly flexible, we considered the top 20 conformations based on the molpdf score and selected the loop model with the best DOPE score that did not feature the loop making close interactions with the interior of the protein. (Considering DOPE score alone results in loop models that are buried and in contact the with the F,G and I helices, which is inconsistent with expected flexible loop indicated by the X-ray data).

1.10 Molecular Dynamics Setup, Simulations and Analysis

The selected docked poses for each atropisomer of **3** were combined with chain A of the AbyV structure (including crystallographic water molecules) and the coordinates for residues 80-88 obtained from MODELLER. Water molecules within 2 Å of the substrate or the remodelled loop were deleted. The Enlighten PREP protocol (see: www.github.com/marcvanderkamp/enlighten)[11] was used to prepare the structures for simulation. This includes addition of hydrogens to the protein (incl. Asn/Gln/His flips were appropriate) using the AmberTools[12] programme reduce and based on pK_a prediction from PropKa 3.1.[13,14] His339 and His342 were singly protonated at Ne2 and all other histidines singly protonated at Nd1. All other titratable residues were modelled in their standard states, apart from Glu353, which was protonated (based on a predicted pK_a of 9.54). A 30 Å solvent sphere was then added around the active site (with the AmberTools program tleap), centred on carbon atom of the tetronate ring to which the hydroxy group is attached. In subsequent simulations, the protein was treated by the ff14SB force field[15] and water using the TIP3P model. AM1-BCC partial charges and Generalised Amber Force Field[16] parameters were generated for each atropisomer substrate using Antechamber. For the heme and Cys344 to which it is bound, Amber compatible parameters[17] consistent with compound I were used.

The Enlighten protocols STRUCT and DYNAM were then applied (using sander from AmberTools16, with the entire structure treated with MM). The STRUCT protocol performs brief simulated annealing and minimisation to optimise the structure, with atoms within 25 Å of the solvent sphere centre allowed to move. DYNAM then continues and performs brief heating to 300K, followed by molecular dynamics (MD) simulation, with all atoms within the 30 Å solvent sphere allowed to move. DYNAM was repeated 10 times to obtain 10 independent 200ps MD trajectories (2 fs timestep) for each atropisomer. The percentage of reactive conformations was calculated from structures at every 1 ps in the last 100 ps of the 10 MD trajectories (1000 snapshots per atropisomer). A snapshot was deemed reactive if the two geometric reactivity criteria established in a previous study[18] (to identify structures from which

epoxidation may readily occur) were satisfied for either carbon of the double bond of the substituted cyclohexene. These two conditions are that the distance between the epoxidation site carbon and the oxygen of compound I be less than 4 Å, and that the angle between the compound I iron, oxygen and the epoxidation site carbon atom be between 110 and 140°.

1.11 Compound Synthesis

1.11.1 General Considerations

All reagents were obtained from commercial suppliers and used without further purification. All air and moisture sensitive reactions were carried out using standard Schlenk syringe-septa techniques, using flame dried glassware under a positive pressure of nitrogen. Anhydrous THF, CH_2Cl_2, Et_2O and toluene were dried by passing through a modified Grubbs system of alumina columns, manufactured by Anhydrous Engineering, and stored over 3 Å molecular sieves. Anhydrous DMSO, DMF, acetone, MeOH and pyridine were obtained from commercial suppliers and used without further drying. DIPA and NEt_3 were distilled over CaH_2 prior to use. All stated temperatures below ambient are the temperatures of the cooling baths. TLC analysis was performed with aluminium backed silica TLC plates (Merck-Kieselgel 60 F_{254}) using the stated solvent system and the plates were visualised using UV fluorescence (254 nm) and/or developed with potassium permanganate solution. Flash column chromatography was performed according to the procedures described by Still,[19] using silica gel 60 (Fisher Scientific or Aldrich) and the stated solvent system.

1.11.2 Compound Characterisation

^{1}H and ^{13}C NMR spectra were recorded using Jeol ECZ 400, Jeol ECS 400, Bruker Nano 400 and Bruker Avance III HD 500 Cryo spectrometers at ambient temperature. Chemical shifts (δ) are quoted in parts per million (ppm) and coupling constants (*J*) are in Hertz (Hz) rounded to 0.5 Hz intervals. Residual solvent peaks were used as the internal reference for proton and carbon chemical shifts. Two-dimensional NMR techniques (HSQC, COSY, HMBC) were used

routinely for structural assignment. HRMS ESI were performed on either a Bruker Daltonics Apex 4, 7 Tesla FTICR or microTOF II. Samples were submitted in MeOH or CH_2Cl_2. Specific rotations ($[\alpha]_D^T$)were measured on a Bellingham and Stanley Ltd. ADP220 polarimeter and are quoted in (° ml)(g dm)$^{-1}$. Infra-red spectra were recorded on a Perkin Elmer Spectrum 100 FTIR with an ATR accessory and frequencies are reported in wavenumbers (cm^{-1}).

1.11.3 Synthesis of AbyV Substrate 3 and Epoxide 4

(*S*)-3-Bromo-1-(*tert*-butyldimethylsilyloxy)-2-methylpropane 24

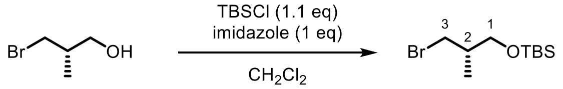

23 **24**

Under an atmosphere of nitrogen, *(S)*-3-bromo-2-methyl-1-propanol **23** (1 g, 6.58 mmol) was dissolved in CH_2Cl_2 (20 mL). TBSCl (1.10 g, 7.24 mmol) and imidazole (0.67 g, 9.87 mmol) were added, and the reaction was stirred for 4 h at room temperature. The reaction was quenched with sat. aq. NH_4Cl (20 mL), and the aqueous layer was extracted with CH_2Cl_2 (3 × 20 mL). The combined organic layers were washed with brine (30 mL), dried ($MgSO_4$), filtered and the solvent removed *in vacuo*. The crude residue was dissolved in toluene (20 mL) and the solvent removed *in vacuo* to give bromide **73** as a colourless oil (1.65 g, 93%). $[\boldsymbol{\alpha}]_{\mathbf{D}}^{\mathbf{20}}$= + 7.8 (*c* 0.9, $CHCl_3$) (Lit. $[\alpha]_D^{27}$= + 11.1 (*c* 1.42, CH_2Cl_2)).[20] **δ^H (400 MHz, $CDCl_3$)** 3.57 (1H, dd, *J* 10.0, 5.0, 1-*H*H), 3.51 – 3.42 (3H, overlapping m, 1-H*H* & 3-H_2), 1.99 – 1.95 (1H, m, 2-H), 0.99 (3H, d, *J* 7.0, 2-CH_3), 0.89 (9H, s, $SiC(CH_3)_3$), 0.06 (6H, s, $Si(CH_3)_2$); **δ^C (100 MHz, $CDCl_3$)** 65.5 (C-2), 38.2 (C-3), 37.9 (C-1), 26.0 (Si(C(*C*H$_3$)$_3$), 18.4 (Si(*C*(CH$_3$)$_3$), 15.6 (2-CH_3), -5.3 (Si(*C*H$_3$)$_2$).

All data are in accordance with the literature.[20]

(*S*)-3-Iodo-1-(*tert*-butyldimethylsilyloxy)-2-methylpropane 25

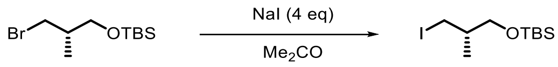

24 **25**

24 (1.74 g, 6.51 mmol) was dissolved in acetone (18 mL), and sodium iodide (3.88 g, 26.0 mmol) was added. The reaction was heated at reflux for 16 h and then cooled to room temperature. The solvent was removed *in vacuo* and the crude residue was dissolved in EtOAc (20 mL). H_2O (20 mL) was added, the layers separated, and the aqueous layer extracted with EtOAc (3 × 30 mL). The combined organic layers were dried ($MgSO_4$), filtered and the solvent removed *in vacuo* to give silyl ether **25** as a yellow oil (2.05 g, quant.). $[\boldsymbol{\alpha}]_{\mathbf{D}}^{\mathbf{20}}$= + 7.5 (*c* 0.8, $CHCl_3$) (Lit. $[\alpha]_D^{23}$= + 12.2 (*c* 1.4, $CHCl_3$)).[21] **δ^H (400 MHz, $CDCl_3$)** 3.53 (1H, dd, *J* 10.0, 5.0, 1-*H*H), 3.40 (1H, dd, *J* 10.0, 7.0, 1-H*H*), 3.30 (1H, dd, *J* 9.5, 5.5, 3-*H*H), 3.25 (1H, dd, *J* 9.5, 5.5, 3-H*H*), 1.66 – 1.61 (1H, m, 2-H), 0.95 (3H, d, *J* 6.5, CH_3), 0.90 (9H, s, $SiC(CH_3)_3$), 0.06 (6H, s, $Si(CH_3)_2$). **δ^C (100 MHz, $CDCl_3$)** 66.8 (C-1), 37.5 (C-2), 26.0 ($SiC(CH_3)_3$), 18.4 ($SiC(CH_3)_3$), 17.4 (2-CH_3), 14.0 (C-3), -5.2 ($Si(CH_3)_2$). **HRMS (APCI+)** calc. for $[C_{10}H_{22}OISi+H]^+$ 315.0636, found 315.0629.

All data are in accordance with the literature.[21]

(3*S*, 5*R*)-3,5-Dimethyltetrahydro-2H-pyran-2-one 16

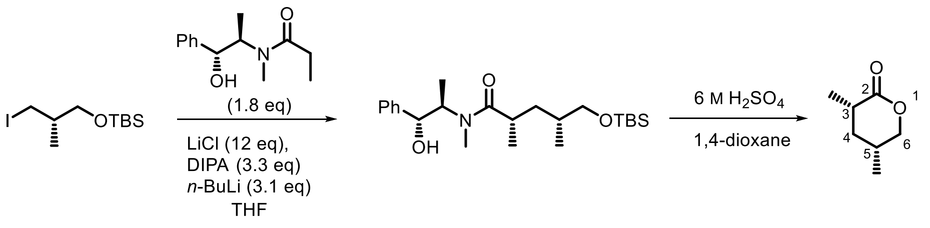

25 **26** **16**

A RBF containing lithium chloride (3.04 g, 71.64 mmol) was flame dried under vacuum for 5 minutes and the flask was then allowed to cool to room temperature under an atmosphere of nitrogen. Anhydrous THF (17 mL) was added followed by diisopropylamine (2.77 mL, 19.70

mmol) and the solution cooled to −78 °C. *n*-BuLi (1.39 M, 13.3 mL, 18.51 mmol) was added dropwise, the mixture was raised to 0 °C and held for 5 minutes before cooling back to −78 °C. Pseudoephedrine propionamide[22] (2.37 g, 10.74 mmol) in anhydrous THF (17 mL) was then added to the LDA solution and the resulting mixture was stirred at −78 °C for 1 h, 30 minutes at 0 °C, 5 minutes at room temperature before being cooled back to 0 °C. At 0 °C, iodide **25** (1.90 g, 5.97 mmol) in anhydrous THF (11 mL) was added slowly to the stirred solution which was then allowed to warm to room temperature and stirred overnight. The reaction was quenched with sat. aq. NH_4Cl (40 mL) and the aqueous layer was extracted with EtOAc (4 × 50 mL). The combined organic layers were dried ($MgSO_4$), filtered and the solvent removed *in vacuo*. The crude residue was purified by column chromatography, eluting with 30-50% EtOAc in petroleum ether (40-60 °C) to give the auxiliary adduct **26** as a pale-yellow oil (2.43 g, 94%). $[\boldsymbol{\alpha}]_{\mathbf{D}}^{\mathbf{20}}$= -36.0 (*c* 1.0, $CHCl_3$) (Lit. $[\alpha]_{D}^{25}$= - 58.1 (*c* 1.2, CH_2Cl_2)).[23] The auxiliary adduct (2.23 g, 5.47 mmol) was dissolved in 1,4-dioxane (55 mL) and the solution cooled to 0 °C. 6 M H_2SO_4 (55 mL) was added slowly and the flask was warmed to 30 °C and stirred overnight. EtOAc (50 mL) was added, the layers separated, and the aqueous layer extracted with EtOAc (4 × 30 mL). The combined organic layers were washed sequentially with sat. aq. $NaHCO_3$ (30 mL) and brine (20 mL), dried ($MgSO_4$), filtered and the solvent removed *in vacuo*. The crude residue was purified by column chromatography, eluting with 20% EtOAc in petroleum ether (40-60 °C) to give lactone **16** as a colourless solid (530 mg, 76%). $[\boldsymbol{\alpha}]_{\mathbf{D}}^{\mathbf{20}}$= + 33.8 (*c* 0.8, $CHCl_3$) (Lit. $[\alpha]_{D}^{27}$= + 41.6 (*c* 0.1, $CHCl_3$)).[24] **δ^H (400 MHz, $CDCl_3$)** 4.31 (1H, ddd, *J* 11.0, 5.0, 2.0, 6-*H*H), 3.88 (1H, dd, *J* 11.0, 9.5, 6-H*H*), 2.52 (1H, dqd, *J* 12.5, 7.0, 6.0, 3-H), 2.20 – 2.04 (2H, m, 4-H_2), 1.25 (3H, d, *J* 7.0, 3-CH_3), 1.23 (1H, m, 5-H) 0.98 (3H, d, *J* 6.5, 5-CH_3)). **δ^C (100 MHz, $CDCl_3$)** 174.7 (C-2), 75.1 (C-6), 37.0 (C-4), 35.5 (C-3), 28.8 (C-5), 17.5 (CH_3), 17.1 (CH_3). **HRMS (APCI+)** calc. for $[C_7H_{11}O_2+H]^+$ 129.0910, found 129.0905. **IR (v_{max}/cm^{-1})** (neat) 2962, 1731, 1460.

All data are in accordance with the literature.[24]

(*E*)-(2-^{13}C)Crotonic acid 27

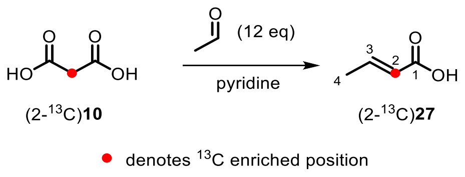

(2-^{13}C)Malonic acid (3 g, 28.56 mmol) was dissolved in pyridine (30 mL) and the solution was cooled to 0 °C. Acetaldehyde (19.25 mL, 342.72 mmol) was added and the solution was allowed to warm to room temperature and then stirred for 16 h. The solution was then heated to 50 °C for 4 h before cooling to room temperature. Water (20 mL) was added, and the solution acidified to pH 1.5 with conc. HCl. The aqueous layer was extracted with Et_2O (4 × 100 mL), the combined organic layers dried ($MgSO_4$), filtered and the solvent removed *in vacuo* to give (2-^{13}C)**27** as a white solid (2.39 g, 96%). **δ^H (400 MHz, $CDCl_3$)** 7.10 (1H, dqd, *J* 15.5, 7.0, 2.0, 3-H), 5.86 (1H, ddq, *J* 163.0, 15.5, 1.5, 2-H), 1.92 (3H, ddd, *J* 7.0, 7.0, 1.5, 4-H_3). **δ^C (100 MHz, $CDCl_3$)** 171.6 (d, *J* 72.5, C-1), 147.6 (d, *J* 70.0, C-3), 122.2 (C-2 (enhanced)), 18.3 (d, *J* 1.5, C-4). **HRMS (+APCI)** calc. for $[C_3{}^{13}CH_6O_2+H]^+$ 88.0474, found 88.0472.

All data in accordance with the literature.[25]

Ethyl (*E*)-(2-^{13}C)crotonate 11

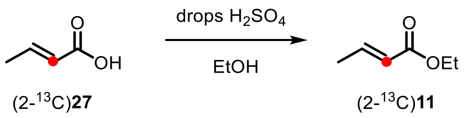

(2-^{13}C)Crotonic acid (2.39 g, 27.44 mmol) was dissolved in ethanol (50 mL) and the solution cooled to 0 °C. Conc. H_2SO_4 (4 drops) was added, and the solution heated at reflux for 16 h. The reaction was cooled to 0 °C, quenched with sat. aq. $NaHCO_3$ (20 mL) and the aqueous layer extracted with CH_2Cl_2 (3 × 60 mL). The combined organic layers were washed with water (3 × 30 mL) and brine (1 × 30 mL), dried ($MgSO_4$), filtered and the solvent removed *in vacuo* to give (2-^{13}C)**11** as a colourless oil (2.63 g, 83%). **δ^H (400 MHz, $CDCl_3$)** 6.97 (1H, dqd, *J* 15.5,

7.0, 2.0, 3-H), 5.84 (1H, ddq, *J* 162.0, 15.5, 1.5, 2-H), 4.18 (2H, q, *J* 7.0, OC$H_2$$CH_3$), 1.87 (3H, td, *J* 7.0, 1.5, 4-H_3), 1.28 (3H, t, *J* 7.0, OCH_2CH_3). **δ^C (100 MHz, $CDCl_3$)** 166.7 (d, *J* 75.0, C-1), 144.6 (d, *J* 70.5, C-3), 123.0 (C-2 (enhanced)), 60.2 (O$CH_2$$CH_3$), 18.1 (d, *J* 1.5, C-4), 14.4 (O$CH_2$$CH_3$). **HRMS (+APCI)** calc. for $[C_5{}^{13}CH_{10}O_2+H]^+$ 116.0787, found 116.0784.

All data in accordance with the literature.[26]

Ethyl (2*E*, 4*E*)-(4-^{13}C)hexadienoate 14

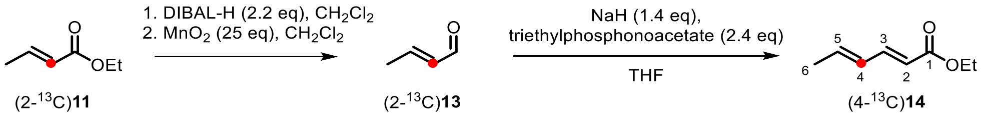

Under an atmosphere of nitrogen, (2-^{13}C)**11** (2.60 g, 22.58 mmol) was dissolved in CH_2Cl_2 (120 mL) and cooled to – 40 °C. DIBAL-H (1.0 M in hexanes, 50 mL, 50 mmol) was added dropwise and the reaction was allowed to warm to room temperature and stirred for 1 h. The reaction was cooled to 0 °C and sat. aq. potassium sodium tartrate (80 mL) was added. The resulting cloudy suspension was stirred vigorously until biphasic, and the aqueous layer extracted with CH_2Cl_2 (3 × 100 mL), the organic layers combined, washed with brine (50 mL), dried ($MgSO_4$), filtered and concentrated *in vacuo* to a volume of 75 mL. MnO_2 (49.0 g, 564.5 mmol) was added to the solution of crotyl alcohol, and the suspension was stirred at room temperature for 1 h. The suspension was filtered through a pad of celite and the solvent was removed *in vacuo* to give crotonaldehyde (2-^{13}C)**13** as a colourless liquid (**δ^H (400 MHz, $CDCl_3$)** 9.50 (1H, dd, *J* 25.5, 8.0, 1-H), 6.86 (1H, dqd, *J* 15.5, 7.0, 0.5, 3-H), 6.15 (1H, ddq, *J* 160.5, 15.5, 1.5, 2-H), 2.03 (3H, dd, *J* 7.0, 1.5, 4-H_3), which was used immediately in the following step.

Under an atmosphere of nitrogen, NaH (60% suspension in mineral oil, 758 mg, 31.61 mmol) was dissolved in anhydrous THF (150 mL). The solution was cooled to 0 °C and triethyl phosphonoacetate (10.75 mL, 54.19 mmol) was added dropwise. The solution was stirred at the same temperature for 0.5 h and then previously prepared crotonaldehyde (2-^{13}C)**13** was

added dropwise. The reaction was allowed to warm to room temperature and stirred for 16 h. The reaction was quenched by the addition of sat. aq. NH_4Cl (70 mL) and the aqueous layer was extracted with EtOAc (3 × 100 mL). The combined organic layers were dried ($MgSO_4$), filtered and the solvent removed *in vacuo*. The crude residue was purified by column chromatography, eluting with 5% EtOAc in petroleum ether (40-60 °C) to give (4-^{13}C)**14** as a colourless oil (0.78 g, 24% over 3 steps). **δ^H (400 MHz, $CDCl_3$)** 7.25 (1H, ddd, *J* 15.0, 11.0, 2.5, 3-H), 6.19 (1H, ddd, *J* 154.5, 15.0, 11.0, 4-H), 6.13 (1H, dq, *J* 15.0, 7.0, 5-H), 5.77 (1H, dd, *J* 15.5, 7.0, 2-H), 4.19 (2H, q, *J* 7.0, OCH_2CH_3), 1.85 (3H, td, *J* 7.0, 1.5, 6-H_3), 1.29 (3H, t, *J* 7.0, OCH_2CH_3). **δ^C (100 MHz, $CDCl_3$)** 167.5 (d, *J* 8.0, C-1), 145.0 (d, *J* 55.5, C-3), 139.3 (d, *J* 70.0, C-5), 129.9 (C-4 (enhanced)), 119.2 (d, *J* 1.0, C-2), 60.3 (OCH_2CH_3), 18.8 (d, *J* 1.0, C-6), 14.5 (OCH_2CH_3). **HRMS (+APCI)** calc. for $[C_7{}^{13}CH_{12}O_2+H]^+$ 142.0941, found 142.0941.

All data in accordance with the literature.[27]

(2*E*, 4*E*)-(4-^{13}C)Hexadienal 15

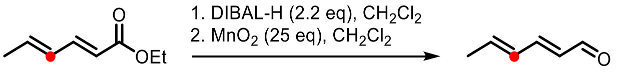

(4-^{13}C)**14** (4-^{13}C)**15**

Under an atmosphere of nitrogen, (4-^{13}C)**14** (745 mg, 5.28 mmol) was dissolved in CH_2Cl_2 (28 mL) and the solution cooled to – 40 °C. DIBAL-H (1.0 M in hexanes, 12.7 mL, 12.7 mmol) was added dropwise and the reaction was allowed to warm to room temperature and stirred for 1 h in the absence of light. The reaction was cooled to 0 °C and sat. aq. potassium sodium tartrate (20 mL) was added. The resulting cloudy suspension was stirred vigorously until biphasic and the aqueous layer extracted with CH_2Cl_2 (3 × 30 mL), the organic layers were combined, washed with brine (50 mL), dried ($MgSO_4$), filtered and the solvent removed *in vacuo* to give (2*E*, 4*E*)-(4-^{13}C)hexadienol as a colourless oil (**δ^H (400 MHz, $CDCl_3$)** 6.20 (1H, m, 3-H), 6.06 (1H, ddd, *J* 151.5, 15.5, 10.5, 4-H), 5.72 (2H, overlapping m, 2-H and 5-H), 4.15 (2H, d, *J* 5.5, 1-H_2), 1.76 (3H, dd, *J* 7.5, 6.5, 6-H_3)) which was used immediately in the next

step. (2*E*, 4*E*)-(4-^{13}C)hexadienol (480 mg, 4.84 mmol) was dissolved in CH_2Cl_2 (16 mL) and MnO_2 (10.50 g, 120.7 mmol) was added. The resulting suspension was stirred at room temperature for 1 h in the absence of light. The suspension was filtered through a pad of celite, and the solvent was removed *in vacuo* to give (4-^{13}C)**15** as a yellow oil (359 mg, 72% over 2 steps). **δ^H (400 MHz, $CDCl_3$)** 9.53 (1H, d, *J* 8.0, 1-H), 7.08 (1H, ddd, *J* 15.5, 11.0, 2.1, 3-H), 6.34 (1H, ddd, *J* 153.5, 15.5, 11.0, 4-H), 6.29 (1H, dqd, *J* 15.5, 7.0, 2.0, 5-H), 6.06 (1H, m, 2-H), 1.91 (3H, dd, *J* 8.0, 7.0, 6-H_3). **δ^C (100 MHz, $CDCl_3$)** 194.1 (C-1), 152.7 (d, *J* 55.5, C-3), 142.0 (d, *J* 69.5, C-5), 130.3 (C-4 (enhanced)), 130.1 (C-2), 19.1 (C-6). **HRMS (+APCI)** calc. for $[C_5{}^{13}CH_8O+H]^+$ 98.0681, found 96.0680.

All data in accordance with the literature.[28]

(2*R*,4*S*,6*E*,8*E*,10*E*)-2,4-Dimethyl-1-hydroxy-dodeca-6,8,10-trien-5-one 18

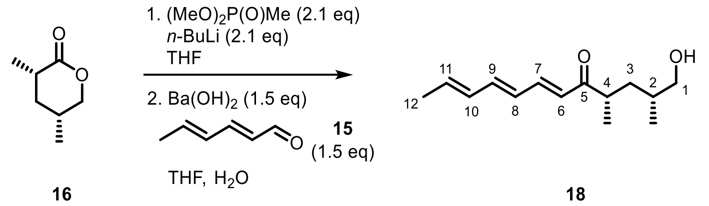

Under an atmosphere of nitrogen, dimethyl methylphosphonate (2.14 mL, 19.83 mmol) was dissolved in anhydrous THF (32 mL) and the solution cooled to -78 °C. *n*-BuLi (1.5 M, 13.2 mL, 19.80 mmol) was added dropwise, and the mixture was stirred at the same temperature for 0.5 h. Lactone **16** (1.21 g, 9.44 mmol) in anhydrous THF (20 mL) was added dropwise, and the reaction was stirred at - 78 °C for 1 h. The reaction was quenched by the addition of sat. aq. NH_4Cl (60 mL), and the aqueous layer was extracted with EtOAc (5 × 30 mL). The combined organic layers were dried (Na_2SO_4), filtered and the solvent removed *in vacuo*. The crude residue was dissolved in THF (150 mL) and cooled to 0 °C. Barium hydroxide (2.43 g, 14.16 mmol) and (*2E, 4E*)-hexa-2,4-dienal (1.86 mL, 14.16 mmol) were added. H_2O (30 mL) was added dropwise, and the reaction was stirred at room temperature for 16 h. The reaction was quenched by the addition of sat. aq. NH_4Cl (20 mL), and the aqueous layer was extracted

with EtOAc (3 × 20 mL). The combined organic layers were dried (Na_2SO_4), filtered and the solvent removed *in vacuo*. The crude residue was purified by column chromatography, eluting with 15-30% EtOAc and 0.05% Et_3N in petroleum ether (40-60 °C), giving alcohol **18** as a yellow oil (1.47 mg, 70%). $[\alpha]_D^{24}$= + 17.5 (*c* 0.4, $CHCl_3$) (Lit. $[\alpha]_D^{21}$= + 22.4 (*c* 0.25, $CHCl_3$)).[29] **δ^H (400 MHz, CD_3OD)** 7.32 (1H, dd, *J* 15.5, 11.0, 7-H), 6.70 (1H, dd, *J* 15.0, 11.0, 9-H), 6.37 – 6.20 (3H, m, 6-H, 8-H and 10-H), 6.03 (1H, m, 11-H), 3.40 (1H, dd, *J* 11.0, 5.0, 1-*H*H), 3.31 (1H, dd, *J*, 11.0, 5.0, 1-H*H*), 3.00 (1H, m, 4-H), 1.83 (3H, d, *J* 7.5, 12-H_3), 1.81 (1H, m, 3-*H*H), 1.54 (1H, m, 2-H), 1.11 (1H, m, 3-H*H*), 1.09 (3H, d, *J* 7.0, 4-CH_3), 0.91 (3H, d, *J* 7.0, 2-CH_3). **δ^C (100 MHz, CD_3OD)** 206.9 (C-5), 145.3 (C-7), 144.1 (C-9), 136.7 (C-11), 132.8 (C-10), 129.4 (C-8), 128.3 (C-6), 68.3 (C-1), 42.8 (C-4), 38.4 (C-3), 34.9 (C-2), 18.7 (C-12), 18.3 (4-CH_3), 17.3 (2-CH_3). **HRMS (ESI)** calc. for $[C_{14}H_{22}O_2+H]^+$ 223.1693, found 223.1688. **IR (ν_{max}/cm^{-1})** (neat) 3428, 2962, 2878, 1674, 1596, 1574.

All data are in accordance with the literature.[29]

The above procedure was repeated with (4-^{13}C)**15** (359 mg, 3.70 mmol, 0.7 eq), giving (10-^{13}C)**18** (261 mg, 32% over 2 steps). **δ^H (400 MHz, CD_3OD)** 7.32 (1H, dd, *J* 15.0, 11.5, 7-H), 6.69 (1H, dd, *J* 15.0, 11.0, 9-H), 6.31 (2H, m, 6-H and 8-H), 6.23 (1H, dddd, 158.5, 15.0, 11.0, 1.5, 10-H), 6.03 (1H, m, 11-H), 3.39 (1H, dd, *J* 10.5, 5.5, 1-*H*H), 3.31 (1H, dd, *J* 10.5, 5.5, 1-H*H*), 3.00 (1H, m, 4-H), 1.83, (3H, dd, J 8.0, 6.0, 12-H_3), 1.80 (1H, m, 3-*H*H), 1.53 (1H, m, 2-H), 1.12 (1H, m, 3-H*H*), 1.09 (3H, d, *J* 7.0, 4-CH_3), 0.91 (3H, d, *J* 6.5, 2-CH_3). **δ^C (100 MHz, CD_3OD)** 206.9 (C-5), 145.3 (d, *J* 8.0, C-7), 144.1 (d, *J* 55.5, C-9), 136.7 (d, *J* 70.0, C-11), 132.8 (C-10 (enhanced)), 129.4 (C-8), 128.3 (C-6), 68.3 (C-1), 42.9 (C-4), 38.4 (C-3), 34.9 (C-2), 18.7 (C-2), 18.3 (4-CH_3), 17.3 (2-CH_3). **HRMS (ESI)** calc. for $[C_{13}{}^{13}CH_{22}O_2+H]^+$ 224.1726, found 224.1725.

(2*R*,4*S*,6*E*,8*E*,10*E*)-2,4-Dimethyl-5-oxododeca-6,8,10-trienal 19

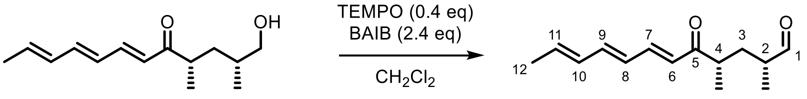

Under an atmosphere of nitrogen, alcohol **18** (263 mg, 1.18 mmol) was dissolved in CH_2Cl_2 (6.5 mL) and the solution was cooled to 0 °C. (Diacetoxyiodo)benzene (911 mg, 2.83 mmol) and TEMPO (73 mg, 0.47 mmol) were added, and the solution was stirred at room temperature for 3 h. The reaction was quenched by the addition of sat. aq. $Na_2S_2O_3$ (5 mL) and sat. aq. $NaHCO_3$ (1 mL) and then stirred for a further 30 minutes. The layers were separated, and the aqueous layer was extracted with CH_2Cl_2 (3 × 15 mL). The combined organic layers were washed with brine (30 mL), dried ($MgSO_4$), filtered and the solvent removed *in vacuo*. The crude residue was purified by column chromatography, eluting with 10-15% EtOAc in petroleum ether (40-60 °C) to give aldehyde **19** as a yellow oil (212 mg, 81%). $[\boldsymbol{\alpha}]_{\mathbf{D}}^{\mathbf{21}}$= + 4.0 (*c* 1, $CHCl_3$) (Lit. $[\alpha]_{D}^{22}$= + 5.7 (*c* 0.25, $CHCl_3$)).[29] **δ^H (400 MHz, $CDCl_3$)** 9.59 (1H, d, *J* 1.5, 1-H), 7.26 (1H, m, 7-H), 6.60 (1H, ddd, *J* 14.5, 11.0, 4.0, 9-H), 6.26 – 6.12 (3H, m, 6-H, 8-H and 10-H), 5.97 (1H, dq *J* 14.5, 7.0, 11-H), 2.97 – 2.73 (1H, m, 4-H), 2.43 – 2.32 (1H, m, 2-H), 2.19 (1H, app. dt, *J* 14.0, 7.5, 3-*H*H), 1.84 (3H, d, *J* 7.0, 12-H_3), 1.35 – 1.24 (1H, m, 3-H*H*), 1.12 (3H, d, *J* 7.0, 4-CH_3), 1.09 (3H, d, *J* 7.0, 2-CH_3). **δ^C (100 MHz, $CDCl_3$)** 204.6 (C-1), 203.0 (C-5), 143.7 (C-7), 142.7 (C-9), 136.0 (C-11), 131.5 (C-10), 128.1 (C-8), 127.0 (C-6), 44.4 (C-2), 41.7 (C-4), 33.6 (C-3), 18.8 (C-12), 17.7 (CH_3), 13.9 (CH_3). **HRMS (ESI)** calc. for $[C_{14}H_{20}O_2+H]^+$ 221.1536, found 221.1533. **IR (ν_{max}/cm^{-1})** (neat) 2968, 2875, 2712, 1722, 1596, 1573.

All data are in accordance with the literature.[29]

The above procedure was repeated with (10-^{13}C)**18** (222 mg, 0.99 mmol), giving (10-^{13}C)**19** (136 mg, 62% yield). **δ^H (400 MHz, $CDCl_3$)** 9.60 (1H, d, *J* 2.0, 1-H), 7.26 (1H, dd, *J* 16.0, 11.5 , 7-H), 6.60 (1H, dd, *J* 15.5, 10.0, 9-H), 6.17 (1H, dddd, *J* 153.0, 15.5, 11.0, 2.0, 10-H), 6.23 (2H, m, 6-H and 8-H), 5.99 (1H, m, 11-H), 2.86 (1H, m, 4-H), 2.38 (1H, m, 2-H), 2.20 (1H, ddd,

J 14.0, 7.0, 6.0, 3-*H*H), 1.84 (3H, dd, *J* 7.5, 6.0, 12-H_3), 1.31 (1H, ddd, *J* 14.0, 7.0, 6.0, 3-H*H*), 1.14 (3H, d, *J* 7.0, 4-CH_3), 1.09 (3H, d, *J* 7.0, 2-CH_3). **δ^C (100 MHz, $CDCl_3$)** 204.6 (C-1), 203.0 (C-5), 143.7 (C-7), 142.7 (d, *J* 56.5, C-9), 135.9 (d, *J* 70.5, C-11), 131.4 (C-10 (enhanced)), 128.1 (C-8), 127.0 (C-6), 44.4 (C-2), 41.7 (C-4), 33.6 (C-3), 18.7 (C-12), 17.7 (CH_3), 13.9 (CH_3). **HRMS (ESI)** calc. for $[C_{13}{}^{13}CH_{20}O_2+H]^+$ 222.1569, found 222.1571.

(2*R*,4*S*,6*E*,8*E*,10*E*)-1-(4′-Methoxy-5′-methylene-2′-oxo-2′,5′-dihydrofuran-3′-yl)-2,4-dimethyldodeca-6,8,10-triene-1,5-dione 20

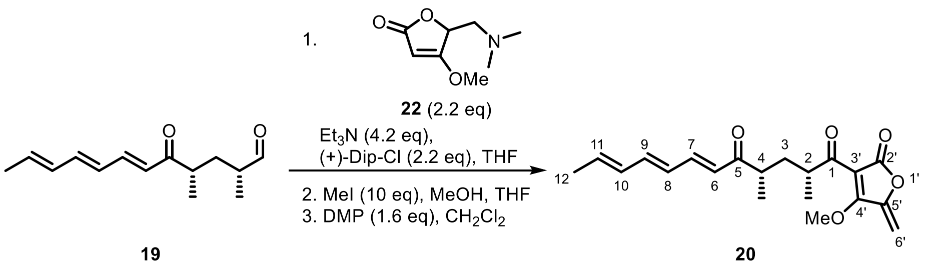

19 20

Two identical reactions were carried out in parallel as follows: Under an atmosphere of nitrogen, **22** (287 mg, 1.66 mmol) was dissolved in anhydrous THF (5 mL). The solution was cooled to -78 °C and NEt_3 (444 µL, 3.2 mmol) and (+)-DIP-Cl (0.78 M solution in hexane, 2.11 mL, 1.65 mmol) were added dropwise. The solution was stirred for 0.5 h at -78 °C and then aldehyde **19** (165 mg, 0.75 mmol) in anhydrous THF (3 mL) was added dropwise and the solution stirred for a further 1 h at -78 °C. The reaction mixture was warmed to 0 °C and MeOH (0.86 mL) and MeI (0.48 mL, 7.5 mmol) were added and stirring continued at 0 °C for a further 3 h. The reaction was quenched by the addition of sat. aq. $NaHCO_3$ (5 mL) and then stirred at room temperature for a further 1 h. The two biphasic reaction mixtures were combined, the layers were separated, and the aqueous layer was extracted with EtOAc (3 × 20 mL). The combined organic layers were dried ($MgSO_4$), filtered and the solvent removed *in vacuo*. The crude residue was purified by column chromatography, eluting with 10-20% EtOAc in petroleum ether (40-60 °C) to give a mixture of epimers which were used immediately in the next step. NB polymerisation of this species occurs rapidly when concentrated.

The mixture of epimeric alcohols was dissolved in CH_2Cl_2 (40 mL) and cooled to 0 °C. Dess-Martin periodinane (1.02 g, 2.40 mmol) was added and the solution was stirred at room temperature for 1 h. The reaction quenched by the addition of sat. aq. $Na_2S_2O_3$ (20 mL) and sat. aq. $NaHCO_3$ (4 mL). The aqueous layer was extracted with CH_2Cl_2 (3 × 20), and the combined organic layers were dried ($MgSO_4$), filtered and the solvent removed *in vacuo*. The crude residue was purified by column chromatography, eluting with 20% EtOAc in petroleum ether (40-60 °C) to give **20** as a yellow oil (290 mg, 56%). $[\alpha]_D^{21}$= - 24.0 (*c* 0.5, $CHCl_3$) (Lit. $[\alpha]_D^{21}$= -32.7 (*c* 0.7, $CHCl_3$)).[29] **δ^H (400 MHz, $CDCl_3$)** 7.25 (1H, dd, *J* 15.0, 11.0, 7-H), 6.58 (1H, dd, *J* 15.0, 11.0, 9-H), 6.26-6.14 (3H, m, 6-H, 8-H and 10-H), 5.96 (1H, m, 11-H), 5.26 (1H, d, *J* 3.0, 6′ -*H*H), 5.21 (1H, d, *J* 3.0, 6′-H*H*), 4.12 (3H, s, OCH_3), 3.64 (1H, m, 2-H), 2.80 (1H, m, 4-H), 2.21 (1H, m, 3-*H*H), 1.83 (3H, d, *J* 7.5, 12-H_3), 1.30 (1H, m, 3-H*H*), 1.15 (3H, d, *J* 6.0, 2-CH_3),1.13 (3H, d, *J* 6.0, 4-CH_3). **δ^C (100 MHz, $CDCl_3$)** 203.4 (C-5), 200.7 (C-1), 168.7 (C-5′), 166.4 (C-2′), 149.0 (C-4′), 143.4 (C-7), 142.3 (C-9), 135.5 (C-11), 131.5 (C-10), 128.3 (C-8), 127.4 (C-6), 104.8 (C-1′), 95.9 (C-6′), 62.8 (OCH_3), 42.4 (C-2), 42.2 (C-4), 35.8 (C-3), 18.8 (C-12), 18.0 (2-CH_3), 17.1 (4-CH_3). **HRMS (ESI)** calc. for $[C_{20}H_{24}O_5+H]^+$ 345.1697, found 345.1690. **IR (v_{max}/cm^{-1})** (neat) 2967, 1770, 1683, 1578.

All data are in accordance with the literature.[29]

The above procedure was repeated with (10-^{13}C)**19** (136 mg, 0.61 mmol), giving (10-^{13}C)**20** (56 mg, 27% over 2 steps). **δ^H (400 MHz, $CDCl_3$)** 7.25 (1H, dd, *J* 16.0, 10.5, 7-H), 6.59 (1H, m, 9-H), 6.23 (2H, m, 6-H and 8-H), 6.13 (1H, m, 10-H), 5.97 (1H, m, 11-H), 5.27 (1H, d, *J* 3.0, 6′-*H*H), 5.21 (1H, d, *J* 3.0, 6′-H*H*), 4.12 (3H, s, OCH_3), 3.64 (1H, m, 2-H), 2.81 (1H, m, 4-H), 2.21 (1H, app dt, *J* 14.0, 7.0, 3-*H*H), 1.83 (3H, dd, J 8.0, 6.5, 12-H_3), 1.31 (1H, m, 3-H*H*), 1.15 (3H, d, *J* 6.5, 2-CH_3), 1.13 (3H, d, *J* 6.5, 4-CH_3). **δ^C (100 MHz, $CDCl_3$)** 203.3 (C-5), 200.7 (C-1), 168.7 (C-5′), 166.4 (C-2′), 149.0 (C-4′), 143.4 (d, *J* 8.0, C-7), 142.3 (d, *J* 56.5, C-9), 135.5 (d, *J* 70.5, C-11), 131.6 (C-10 (enhanced)), 128.4 (C-8), 127.3 (d, *J* 16.0, C-6), 104.8 (C-1′),

95.9 (C-6′), 62.8 (OCH_3), 42.4 (C-2), 42.2 (C-4), 35.8 (C-3), 18.8 (C-12), 18.0 (2-CH_3), 17.1 (4-CH_3). **HRMS (ESI)** calc. for $[C_{19}{}^{13}CH_{24}O_5+H]^+$ 346.1730, found 346.1732.

Diels-Alder Adduct 21

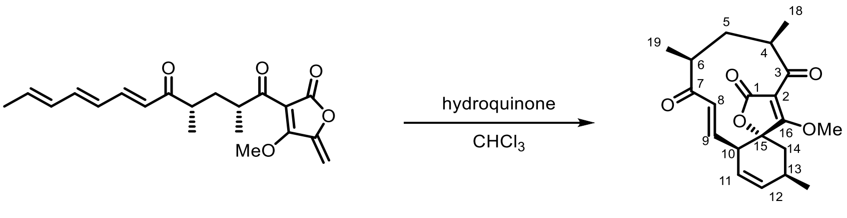

20 **21**

Tetronate **20** (123 mg, 0.092 mmol) and hydroquinone (1 mg) were dissolved in $CHCl_3$ (10 mL), and the solution heated at 75 °C for 48 h. The reaction mixture was cooled to room temperature, the solvent removed *in vacuo* and the crude residue was purified by column chromatography, eluting with 10-20% EtOAc in petroleum ether (40-60 °C) to give Diels-Alder adduct **21** as a colourless oil (88 mg, 72%). $[\boldsymbol{\alpha}]_{\mathbf{D}}^{\mathbf{23}}$= - 206.7 (*c* 0.6, $CHCl_3$) (Lit. $[\alpha]_{D}^{21}$= -202.4 (*c* 0.45, $CHCl_3$)).[29] **δ^H (400 MHz, $CDCl_3$)** 6.47 (1H, dd, *J* 17.0, 7.0, 9-H), 6.24 (1H, d, *J* 17.0, 8-H), 5.85 (1H, app. dt, *J* 10.0, 3.0, 12-H), 5.67 (1H, app. dt, *J* 10.0, 3.0, 11-H), 3.90 (3H, s, OMe), 3.44 (1H, m, 10-H), 3.10 (1H, m, 4-H), 2.94 (1H, app. h, *J* 6.5, 6-H), 2.63 (1H, m, 13-H), 2.40 (1H, dd, *J* 14.5, 8.0, 14-*H*H), 1.85 (1H, overlapping m, 5-*H*H) 1.82 (1H, dd, *J* 14.5, 4.5, 14-H*H*), 1.20 (3H, d, *J* 7.0, 19-H_3), 1.19 (1H, overlapping m, 5-H*H*), 1.18 (3H, d, *J* 7.0, 18-H_3), 1.15 (3H, d, *J* 7.5, 17-H_3). **δ^C (100 MHz, $CDCl_3$)** 204.3 (C-7), 200.6 (C-3), 178.2 (C-16), 169.9 (C-1), 141.5 (C-9), 136.7 (C-12), 131.6 (C-8), 121.7 (C-11), 106.9 (C-2), 86.0 (C-15), 61.7 (OCH_3), 46.62 (C-6), 46.57 (C-4), 44.6 (C-10), 38.9 (C-5), 36.5 (C-14), 29.3 (C-13), 21.1 (C-17), 17.0 (C-19), 16.6 (C-18). **HRMS (ESI)** calc. for $[C_{20}H_{24}O_5+Na]^+$ 362.1938, found 362.1938. **IR (ν_{max}/cm^{-1})** (neat) 2974, 2880, 1751, 1690, 1630, 1454.

All data in accordance with the literature.[29]

The above procedure was repeated with (10-^{13}C)**20** (17 mg, 0.05 mmol), giving (12-^{13}C)**21** (16 mg, 94%). **δ^H (400 MHz, $CDCl_3$)** 6.47 (1H, dd, *J* 17.0, 7.0, 9-H), 6.24 (1H, dd, *J* 17.0, 1.5, 8-

H), 5.85 (1H, app. ddt, *J* 149.5, 10.0, 3.0, 12-H), 5.67 (1H, m, 11-H), 3.90 (3H, s, OMe), 3.44 (1H, m, 10-H), 3.11 (1H, m, 4-H), 2.93 (1H, app. h, *J* 6.5, 6-H), 2.63 (1H, m, 13-H), 2.40 (1H, ddd, *J* 14.5, 8.0, 3.0, 14-*H*H), 1.85 (2H, overlapping m, 5-*H*H and 14-H*H*), 1.20 (3H, d, *J* 6.5, 19-H_3), 1.19 (1H, overlapping m, 5-H*H*), 1.18 (3H, d, *J* 7.0, 18-H_3), 1.15 (3H, dd, *J* 7.5, 17-H_3). **δ^C (100 MHz, $CDCl_3$)** 204.3 (C-7), 200.6 (C-3), 178.2 (C-16), 169.9 (C-1), 141.5 (d, *J* 3.5, C-9), 136.7 (C-12 (enhanced)), 131.6 (C-8), 121.7 (d, *J* 71.0, C-11), 107.0 (C-2), 86.1 (C-15), 61.7 (OCH_3), 46.64 (C-6), 46.59 (C-4), 44.6 (C-10), 38.9 (C-5), 36.6 (d, *J* 2.0, C-14), 29.3 (d, *J* 39.0, C-13), 21.1 (d, *J* 1.5, C-17), 17.0 (C-19), 16.6 (C-18). **HRMS (ESI)** calc. for $[C_{19}{}^{13}CH_{24}O_5+H]^+$ 346.1730, found 346.1724.

Tetronic Acid 3

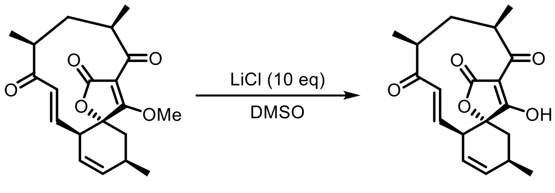

21 3

Under an atmosphere of nitrogen, Diels-Alder adduct **21** (11 mg, 0.032 mmol), was dissolved in anhydrous DMSO (2 mL) and LiCl (14 mg, 0.320 mmol) was added. The resulting suspension was heated at 50 °C for 3 h. The reaction was then allowed to cool to room temperature and diluted with water (5 mL) and 1 M HCl (5 mL). The aqueous layer was extracted with EtOAc (3 × 20 mL), the organic layers combined, washed with sat. aq. LiCl (20 mL), dried ($MgSO_4$), filtered and the solvent removed *in vacuo*. The crude residue was purified by column chromatography, eluting with 70 % EtOAc in petroleum ether (40-60 °C) to give **3** as a white solid (7.4 mg, 70%). $[\alpha]_D^{22}$= - 203.3 (*c* 0.3, MeOH) (Lit. $[\alpha]_D^{22}$= -195.3 (*c* 0.15, MeOH)).[29] **δ^H (400 MHz, CD_3OD)** 6.36 (2H, m, 9-H and 8-H), 5.82 (1H, br d, *J* 10.0, H-12), 5.56 (1H, br d, *J* 10.0, 11-H), 3.86 (1H, m, 4-H), 3.17 (1H, m, 10-H), 2.96 (1H, m, 6-H), 2.49 (1H, m, 13-H), 2.15 (1H, dd, *J* 14.0, 8.0, 14-*H*H), 1.92 (1H, app q, *J* 11.5, 5-*H*H), 1.74 (1H, dd, *J* 14.0, 5.5, 14-H*H*), 1.38 (1H, m, 5-H*H*), 1.18 (3H, d, *J* 7.0, 17-H_3), 1.01 (3H, d, *J* 7.0, 19-H_3)

0.98 (3H, d, *J* 7.5, 18-H_3). **δ^C (100 MHz, CD_3OD)** 208.0 (C-7), 202.0 (C-3), 200.6 (C-16), 175.6 (C-1), 145.5 (C-9), 137.3 (C-12), 134.5 (C-8), 124.3 (C-11), 105.6 (C-15), 87.3 (C-2), 46.9 (C-10), 44.6 (C-6), 43.8 (C-5), 40.2 (C-4), 37.1 (C-14), 30.5 (C-13), 21.5 (C-17), 20.4 (C-19), 19.5 (C-18). **HRMS (ESI)** calc. for $[C_{19}H_{22}O_5+H]^+$ 331.1540, found 331.1526. **IR (ν_{max}/cm^{-1})** (CH_2Cl_2) 3457, 2961, 2929, 1720, 1635, 1543, 1469.

All data in accordance with the literature.[29]

The above procedure was repeated with (12-^{13}C)**21** (16 mg, 0.046 mmol), giving (12-^{13}C)**3** (11 mg, 72%, 3:1 mixture of atropisomers). **δ^H$_{(major)}$ (500 MHz, CD_3OD)** 6.36 (2H, m, 9-H and 8-H), 5.82 (1H, dd, *J* 157.0, 8.5, 12-H), 5.56 (1H, d, *J* 9.5, 11-H), 3.86 (1H, m, 4-H), 3.17 (1H, m, 10-H), 2.96 (1H, m, 6-H), 2.49 (1H, m, 13-H), 2.14 (1H, dd, *J* 14.0, 6.0, 14-*H*H), 1.92 (1H, app. q, *J* 11.0, 5-*H*H), 1.74 (1H, dd, J 12.0, 7.5, 14-H*H*), 1.39 (1H, app. d, *J* 14.0, 5-H*H*), 1.18 (3H, m, 17-H_3), 1.01 (3H, d, *J* 7.0, 19-H_3), 0.98 (3H, d, *J* 7.0, 18-H_3) **δ^C $_{(major)}$(125 MHz, CD_3OD)** 207.9 (C-7), 202.0 (C-3), 200.6 (C-16), 175.6 (C-1), 145.5 (C-9), 137.3 (C-12 (enhanced)), 134.5 (C-8), 124.5 (d, *J* 67.5, C-11), 105.6 (C-15), 87.3 (C-2), 46.9 (C-10), 44.6 (C-6), 43.8 (C-5), 40.1 (C-4), 37.1 (C-14), 30.5 (d, *J* 57.0, C-13), 21.5 (C-17), 20.4 (C-19), 19.5 (C-18). **HRMS (ESI)** calc. for $[C_{18}{}^{13}CH_{22}O_5+Na]^+$ 354.1393, found 354.1399.

5-((*N,N*-dimethylamino)methylene)-4-methoxyfuran-2(5H)-one 29

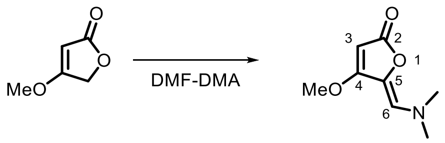

28 **29**

4-Methoxyfuran-2(5H)-one (3.67 g, 32.16 mmol) was dissolved in dimethylformamide dimethyl acetal (30 mL) and heated to 110 °C to allow for the slow and continuous distillation of MeOH. After 8 h the reaction was allowed to cool to room temperature. The solvent was removed *in vacuo* and the crude residue was purified by column chromatography, eluting with 60% EtOAc in petroleum ether (40-60 °C) to give enamine **29** as an orange oil (4.95 g, 91%). **δ^H (400 MHz,**

$CDCl_3$) 6.02 (1H, s, 6-H), 4.86 (1H, s, 3-H), 3.83 (3H, s, OMe), 3.08 (6H, s, $N(CH_3)_2$). **δ^C (100 MHz, $CDCl_3$)** 171.5 (C-2), 170.1 (C-4), 122.9 (C-6), 120.3 (C-5), 80.7 (C-3), 58.5 (OCH_3), 42.6 ($N(CH_3)_2$). **HRMS (ESI)** calc. for $[C_8H_{11}NO_3+H]^+$ 170.0812, found 170.0818. **IR (ν_{max}/cm^{-1})** (neat) 3136, 2935, 1723, 1667, 1563.

All data in accordance with the literature.[30]

5-((*N,N*-dimethylamino)methyl)-4-methoxyfuran-2(5H)-one 22

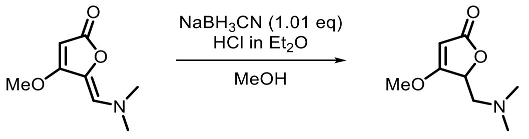

29 **22**

Under an atmosphere of nitrogen, enamine **29** (2.33 g, 13.78 mmol) was dissolved in MeOH and cooled to 0 °C. 2 M HCl in Et_2O (0.5 mL) was added followed by the portion-wise addition of $NaBH_3CN$ (875 mg, 13.92 mmol). The resulting solution was maintained acidic to pH paper (~pH 2) for 15 minutes by the addition of HCl in Et_2O as required (~ 15 mL). The resulting white suspension was warmed to room temperature and stirred for an additional 1 h and then the solvent was removed *in vacuo*. The resulting white solid was dissolved in H_2O (6 mL) and basified to pH 12 with aq. NaOH (5 M). EtOAc (10 mL) was added and the layers were separated. The aqueous layer was extracted with EtOAc (4 × 30 mL), the organic layers combined, dried ($MgSO_4$), filtered and the solvent removed *in vacuo* to give amine **22** as a yellow oil (2.18 g, 93%). **δ^H (400 MHz, $CDCl_3$)** 5.09 (1H, d, *J* 4.5, 3-H), 4.87 (1H, m, 5-H), 3.88 (3H, s, OCH_3), 2.88 (1H, dd, *J* 14.0, 7.0, 6-*H*H), 2.48 (1H, dd *J* 14.0, 7.0, 6-H*H*), 2.33 (6H, d, *J* 7.0, ($N(CH_3)_2$)). **δ^C (100 MHz, $CDCl_3$)** 181.2 (C-2), 172.5 (C-4), 89.4 (C-3), 78.6 (C-5), 60.6 (C-6), 59.6 (OCH_3), 46.2 ($N(CH_3)_2$). **HRMS (ESI)** calc. for $[C_8H_{13}NO_3+Na]^+$ 194.0788, found 194.0781. **IR (ν_{max}/cm^{-1})** (neat) 3477, 2946, 2777, 1746, 1628, 1456.

All data in accordance with the literature.[31]

Epoxide 30

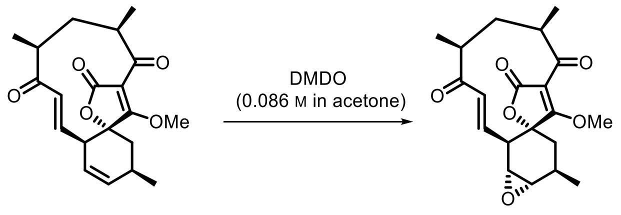

21 **30**

Diels-Alder adduct **21** (52 mg, 0.151 mmol) was cooled to 0 °C and a freshly prepared solution of DMDO in acetone (1.76 mL, 0.151 mmol, 0.086 M in acetone) was added. The reaction was allowed to warm to room temperature over a 4-hour period. The solvent was removed *in vacuo* and the crude residue was purified by column chromatography eluting with 20-35% EtOAc in hexane which gave epoxide **30** as a white solid (33.9 mg, 62% yield). $[\boldsymbol{\alpha}]_{\mathbf{D}}^{\mathbf{21}}$ = -121.0 (*c* 1.15, $CHCl_3$) Lit. $[\alpha]_{D}^{21}$ = -125.0 (*c* 1.10, $CHCl_3$).[32] **δ_H (400 MHz, $CDCl_3$)** 6.43 (1H, dd, *J* 17.0, 7.0, 9-H), 6.31 (1H, dd, *J* 17.0, 1.0, 8-H), 3.98 (3H, s, OMe), 3.21 (1H, dd, *J* 4.0, 3.0, 11-H), 3.14 (1H, m, 4-H), 3.02 (1H, dd, 4.0, 2.5, 12-H), 2.83–2.94 (2H, m, 6-H and 10-H), 2.26 (1H, m, H-13), 2.20 (1H, dd, *J* 14.0, 6.5, 14-*H*H), 1.86 (1H, ddd, *J* 15.5, 6.5, 4.5, 5-*H*H), 1.80 (1H, dd, *J* 14.5, 6.5, 14-H*H*), 1.27-1.25 (1H, overlapping m, 5-H*H*), 1.24 (3H, d, *J* 7.5, CH_3), 1.21 (3H, d, *J* 7.0, CH_3), 1.17 (3H, d, *J* 7.0, CH_3). **δ_C (100 MHz, $CDCl_3$)**: 203.8 (C-7), 200.2 (C-3), 177.3 (C-16), 169.3 (C-1), 138.6 (C-9), 132.5 (C-8), 107.8 (C-2), 85.3 (C-15), 62.1 (O*C*H_3), 57.3 (C-12), 52.1 (C-11), 46.8 (C-6 or C-10), 46.6 (C-6 or C-10), 46.2 (C-4), 39.0 (C-5), 35.3 (C-14), 28.2 (C-13), 19.2 (C-17), 17.2 (C-19) and 16.6 (C-18). **HRMS (ESI)** calc. for $[C_{20}H_{24}O_6+Na]$□ 383.1465, found 383.1460.

Epoxide 4

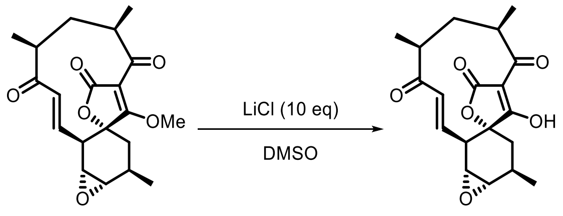

30 4

Epoxide **30** (15 mg, 0.042 mmol) was dissolved in anhydrous DMSO (2 mL) and LiCl (18 mg, 0.42 mmol) was added. The resulting suspension was heated at 50 °C for 3 hours. The reaction mixture was allowed to cool to room temperature and diluted withe with water (5 mL) and 1 M HCl (5 mL). The aqueous layer was extracted with EtOAc (3 × 10 mL), the organic layers combined, washed with sat. aq. LiCl (10 mL), brine (10 mL), dried ($MgSO_4$), filtered and the solvent removed *in vacuo*. The crude residue was dissolved in CH_2Cl_2 (0.5 mL) and AcOH (1-2 drops) and loaded directly to a pipette column, eluting with 80% EtOAc in hexane to give vinylogous epoxide **4** as a white solid (14 mg, 97% yield). **δ_H (400 MHz, $CDCl_3$)** 6.45 (1H, dd, *J* 16.5, 9-H), 6.38 (1H, d, *J* 16.5, 8-H), 3.84 (1H, m, 4-H or 6-H), 3.18 (1H, dd, *J* 4.0, 3.0, 11-H), 3.03 (1H, dd, *J* 4.0, 2.0, 12-H), 2.94 (1H, m, 4-H or 6-H), 2.57 (1H, dd, *J* 8.0, 3.0, 10-H), 2.09 (1H, m, 13-H), 1.96 (1H, m, 5-*H*H), 1.92 (1H, dd, *J* 14.5, 6.0, 14-*H*H), 1.78 (1H, dd, *J* 14.5, 7.0, 14-H*H*), 1.32 (3H, d, *J* 7.5, 17-H_3), 1.30 (1H, overlapping m, 5-H*H*) and 1.01 (6H, d, *J* 6.5, 18-H_3 and 19-H_3). **δ_C (100 MHz, $CDCl_3$)** 207.7 (C-7), 200.3 (C-3), 198.2 (C-16), 177.9 (C-1), 142.7 (C-9), 134.0 (C-8), 103.8 (C-2), 86.4 (C-15), 58.9 (C-12), 54.3 (C-11), 48.2 (C-10) 44.9 (C-4 or C-6), 43.6 (C-5), 40.8 (C-4 or C-6), 35.4 (C-14), 30.0 (C-13), 20.1 (CH_3), 19.4 (CH_3) and 19.3 (CH_3).

All data is in accordance with the literature.[32]

2. Supplementary Tables

Table S1. Summary of X-ray data collection and refinement statistics

	AbyV
**Data collection**	
Space group	*P*1211
Cell dimensions	
a, *b*, *c* (Å)	50.77, 91.83, 82.55
α, β, γ (°)	90, 92.36, 90
Wavelength (Å)	0.91587
Resolution (Å)	61.44 - 2.01 (2.06 - 2.01)
R_{merge} (%)	0.08 (1.31)
$CC_{1/2}$ (%)	1.0 (0.7)
I/sig(I)	14 (1.5)
Completeness (%)	99.8 (100)
Redundancy	6.8 (6.6)
**Refinement**	
Resolution (Å)	61.44 - 2.01
No. reflections	50329/2409
R_{work}/R_{free}	0.19/0.24
No. atoms	5950
Protein	5574
Ligand	225
Water	145
Ions	6
B-factors (Å^2)	
Protein	57.22
Ligand	68.24
Water	48.08
Ions	59.88
Occupancies	
HEME	2
r.m.s deviations	
Bond lengths (Å)	0.0085
Bond angles (°)	1.659
PDB code	7QAN

Values in parentheses are for the highest-resolution shell.

3. Supplementary Figures

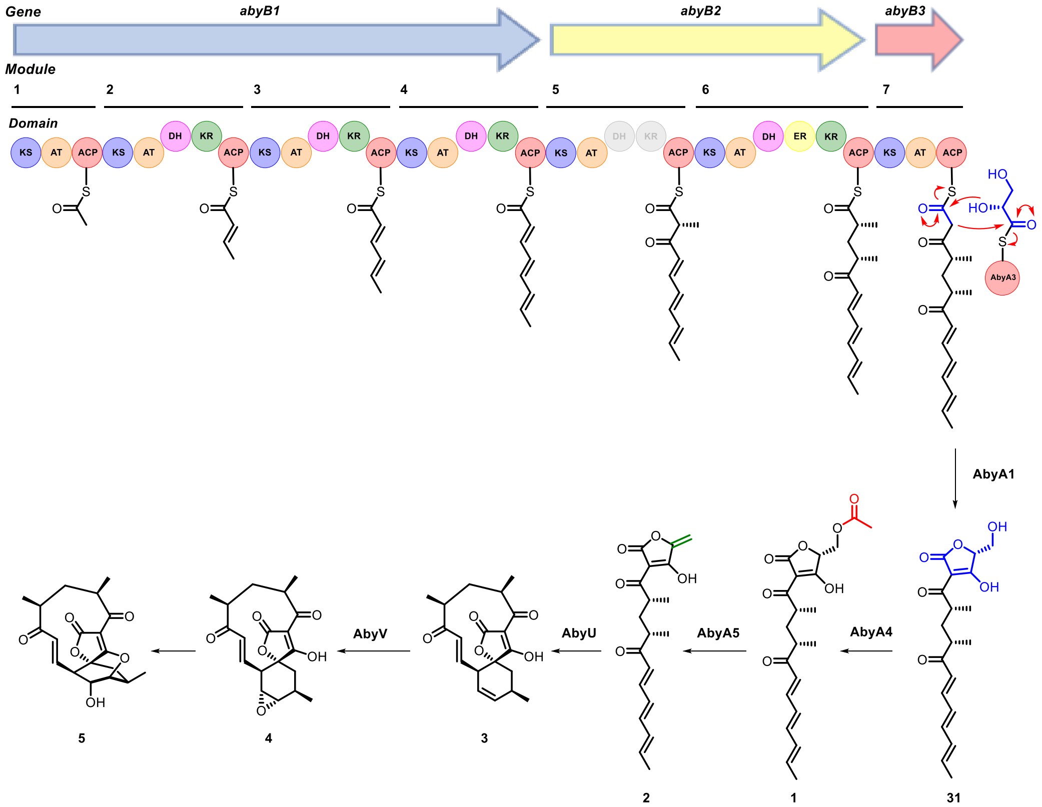

Figure S1. Proposed Abyssomicin C Biosynthetic Pathway. A Type 1 Modular PKS assembles the linear polyketide backbone which is cleaved from the PKS by reaction with an ACP-bound glyceryl unit to give the tetronic acid head group. The tetronic acid **31** then undergoes acetylation and acetate elimination catalysed by AbyA4 and AbyA5 respectively which installs the *exo*-methylene dienophile for the subsequent intramolecular Diels-Alder reaction, catalysed by AbyU. The biosynthesis is then completed via epoxidation of the Diels-Alder adduct by AbyV and subsequent ring opening of the epoxide by the tetronate enol gives abyssomicin C.

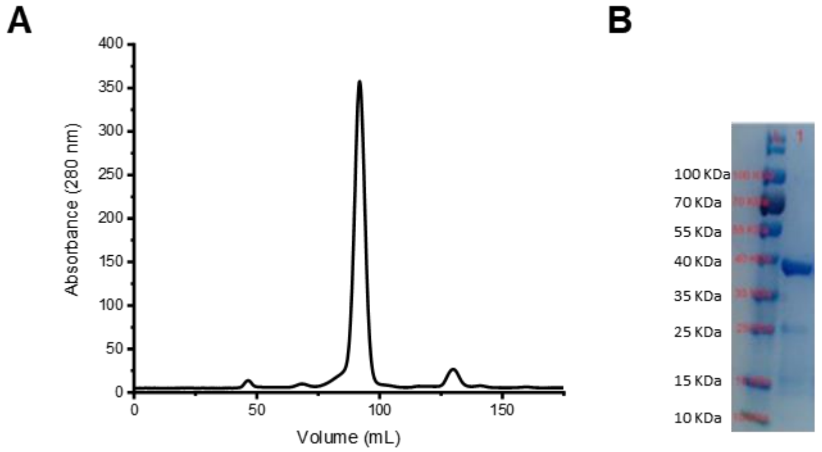

Figure S2. Size exclusion chromatography and SDS PAGE analysis of purified recombinant AbyV. A) Chromatogram showing the elution profile of AbyV from a Superdex 75 10/300 column (GE Healthcare) pre-equilibrated in 50 mM TrisHCl, 500 mM NaCl, pH 7.5. **B**) SDS PAGE analysis of AbyV.

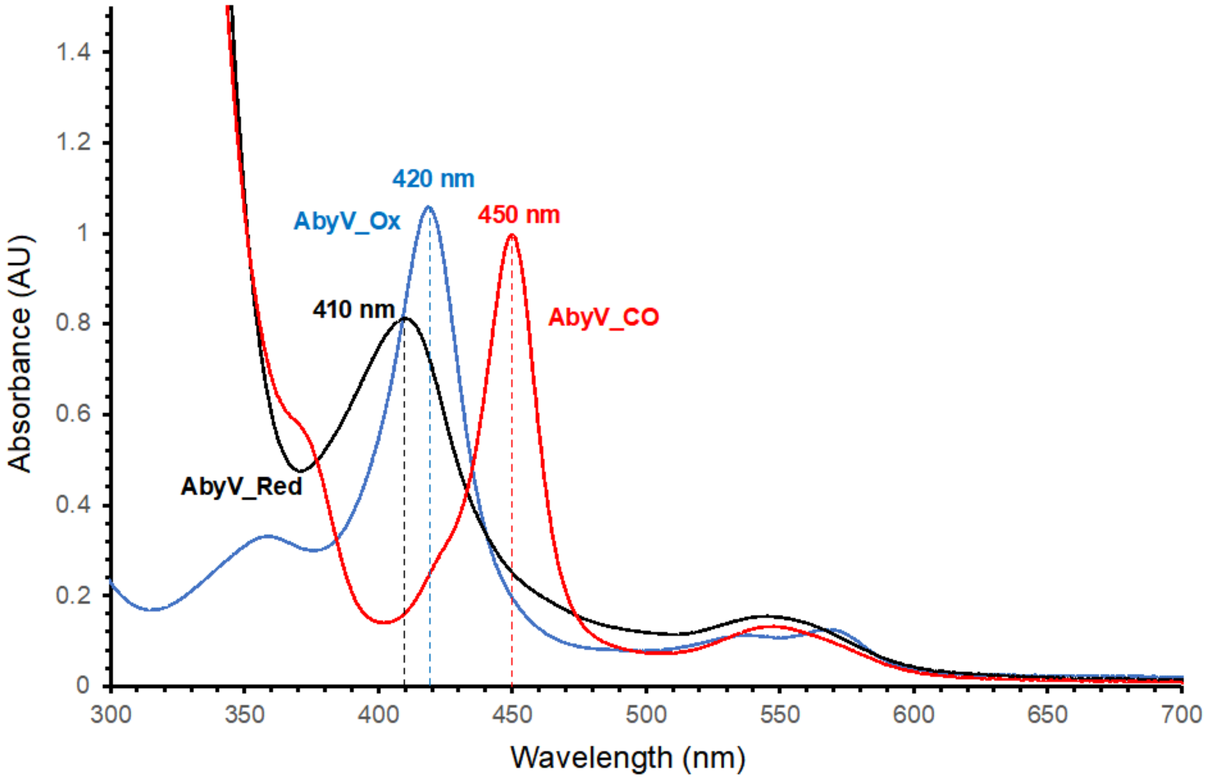

Figure S3. Spectroscopic characterisation of AbyV. UV-visible spectra of oxidized AbyV (blue), sodium dithionite-reduced AbyV (black) and carbon monoxide purged reduced AbyV (red). All data were collected using a Varian Cary 50 spectrophotometer, using a 1 cm path length quartz cuvette in buffer comprising 20 mM Tris-HCl, 150 nM NaCl, pH 7.5, at a temperature of 25°C.

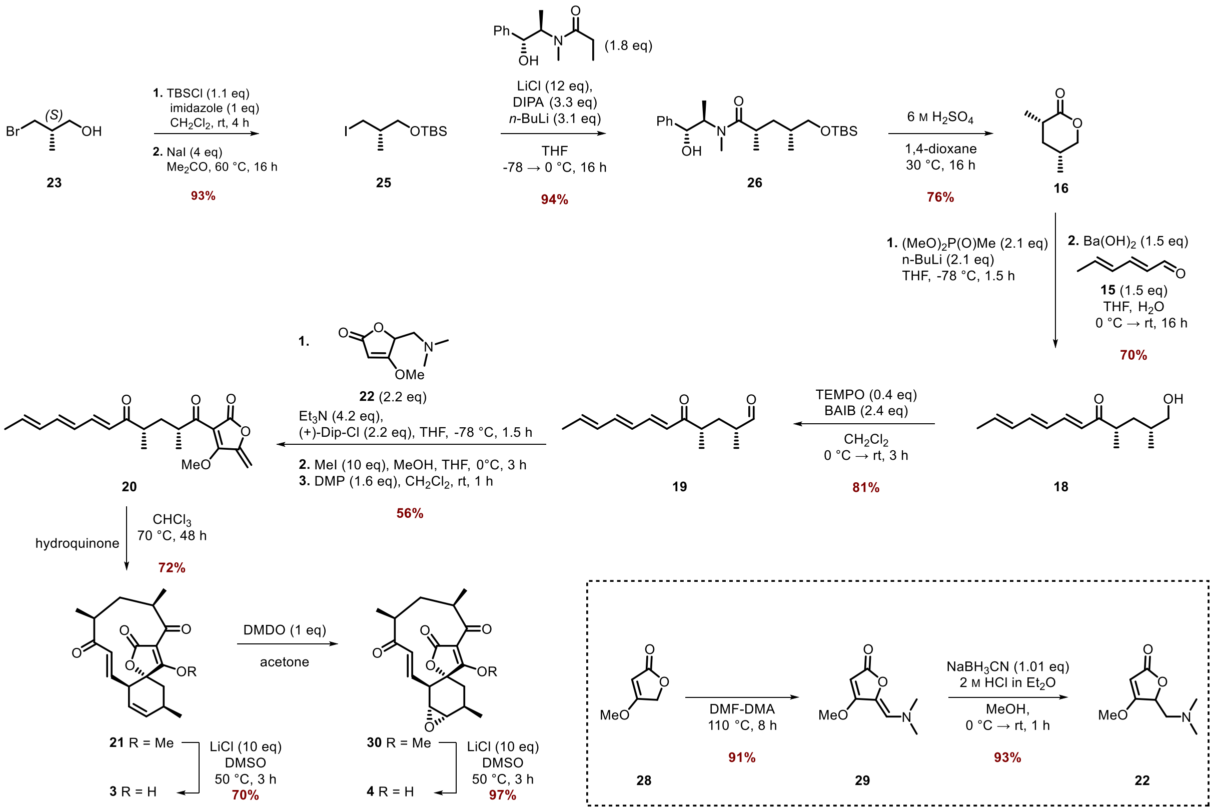

Figure S4. Synthesis of Proposed AbyV Substrate 3 and Epoxide 4.

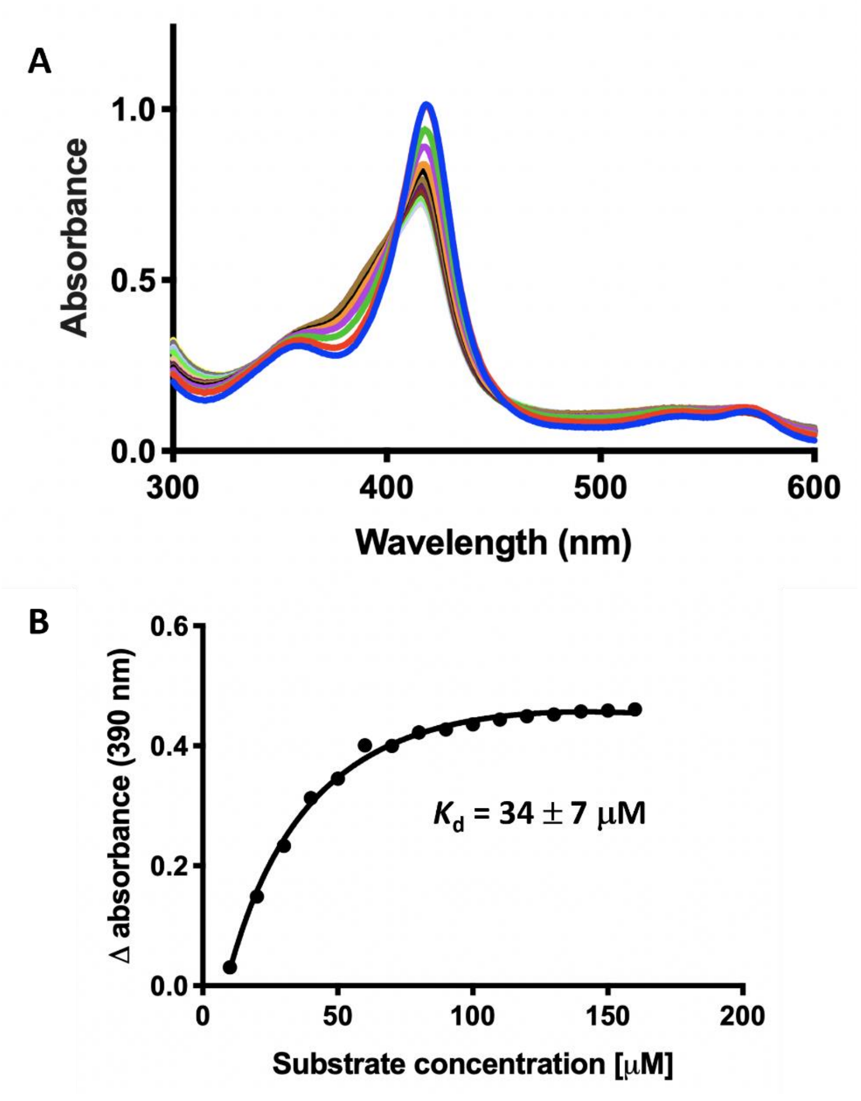

Figure S5. Spectrophotometric Analysis of Compound 3 Binding to AbyV. (A) UV-Visible spectral titration of AbyV with compound **3**. Reported spectra were collected from 0 μM **3** (blue line) to 160 μM **3** (yellow line), in 10 μM increments. (B) Plot of absorbance values at 390 nm vs compound **3** concentration for the data reported in (A). Data have been fitted to a rectangular hyperbolic fitting function to determine the dissociation constant.

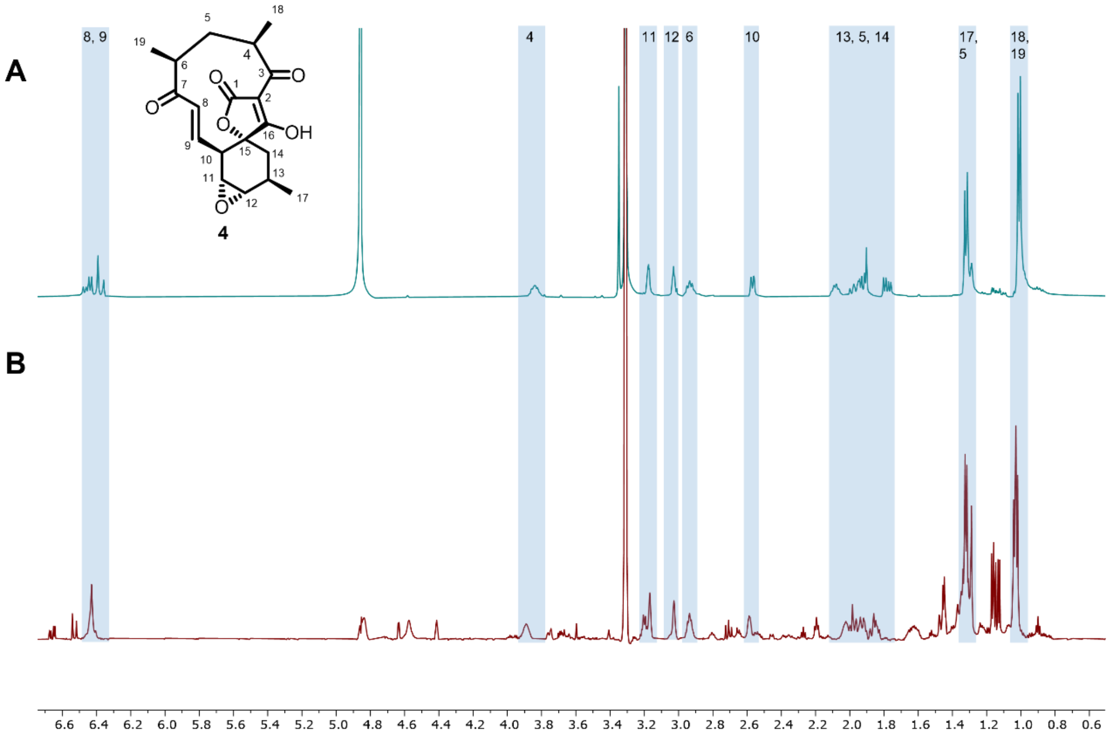

Figure S6. ^{1}H NMR Analysis of Assay Products: A) ^{1}H NMR spectrum of synthetic epoxide **4**. **B)** ^{1}H NMR spectrum of HPLC purified AbyV assay product, with peaks corresponding to epoxide **4** highlighted in blue.

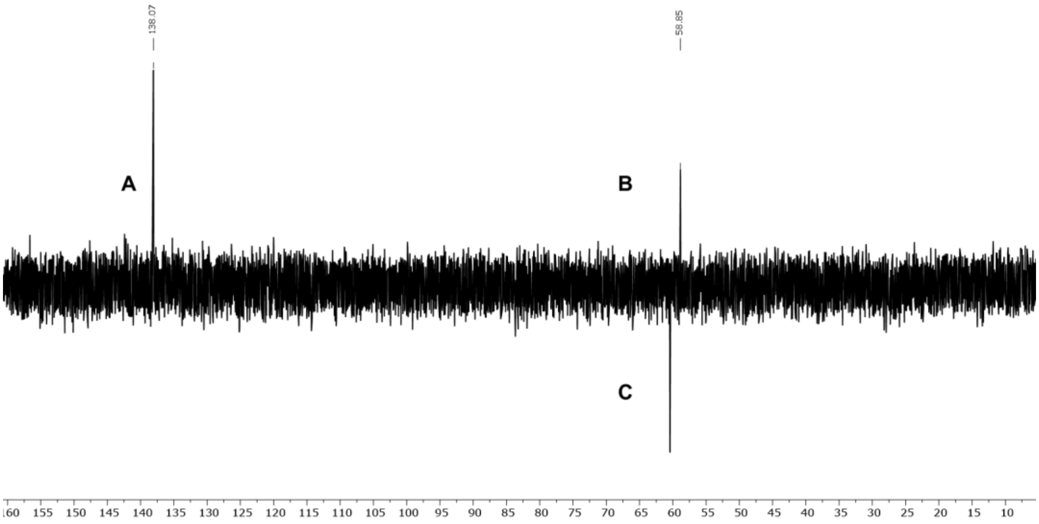

Figure S7. ^{13}C NMR (DEPT) spectrum of AbyV assay mix. A) Signal corresponding to the ^{13}C enriched position of one atropisomer of (12-^{13}C)**3**. **B)** Signal corresponding to the ^{13}C enriched position of epoxide (12-^{13}C)**4**. **C)** Signal corresponding to Tris (-CH_2OH).

```
AbyV      -------MIEIPSAATASPQYP--QRRACPYRPAGGY------ERPVTRVRLYDGRPAWL  45
7DLS      --------------MTEAVAFP--QNRSCPYHPPTAYEPLRE-ERPLSRVTLWNGRQVWF  43
5Y1I      -------------GSHMAPEWPMKRDTGCPFDPPPGVRKLLA-EKPLSRARIWDGSTPWV  46
3ABB      MT-----ETEIRLTGSPAPSFP--QDRTCPYQPPKAYEERRG-ESPLTQVTLFDGRPAWL  52
2Z36      MT-------KNVADELAGLELP--VERGCPFAPPAAYERLRE-RAPINKVRLTSGGQAWW  50
1F25      --------------ASGAPSFP--FSRASGPEPPAEFAKLRA-TNPVSQVKLFDGSLAWL  43
4OQS      -------MRVDSENMNEPVTLPTGRAVGYPFDPPPDLVKL----PPVSPMRFPDGHIGWL  49
3TYW      MTPPESPTASHTPGATPPRDFP--IQRGCPFAAPAEYAALRT-DDPVARVTLPTRREAWV  57
4UBS      ----------------APVAFP--QDRTCPYDPPTAYDPLRE-GRPLSRVSLYDGRSVWV  41
3CV8      MT-----DTATTPQTTDAPAFP--SNRSCPYQLPDGYAQLRDTPGPLHRVTLYDGRQAWV  53
3E5L      ----------MPEPTADAPTVP--KARSCPFLPPDGIADIRA-AAPVTRATFTSGHEAWL  47
                               *                       *:    :      *

AbyV      VTGHETARQVLLDAATFSSDRQH--PAFPALAARFEAARAV----RNFIGMDPPEHTAQR  99
7DLS      VTGHQAARALLGD-QRLSTDSTR--EDFPLPTERSESLR--RQRRGALLGWDDPEHNEQR  98
5Y1I      VTRHADQRALLSD-PRVSHDGLR--DGYPHISADFKFLS---MAQPSFAGQDDPEHGRIR  100
3ABB      ITGHAEGRALLVD-PRLSSDWGH--PDFPVVVRRTEDR---GGLAFPLIGVDDPVHARQR  106
2Z36      VSGHEEARAVLAD-GRFSSDKRK--DGFPLFTLDAATLQQLRSQPPLMLGMDGAEHSAAR  107
1F25      VTKHKDVCFVATS-EKLSKVRTR--QGFPELSASGKQAA---KAKPTFVDMDPPEHMHQR  97
4OQS      VTSHAAARTVMID-PRFSNRPEHKHPVFSVIPRPGG--ATKAPAPGWFINMDAPEHTRYR  106
3TYW      VTRYDDVRELLSD-PRVSADIRR--PGFPALGEGEQEA---GARFRPFIRTDAPEHTRYR  111
4UBS      VTGHAAARALLSD-QRLSSDRTL--PRFPATTERFEAVR---TRRVALLGVDDPEHRTQR  95
3CV8      VTKHEAARKLLGD-PRLSSNRTD--DNFPATSPFFEAVR---ESPQAFIGLDPPEHGTRR  107
3E5L      VTGYEEVRALLRD-SSFSVQVPH-------ALATQDGVVTQKPGRGSLLWQDEPEHTSDR  99
          :: :     :  .   .*                             :   *   *   *

AbyV      RMLISGFTAKRVATLRPAITEIVDSLLDEVVRRG---PGVDLVATFTLPVPSVVICRLLG  156
7DLS      RMLIPSFTLRRAESMRPRIQAIVDRLLDDMIAAG---PSAELVGAFALPVPSMVICELLG  155
5Y1I      RMVTLPFTARRIEAMRPAIQKITDELIDGMLAGP---KPVDLVEALALPVPVRVICEMLG  157
3ABB      RMLIPSFGVKRMNAIRPRLQSLVDRLLDDMLAKG---PGADLVSAFALPVPSVAICELLG  163
2Z36      RPVIGEFTVKRLAALRPRIQDIVDHFIDDMLATD--QRPVDLVQALSLPVPSLVICELLG  165
1F25      SMVEPTFTPEAVKNLQPYIQRTVDDLLEQMKQKGCANGPVDLVKEFALPVPSYIIYTLLG  157
4OQS      RMLISQFTVRRIKELEPRIVQITEDHLDAMAKAG---PPVDLVQAFALPVPSLVICELLG  163
3TYW      RMLLPAFTVRRVRAMRPAVQARVDEILDGMLAAG---GPVDLVSAYANAVSTSVICELLG  168
```

```
4UBS            RMLVPSFTLKRAAALRPRIQETVDGLLDAMEAQG---PPAELVSAFALPLPSMVICALLG   152
3CV8            RMTISEFTVKRIKGMRPEVEEVVHGFLDEMLAAG---PTADLVSQFALPVPSMVICRLLG   164
3E5L            KLLAKEFTVRRMQALRPNIQRIVDEHLDAIEARG---GPVDLVKTFANAVPSMVISDLFG   156
                      *  .    :.* :   ..  :: :         .:**   :  :    *  ::*

AbyV            VPYADHEFFEHQSRRIAAGTSTAAESADAFGQLKRYLLGLIETKG-RGGEDMLDVLVDEQ   215
7DLS            VPYGDHEFFEEQSRRLLRGPA-AEDIEKAFRSLEGYFGELIETKRTDPGEGVIDDLVARQ   214
5Y1I            VPYEDREFLQQNNNAMIYRDTAQGDAQKAAIAQAMYLKELVGTKLGDRGDDILSDL-AVQ   216
3ABB            VPYGDHDFFEECSRNFVGAAT-SAEADAAFGELYTYLHGLVGRKQAEPEDGLLDELIARQ   222
2Z36            VPYTDHDFFQSRTTMMVSRTS-MEDRRRAFAELRAYIDDLITRKESEPGDDLFSRQIARQ   224
1F25            VPFNDLEYLTQQNAIRTNGSSTAREASAANQELLDYLAILVEQRLVEPKDDIISKLCTEQ   217
4OQS            VSYADHAFFQEQTTIMASVDKTQDEVTTALGKLTRYIAELVATKRLSPKDDLLGSLITD-   222
3TYW            IPRHDLEFFRDVTRISGSRNSTAEQVSEALGGLFGLLGGLVAERREEPRDDLISKLVTDH   228
4UBS            VPYADHDFFESQSRRLLRGPG-IAEVQDARAQLDDYLYALIDRKRKEPGDGLLDDLIQEQ   211
3CV8            VPYADHEFFQDASKRLVQSTD-AQSALTARNDLAGYLDGLITQFQTEPGAGLVGALVADQ   223
3E5L            VPVERRAEFQDIAEAMMRVDQDAAATEAAGMRLGGLLYQLVQERRANPGDDLISALITTE   216
                :       :                   *       :  *:         .:..

AbyV            VATGTVTTPDLVDLALLLLVAGHETTASTLALGVALLLEQDG--GAVAA--DPTRVGAVV   271
7DLS            REEGRPDDDELVQFATVLLVAGHETTANMISLATYTLLEHPARLAELRA--DPGLVPAAV   272
5Y1I            VEAGEITQDDAAGIGMMLLGAGHETTANMIALGTLALLENPEQLAEVRDSDDPKVIVNTV   276
3ABB            LEEGDLDHDEVVMIALVLLVAGHETTVNAIALGALTLIQHPEQIDVLLR--DPGAVSGVV   280
2Z36            RQEGTLDHAGLVSLAFLLLTAGHETTANMISLGVVGLLSHPEQLTVVKA--NPGRTPMAV   282
1F25            VKPGNIDKSDAVQIAFLLLVAGNANMVNMIALGVATLAQHPDQLAQLKA--NPSLAPQFV   275
4OQS            ---TDLTDEELTNIALLLLVAGHETTANMLGLGTFALLQHPEQIAALDS-------PDAV   272
3TYW            LVPGNVTTEQLLSTLGITINAGRETTTSMIALSTLLLLDRPELPAELRK--DPDLMPAAV   286
4UBS            LNRGTVDRAELVSLATLLLIAGHETTANMISLGTFTLLRHPEQLAELRA--EPGLMPAAV   269
3CV8            LANGEIDREELISTAMLLLIAGHETTASMTSLSVITLLDHPEQYAALRA--DRSLVPGAV   281
3E5L            DPDGVVDDMFLMNAAGTLLIAAHDTTACMIGLGTALLLDSPDQLALLRE--DPSLVGNAV   274
                                  : *.. . .   .*..  *         :            *

AbyV            EEILRHTAVAD-GV-ARFATRDTEVAGVRIAAGDAVVVALSAANRDPGPFP-DPDRFDPR   328
7DLS            EELLRFLSIAD-GL-VRVAREDVPVGDQVIRAGEGVVFPTSLINRDDSVYE-HPDTLDWS   329
5Y1I            EELLRYLTIAQ-DTVRRIAAEDIEIGGVVIKAGEGIVFPLNAANWDPDLYPEAPDRLDIH   335
3ABB            EELLRFTSVSD-HI-VRMAKEDIEVGGATIKAGDAVLVSITLMNRDAKAYE-NPDIFDAR   337
2Z36            EELLRYFTIAD-GVTSRLATEDVEIGGVSIKAGEGVIVSMLSANWDPAVFK-DPAVLDVE   340
1F25            EELCRYHTASALAI-KRTAKEDVMIGDKLVRANEGIIASNQSANRDEEVFE-NPDEFNMN   333
4OQS            EELLRYLSIVHLGTPNRAALEDVELEGQMIRKGDTVAIGLPAVNRDPKVFD-EPDILQLD   331
3TYW            DELLRVLSVAD-SIPLRVAAEDIELSGRTVPADDGVIALLAGANHDPEQFD-DPERVDFH   344
4UBS            EELLRFLSIAD-GL-LRVATEDIEVAGTTIRADEGVVFATSVINRDAAGFA-EPDALDWH   326
3CV8            EELLRYLAIAD-IAGGRVATADIEVEGQLIRAGEGVIVVNSIANRDGTVYE-DPDALDIH   339
3E5L            EELLRYLTIGQ-FGGERVATRDVELGGVRIAKGEQVVAHVLAADFDPAFVE-EPERFDIT   332
                :*: *  :        * *  *  : .  :  .: :       : *       *  .:

AbyV            R--GGRQHVTFGHGPHQCIGANLARAELEIALSRLFTRLPTLALAVPVEELGGKEAGGVQ   386
7DLS            R--SARHHVAFGFGIHQCLGQNLARIELEIALGTLLRRLPGLRLAAPADRIPFKPGDTIQ   387
5Y1I            RA-NARRHLAFGYGVHQCLGATLARVELQIVYSTLLRRIPTLALAGTLDEIPFKHDQIAH   394
3ABB            R--NARHHVGFGHGIHQCLGQNLARAELEIALGGLFARIPGLRLAVPLDEVPIKAGHDAQ   395
2Z36            R--GARHHLAFGFGPHQCLGQNLARMELQIVFDTLFRRIPSLRLAVPMEDVPFKGDSVIY   398
1F25            RKWPPQDPLGFGFGDHRCIAEHLAKAELTTVFSTLYQKFPDLKVAVPLGKINYTPLNRDV   393
4OQS            RV-DARKHAAFGGGIHQCLGQQLARVEMRIGFTRLFARFPSLRLAVPAEEIKLREKSAAY   390
3TYW            R--TDNHHVAFGYGVHQCVGQHLARLELEVALETLLRRVPTLRLAGERDQVVVKHDSATF   402
4UBS            R--SARHHVAFGFGIHQCLGQNLARAEMEIALGTLFERLPGLRLAAPADEIPFKPGDTIQ   384
3CV8            R--SARHHLAFGFGVHQCLGQNLARLELEVILNALMDRVPTLRLAVPVEQLVLRPGTTIQ   397
3E5L            R--RPAPHLAFGFGAHQCIGQQLARIELQIVFETLFRRLPGLRLAKPVEELRFRHDMVFY   390
                *         ** * *:*:.  **: *:      *  :.* * :*     :

AbyV            GVQRLPVTW----------------------------      395
7DLS            GMLELPVTW----------------------------      396
5Y1I            GVYELPVTW----------------------------      403
3ABB            GPIELPVVW----------------------------      404
2Z36            GVHELPVTW----------------------------      407
1F25            GIVDLPVIF----------------------------      402
4OQS            GVWALPVAWDARPSFLVQSGYVEQKLISEEDLNSAVD      427
3TYW            GLEELMVTW----------------------------      411
4UBS            GMLELPVTW----------------------------      393
3CV8            GVNELPVTW----------------------------      406
3E5L            GVHELPVTW----------------------------      399
                *   * * :
```

Figure S8. Sequence Alignment of AbyV with its Closest Structural Homologues. The ten most structurally related unique AbyV homologues were identified using DALI[33] and are

listed by PDB code in order of z-score (highest to lowest). The sequence alignment was performed using Clustal Omega.[34] Residues conserved in all proteins are highlighted in yellow boxes.

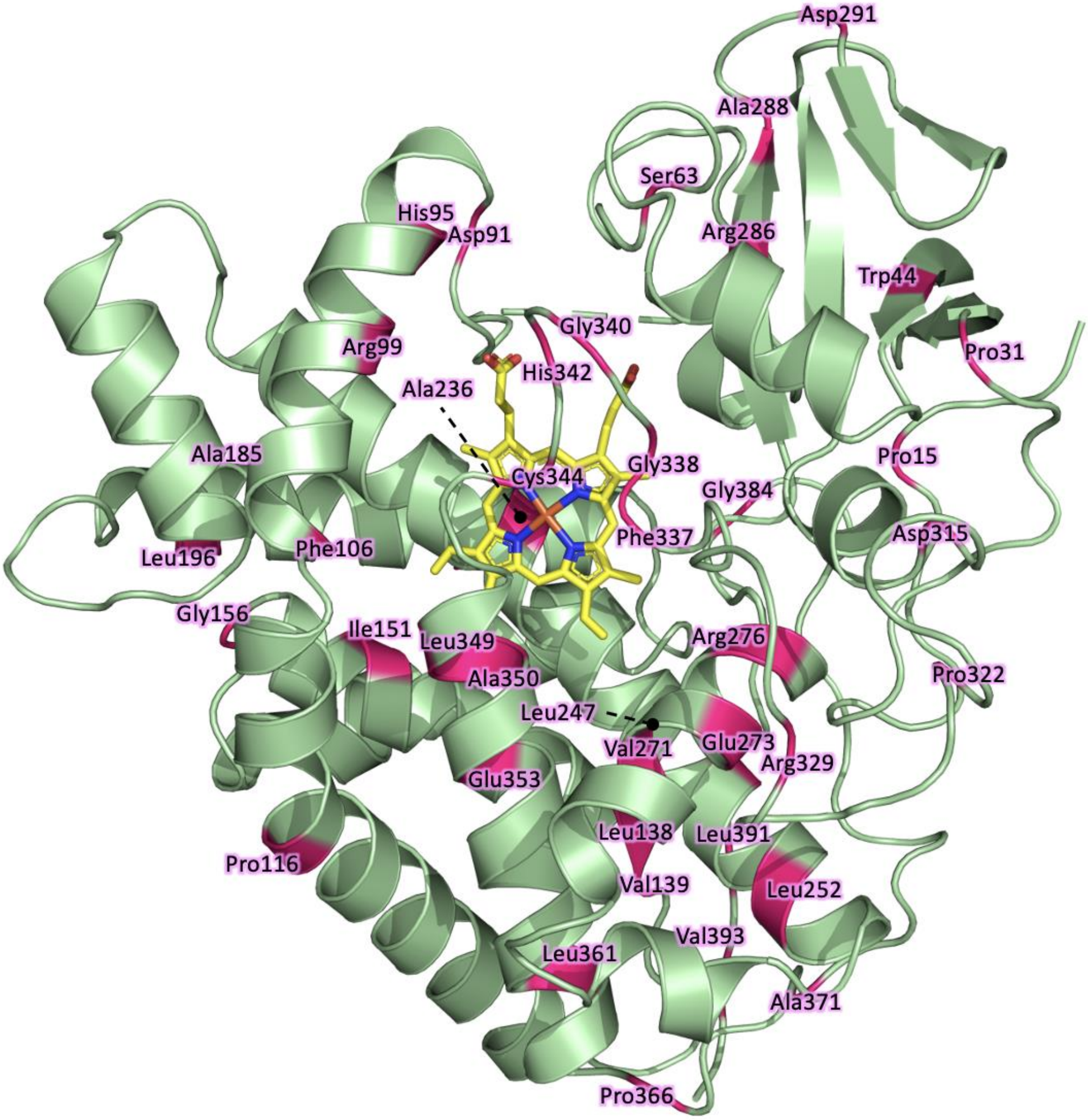

Figure S9. Conserved amino acids identified in Figure S7 mapped onto the crystal structure of AbyV. AbyV is shown in cartoon representation with conserved amino acids highlighted in magenta.

4. Supplementary References

[1] G. Winter, C. M. C. Lobley, S. M. Prince, *Acta Crystallogr. Sect. D Biol. Crystallogr.* **2013**, *69*, 1260–1273.

[2] A. Vagin, A. Teplyakov, *Acta Crystallogr. Sect. D Biol. Crystallogr.* **2010**, *66*, 22–25.

[3] L. H. Xu, S. Fushinobu, S. Takamatsu, T. Wakagi, H. Ikeda, H. Shoun, *J. Biol. Chem.* **2010**, *285*, 16844–16853.

[4] P. Emsley, K. Cowtan, *Acta Crystallogr. Sect. D Biol. Crystallogr.* **2004**, *60*, 2126–2132.

[5] G. N. Murshudov, P. Skubák, A. A. Lebedev, N. S. Pannu, R. A. Steiner, R. A. Nicholls, M. D. Winn, F. Long, A. A. Vagin, *Acta Crystallogr. Sect. D Biol. Crystallogr.* **2011**, *67*, 355–367.

[6] W. DeLano, *CCP4 Newsl. Protein Crystallogr* **2002**.

[7] M. J. Byrne, N. R. Lees, L.-C. Han, M. W. van der Kamp, A. J. Mulholland, J. E. M. Stach, C. L. Willis, P. R. Race, *J. Am. Chem. Soc.* **2016**, *138*, 6095–6098.

[8] O. Trott, A. J. Olson, *J. Comput. Chem.* **2010**, *31*, 455–461.

[9] B. John, A. Sali, *Nucleic Acids Res.* **2003**, *31*, 3982–3992.

[10] A. Fiser, R. Kinh, G. Do, A. S. ˇ Ali, *Protein Sci.* **2000**, *9*, 1753–1773.

[11] “GitHub - marcvanderkamp/enlighten: Protocols and tools to run (automated) atomistic simulations of enzyme-ligand systems,” can be found under https://github.com/marcvanderkamp/enlighten, **n.d.**

[12] R. M. B. D. A. Case, D. S. Cerutti, L. X. and P. A. K. T. E. C. III, T. A. Darden, R. E. Duke, T. J. Giese, H. Gohlke, A. W. Goetz, N. Homeyer, S. Izadi, P. Janowski, J. Kaus, A. Kovalenko, T. S. Lee, S. LeGrand, P. Li, C. Lin, T. Lunchko, R. Luo, B. Madej, D. Mermelstein, K. M. Merz, G. Monard, H. Nguyen, H., *Amber 16, Univ. California, San*

Fr. **2016**.

[13] C. R. Søndergaard, M. H. M. Olsson, M. Rostkowski, J. H. Jensen, *J. Chem. Theory Comput.* **2011**, *7*, 2284–2295.

[14] M. H. M. Olsson, C. R. SØndergaard, M. Rostkowski, J. H. Jensen, *J. Chem. Theory Comput.* **2011**, *7*, 525–537.

[15] J. A. Maier, C. Martinez, K. Kasavajhala, L. Wickstrom, K. E. Hauser, C. Simmerling, *J. Chem. Theory Comput.* **2015**, *11*, 3696–3713.

[16] J. Wang, R. M. Wolf, J. W. Caldwell, P. A. Kollman, D. A. Case, *J. Comput. Chem.* **2004**, *25*, 1157–1174.

[17] K. Shahrokh, A. Orendt, G. S. Yost, T. E. Cheatham, *J. Comput. Chem.* **2012**, *33*, 119–133.

[18] R. Lonsdale, J. N. Harvey, A. J. Mulholland, *J. Phys. Chem. B* **2010**, *114*, 1156–1162.

[19] W. C. Still, M. Kahn, A. Mitra, *J. Org. Chem.* **1978**, *43*, 2923–2925.

[20] T. K. Jones, R. A. Reamer, R. Desmond, S. G. Mills, *J. Am. Chem. Soc.* **1990**, *112*, 2998–3017.

[21] P. Kraft, W. Tochtermann, *Liebigs Ann. der Chemie* **1994**, *1994*, 1161–1164.

[22] A. D. Wadsworth, D. P. Furkert, J. Sperry, M. A. Brimble, *Org. Lett.* **2012**, *14*, 5374–5377.

[23] M. Jiménez, W. Zhu, A. Vogt, B. W. Day, D. P. Curran, *Beilstein J. Org. Chem.* **2011**, *7*, 1372–1378.

[24] M. B. Andrus, W. Li, R. F. Keyes, *J. Org. Chem.* **1997**, *62*, 5542–5549.

[25] J. Alemán, V. Del Solar, C. Navarro-Ranninger, *Chem. Commun.* **2010**, *46*, 454–456.

[26] Y.-Z. Jin, D.-X. Fu, N. Ma, Z.-C. Li, Q.-H. Liu, L. Xiao, R.-H. Zhang, *Molecules* **2011**,

16, 9368–9385.

[27] J. Yu, Y. Zhou, Z. Lin, R. Tong, *Org. Lett.* **2016**, *18*, 4734–4737.

[28] C. W. Zapf, B. A. Harrison, C. Drahl, E. J. Sorensen, *Angew. Chemie Int. Ed.* **2005**, *44*, 6533–6537.

[29] B. B. Snider, Y. Zou, *Org. Lett.* **2005**, *7*, 4939–4941.

[30] S. W. Haynes, P. K. Sydor, A. E. Stanley, L. Song, G. L. Challis, *Chem. Commun.* **2008**, 1865–1867.

[31] K. Takeda, S. G. Yano, M. A. Sato, E. Yoshii, *J. Org. Chem.* **1987**, *52*, 4135–4137.

[32] C. W. Zapf, B. A. Harrison, C. Drahl, E. J. Sorensen, *Angew. Chemie - Int. Ed.* **2005**, *44*, 6533–6537.

[33] L. Holm, *Nucleic Acids Res.* **2022**, *50*, W210–W215.

[34] F. Sievers, A. Wilm, D. Dineen, T. J. Gibson, K. Karplus, W. Li, R. Lopez, H. McWilliam, M. Remmert, J. Söding, J. D. Thompson, D. G. Higgins, *Mol. Syst. Biol.* **2011**, *7*, 539.

6. ^{1}H and ^{13}C NMR Spectra

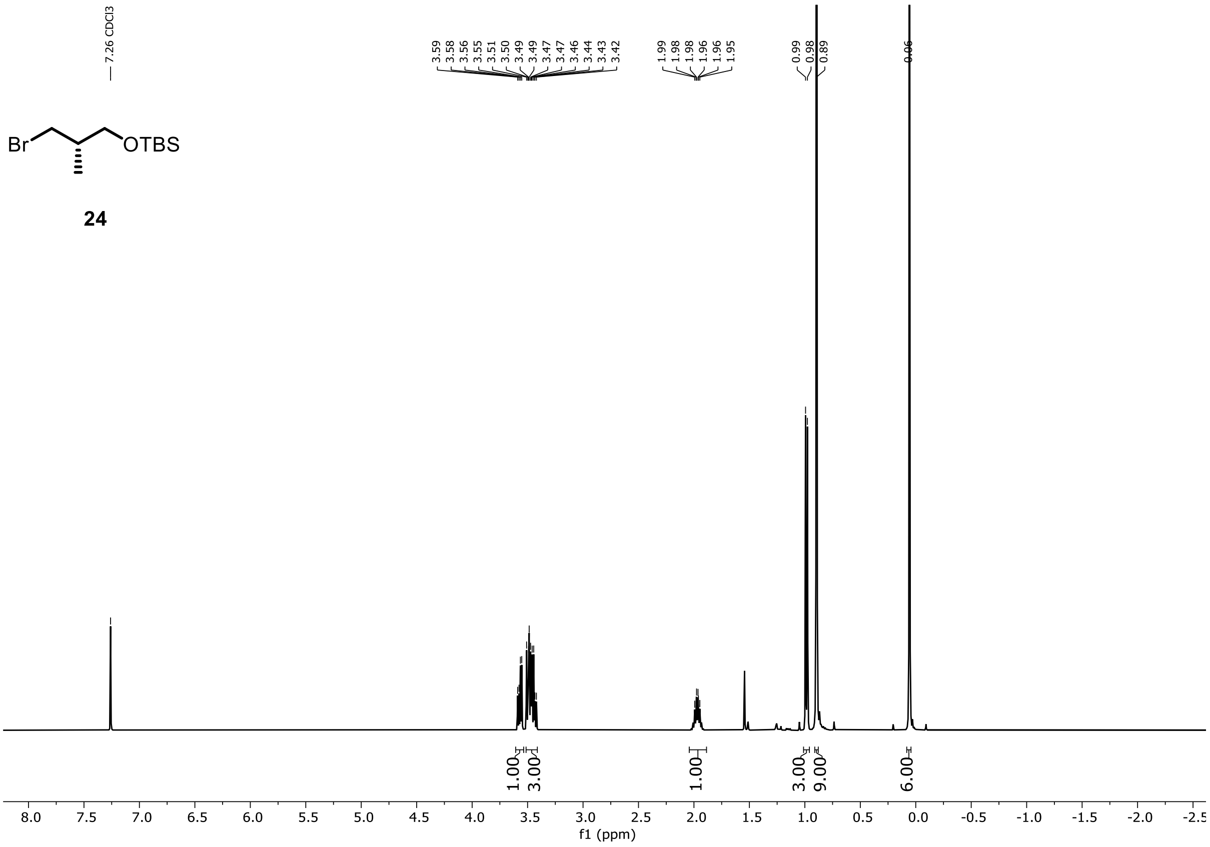

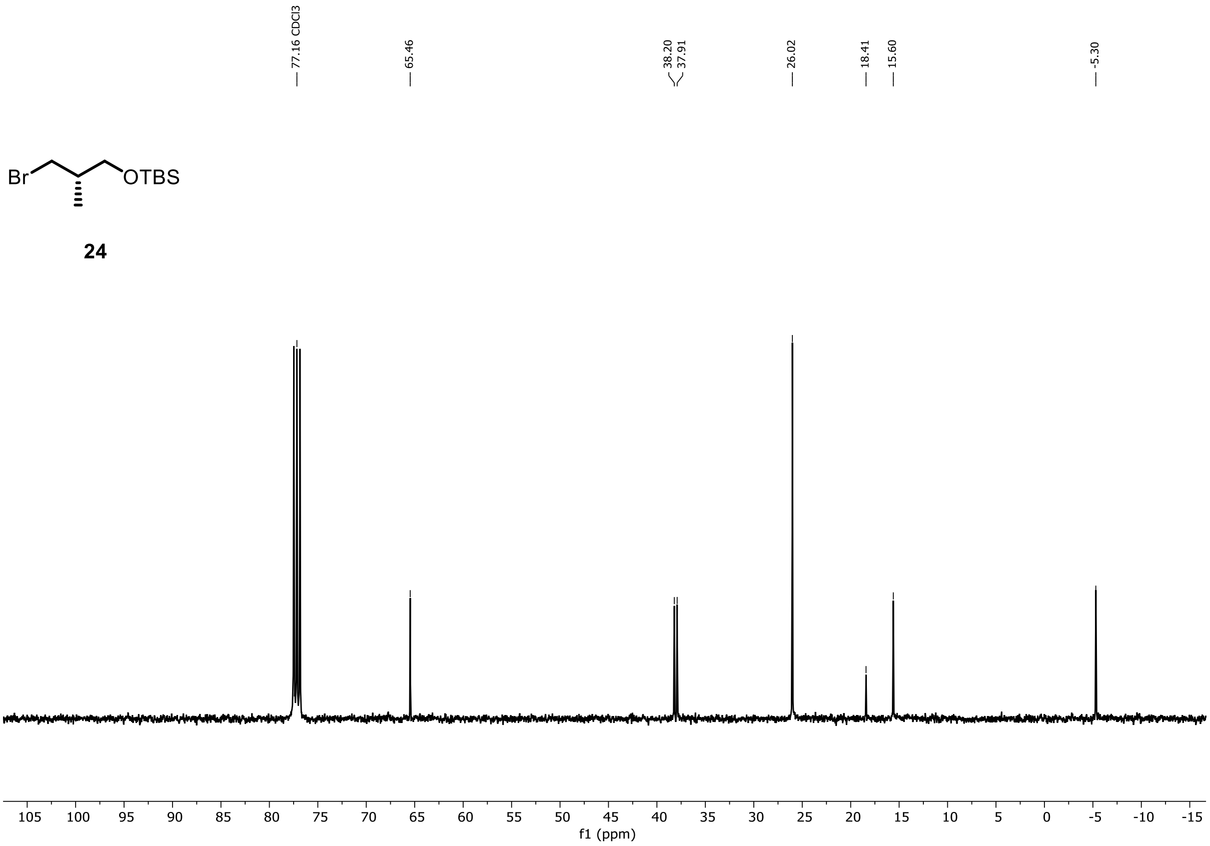

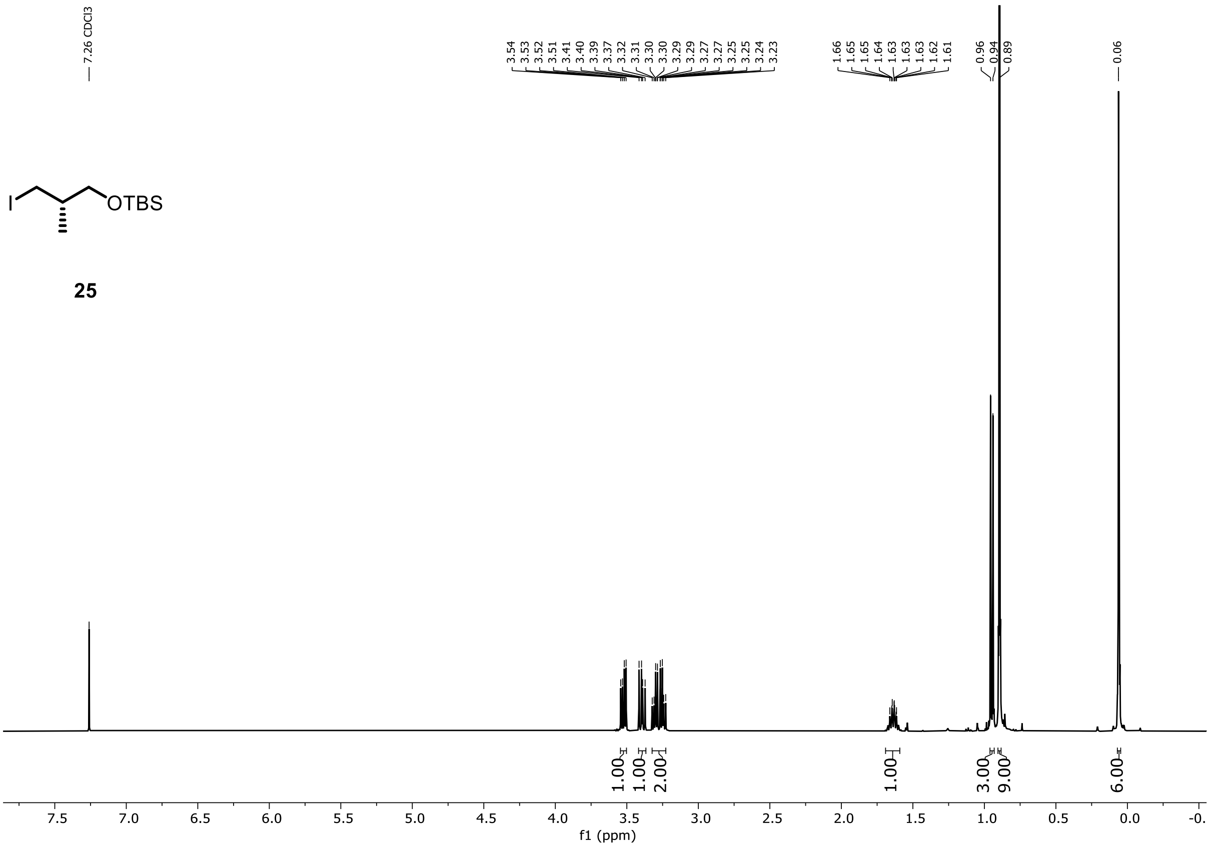
7.26 CDCl3
3.54
3.53
3.52
3.51
3.41
3.40
3.39
3.37
3.32
3.31
3.30
3.30
3.29
3.29
3.27
3.27
3.25
3.25
3.24
3.23
1.66
1.65
1.65
1.64
1.63
1.63
1.63
1.63
1.62
1.61
0.96
0.94
0.89
0.06
I
OTBS
1.00
1.00
2.00
1.00
3.00
9.00
6.00
7.5 7.0 6.5 6.0 5.5 5.0 4.5 4.0 3.5 3.0 2.5 2.0 1.5 1.0 0.5 0.0 -0.
f1 (ppm)

25

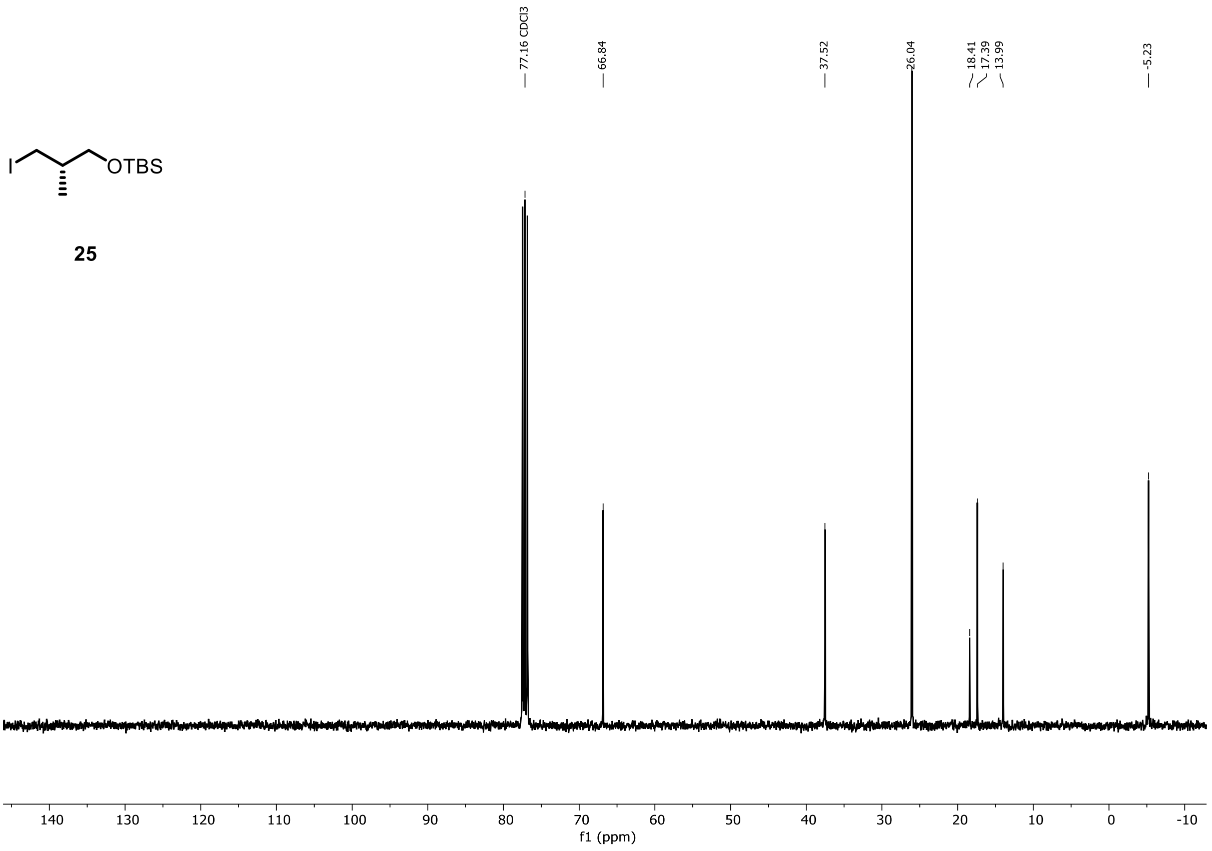
77.16 CDCl3
66.84
37.52
26.04
18.41
17.39
13.99
-5.23
I
OTBS
140 130 120 110 100 90 80 70 60 50 40 30 20 10 0 -10
f1 (ppm)

25

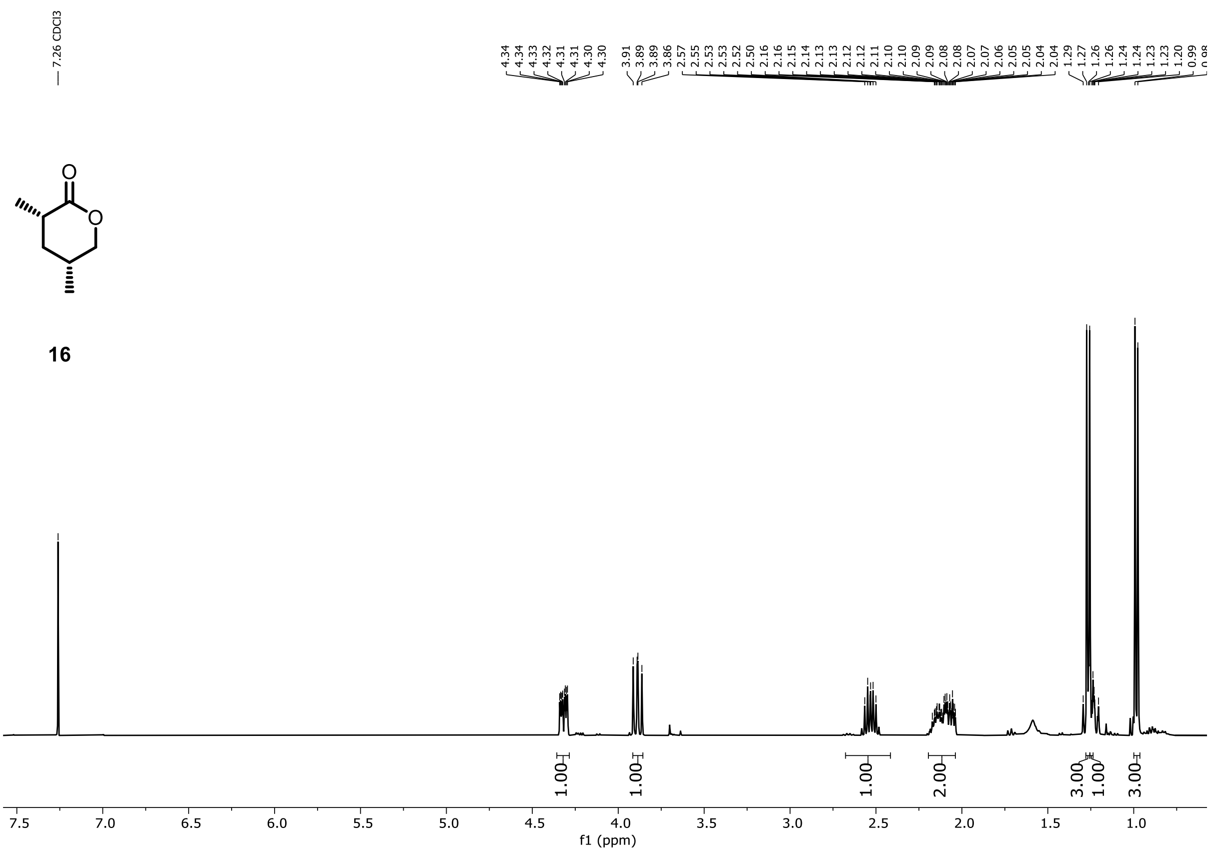

16

7.26 CDCl3
4.34
4.34
4.33
4.32
4.31
4.31
4.30
4.30
3.91
3.89
3.89
3.86
2.57
2.55
2.53
2.53
2.52
2.50
2.16
2.16
2.15
2.14
2.13
2.13
2.12
2.12
2.11
2.10
2.10
2.09
2.09
2.08
2.08
2.07
2.07
2.06
2.05
2.05
2.04
2.04
1.29
1.27
1.26
1.26
1.24
1.24
1.23
1.23
1.20
0.99
1.00
1.00
1.00
2.00
3.00
1.00
3.00
7.5
7.0
6.5
6.0
5.5
5.0
4.5
4.0
3.5
3.0
2.5
2.0
1.5
1.0
f1 (ppm)

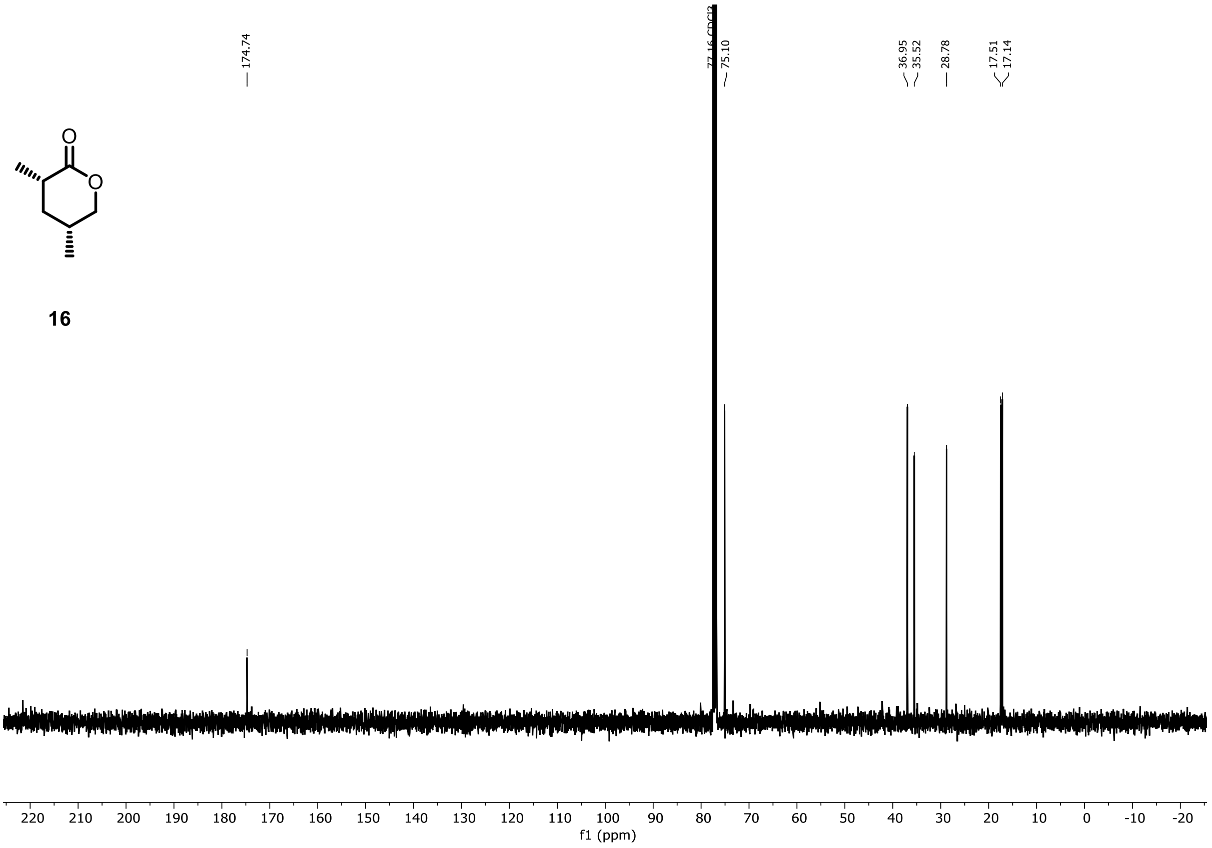

16

174.74
77.16 CDCl3
75.10
36.95
35.52
28.78
17.51
17.14
220
210
200
190
180
170
160
150
140
130
120
110
100
90
80
70
60
50
40
30
20
10
0
-10
-20
f1 (ppm)

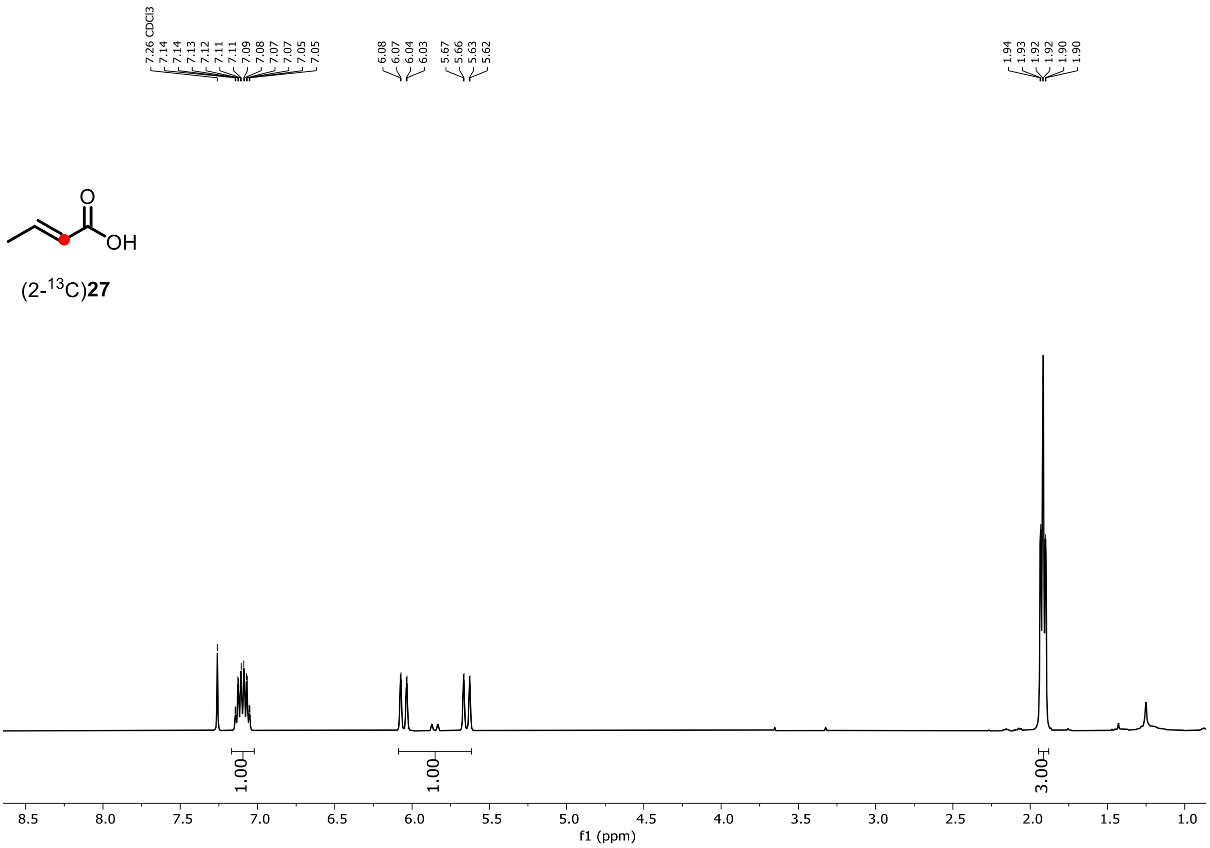
7.26 CDCl3
7.14
7.14
7.13
7.12
7.11
7.11
7.09
7.08
7.07
7.07
7.05
7.05
6.08
6.07
6.04
6.03
5.67
5.66
5.63
5.62
1.94
1.93
1.92
1.92
1.90
1.90
O
OH
(2-13C)27
1.00
1.00
3.00
8.5
8.0
7.5
7.0
6.5
6.0
5.5
5.0
4.5
4.0
3.5
3.0
2.5
2.0
1.5
1.0
f1 (ppm)

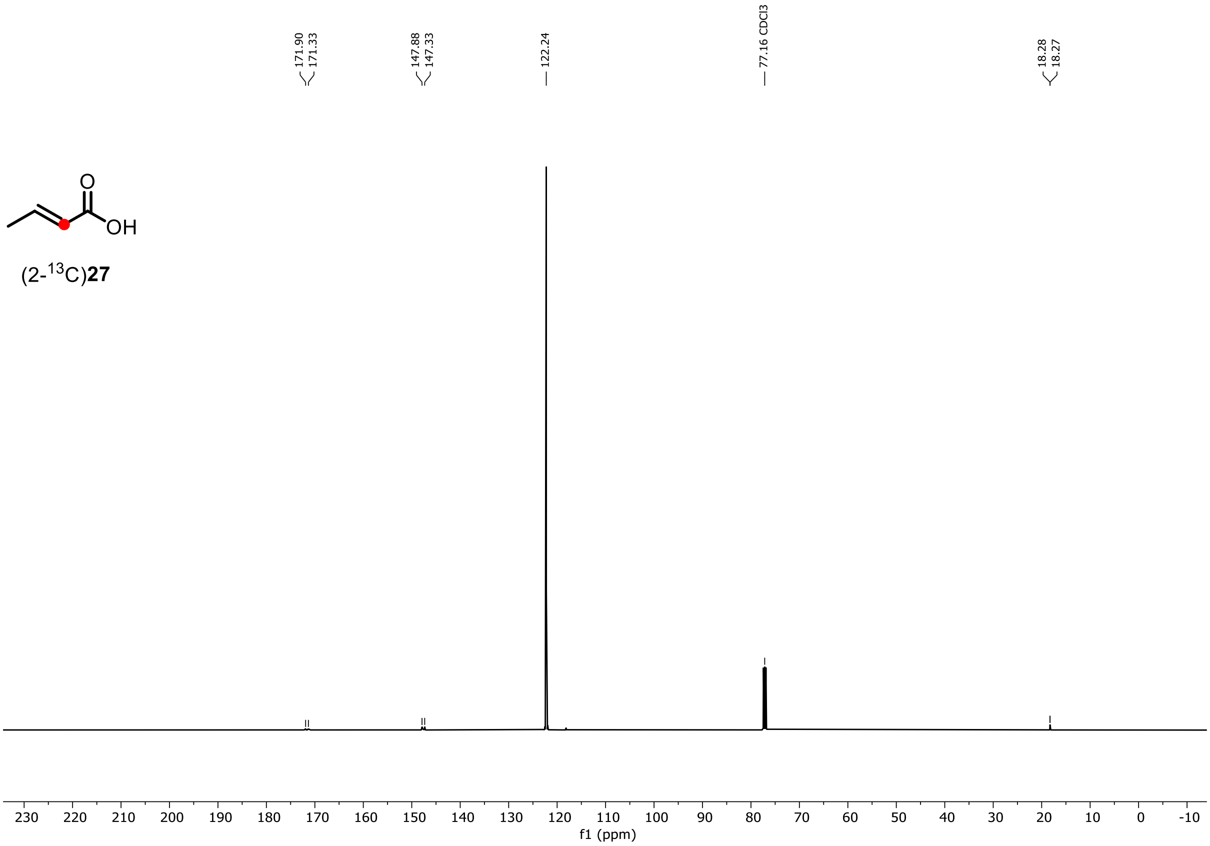
171.90
171.33
147.88
147.33
122.24
77.16 CDCl3
18.28
18.27
O
OH
(2-13C)27
230
220
210
200
190
180
170
160
150
140
130
120
110
100
90
80
70
60
50
40
30
20
10
0
-10
f1 (ppm)

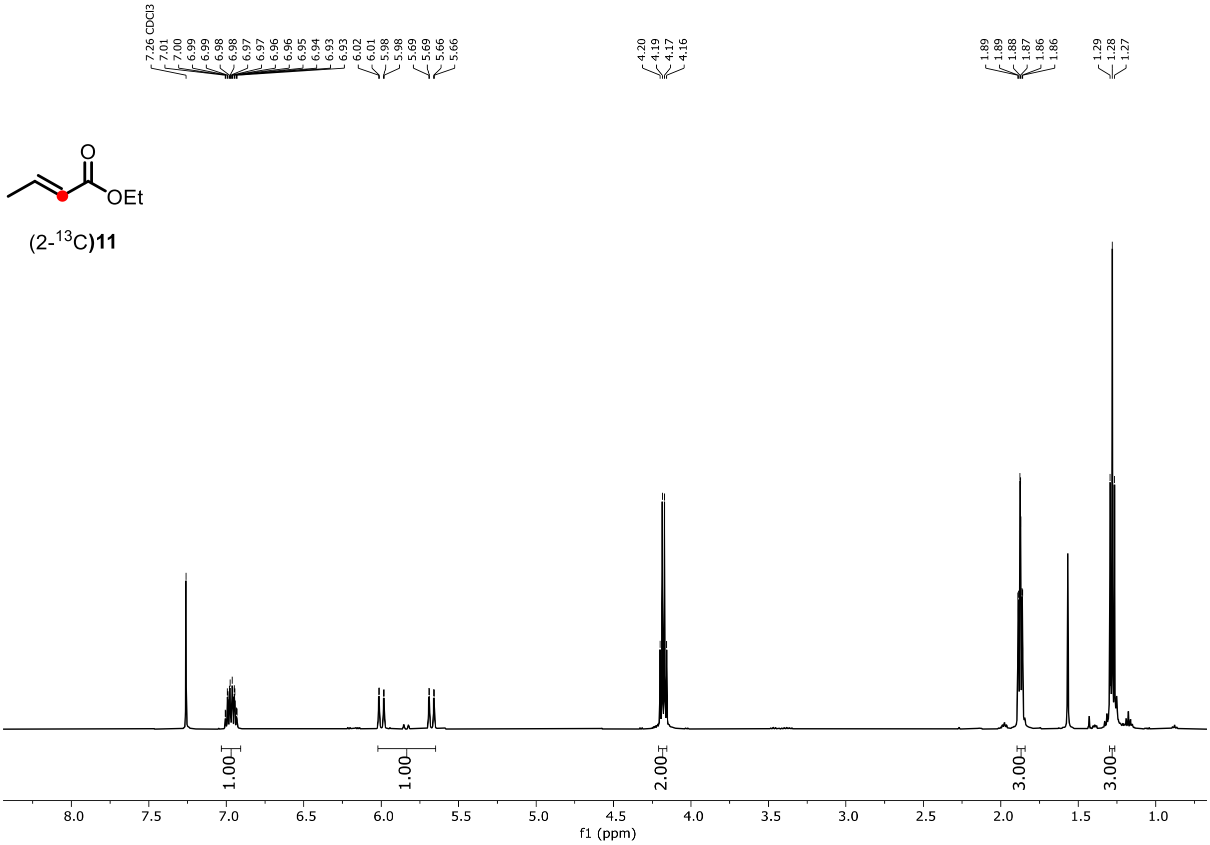
7.26 CDCl3
7.01
7.00
6.99
6.99
6.98
6.98
6.97
6.97
6.96
6.96
6.95
6.94
6.93
6.93
6.02
6.01
5.98
5.98
5.69
5.69
5.66
5.66
4.20
4.19
4.17
4.16
1.89
1.89
1.88
1.87
1.86
1.86
1.29
1.28
1.27
O
OEt
(2-13C)11
1.00
1.00
2.00
3.00
3.00
8.0
7.5
7.0
6.5
6.0
5.5
5.0
4.5
4.0
3.5
3.0
2.5
2.0
1.5
1.0
f1 (ppm)

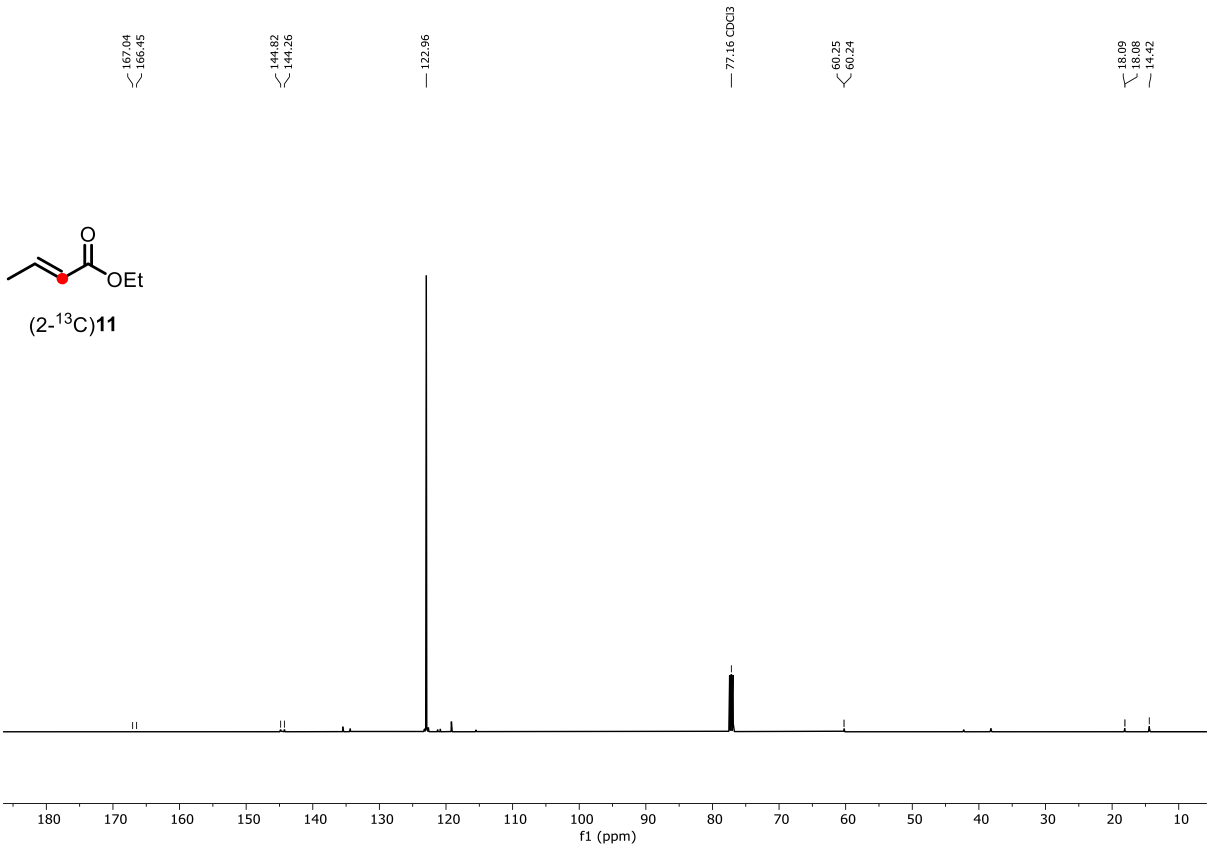
167.04
166.45
144.82
144.26
122.96
77.16 CDCl3
60.25
60.24
18.09
18.08
14.42
O
OEt
(2-13C)11
180
170
160
150
140
130
120
110
100
90
80
70
60
50
40
30
20
10
f1 (ppm)

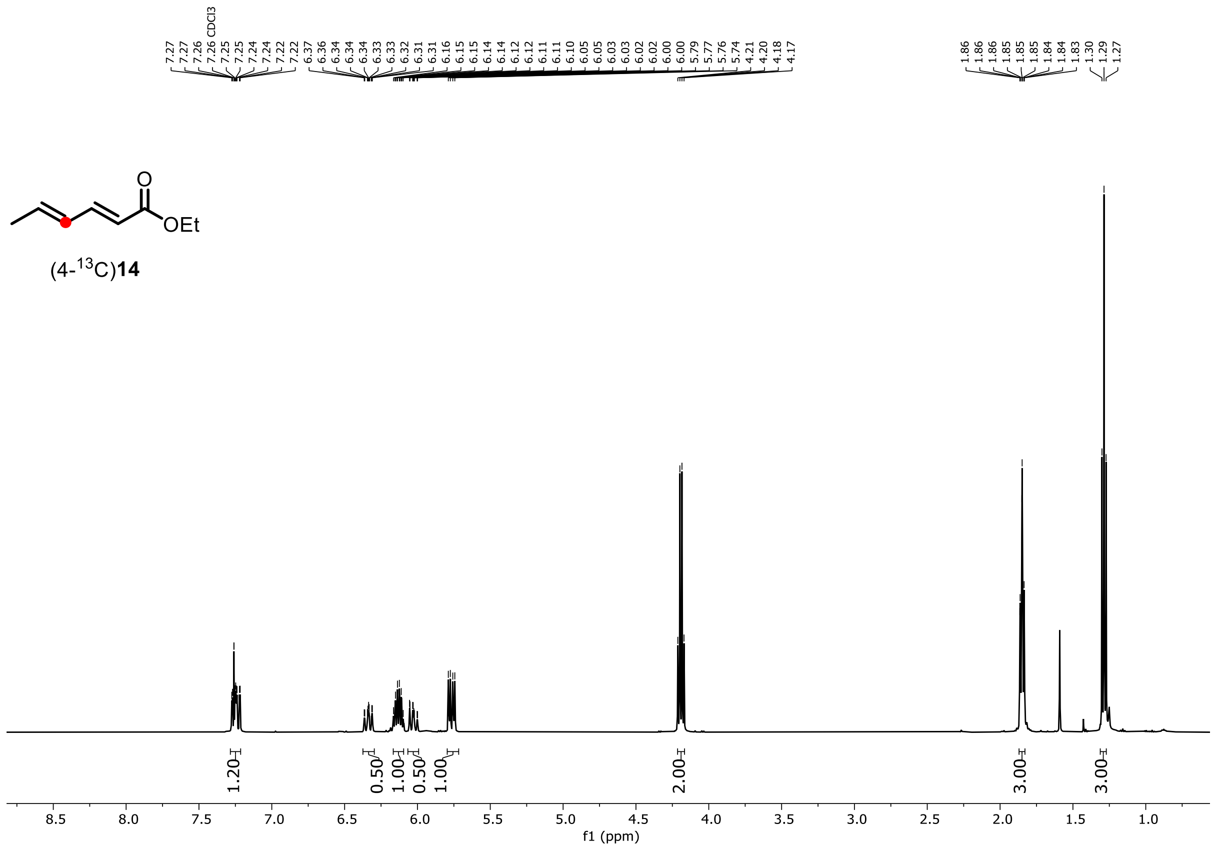

(4-^{13}C)**14**

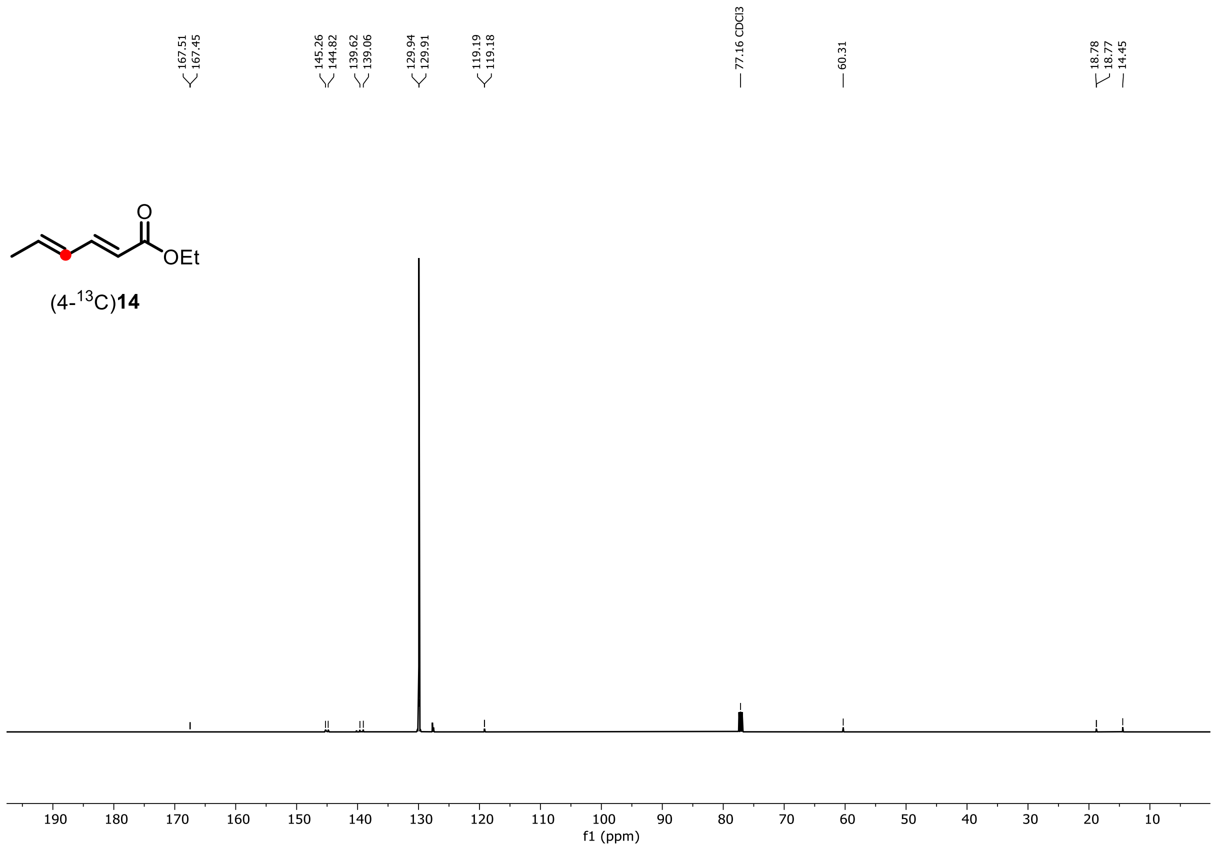

(4-^{13}C)**14**

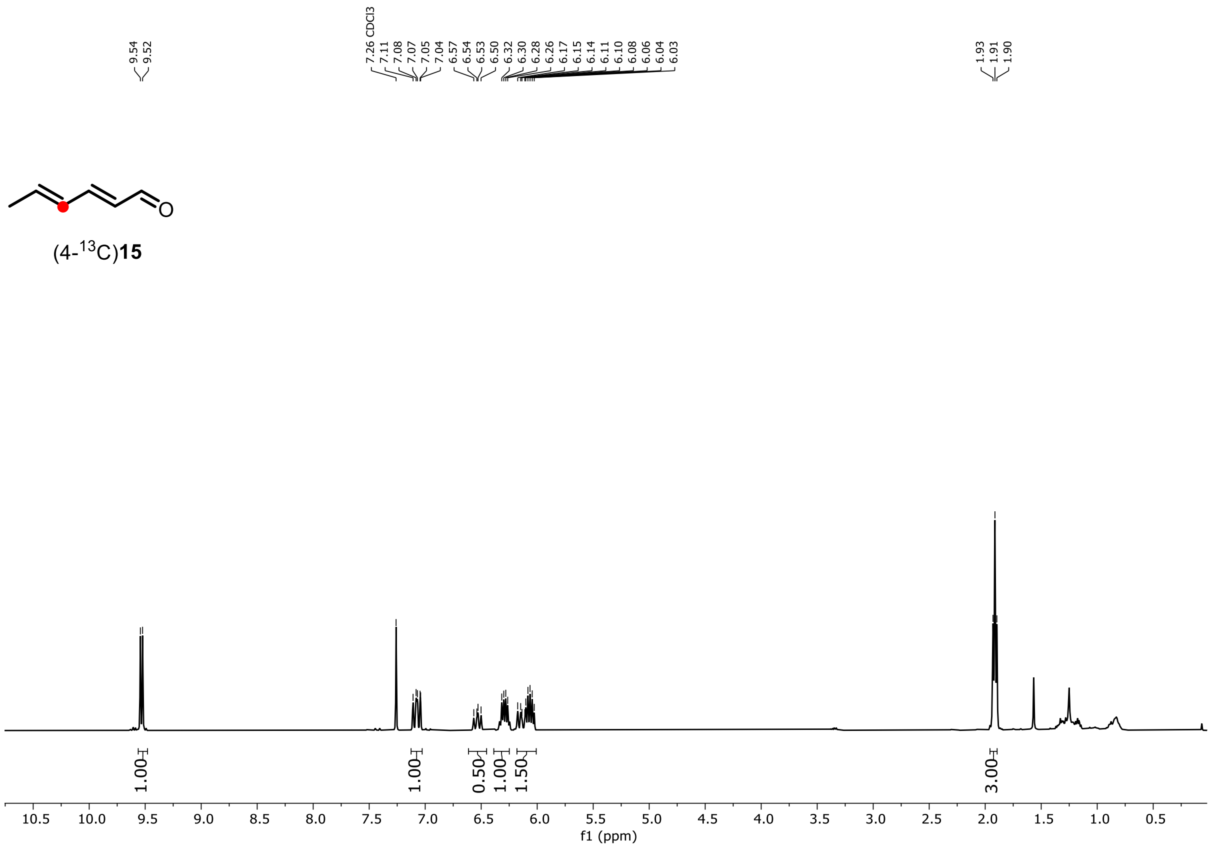

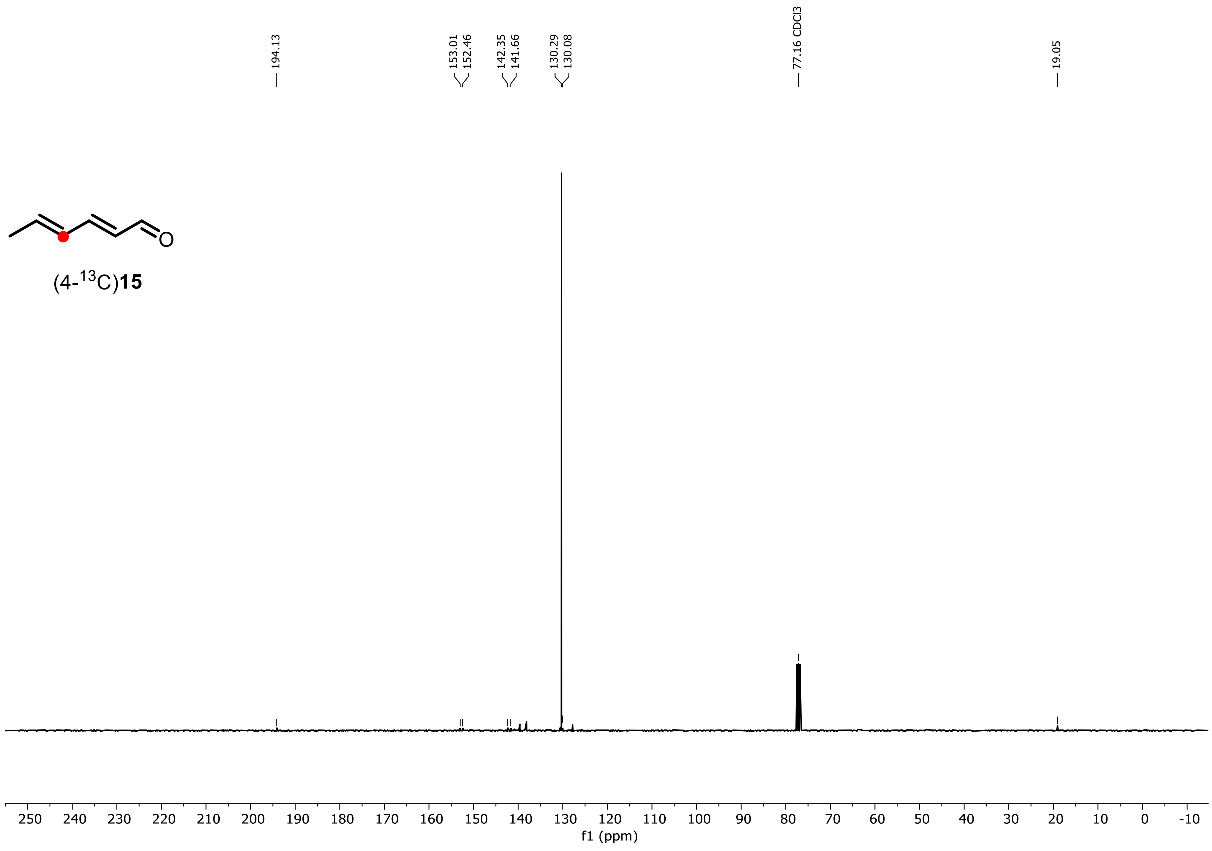

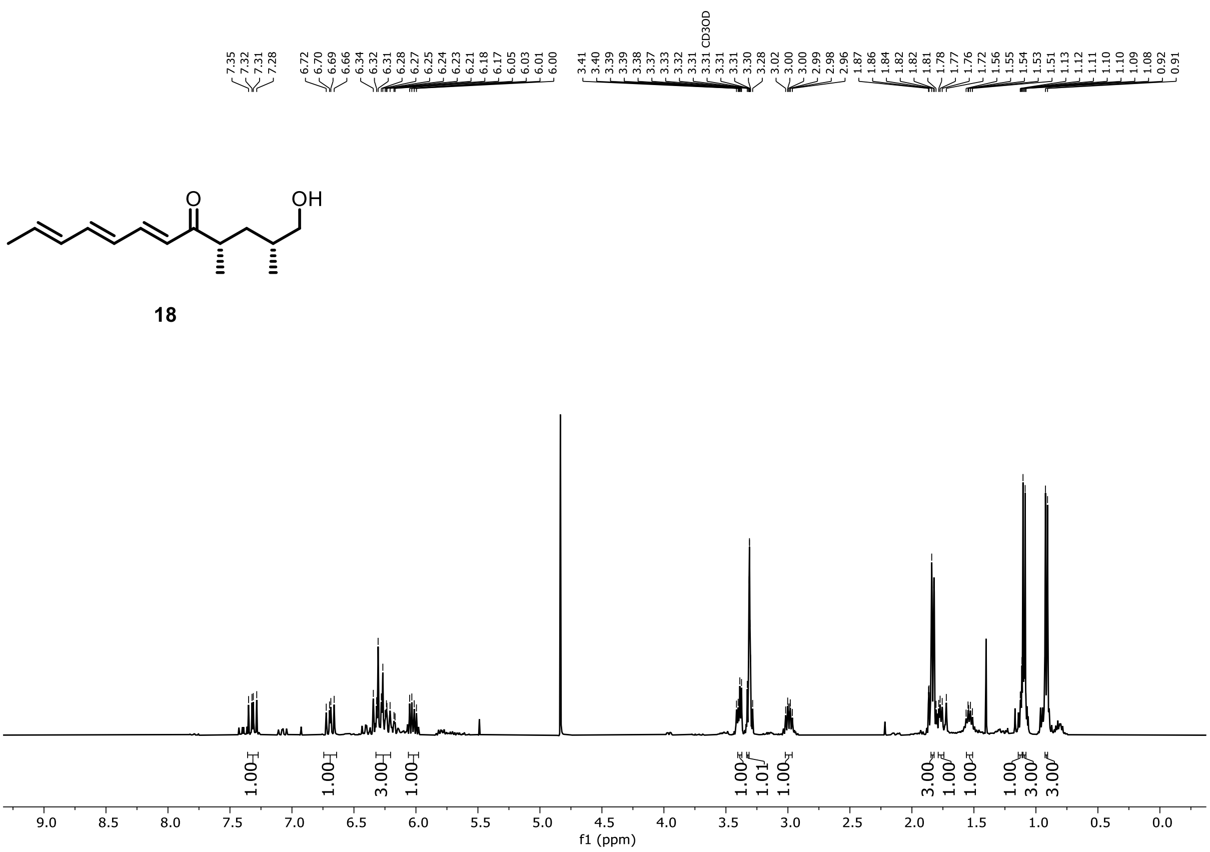
7.35 7.32 7.31 7.28
6.72 6.70 6.69 6.66 6.34 6.32 6.31 6.28 6.27 6.25 6.24 6.23 6.21 6.18 6.17 6.05 6.03 6.01 6.00
3.41 3.40 3.39 3.39 3.38 3.37 3.33 3.32 3.31 3.31 CD3OD 3.31 3.31 3.30 3.28 3.02 3.00 3.00 2.99 2.98 2.96
1.87 1.86 1.84 1.82 1.82 1.81 1.78 1.77 1.76 1.72 1.56 1.55 1.54 1.53 1.51 1.13 1.12 1.11 1.10 1.10 1.09 1.08 0.92 0.91
O
OH
18
1.00 1.00 3.00 1.00 1.00 1.01 1.00 3.00 1.00 1.00 1.00 3.00 3.00
9.0 8.5 8.0 7.5 7.0 6.5 6.0 5.5 5.0 4.5 4.0 3.5 3.0 2.5 2.0 1.5 1.0 0.5 0.0
f1 (ppm)

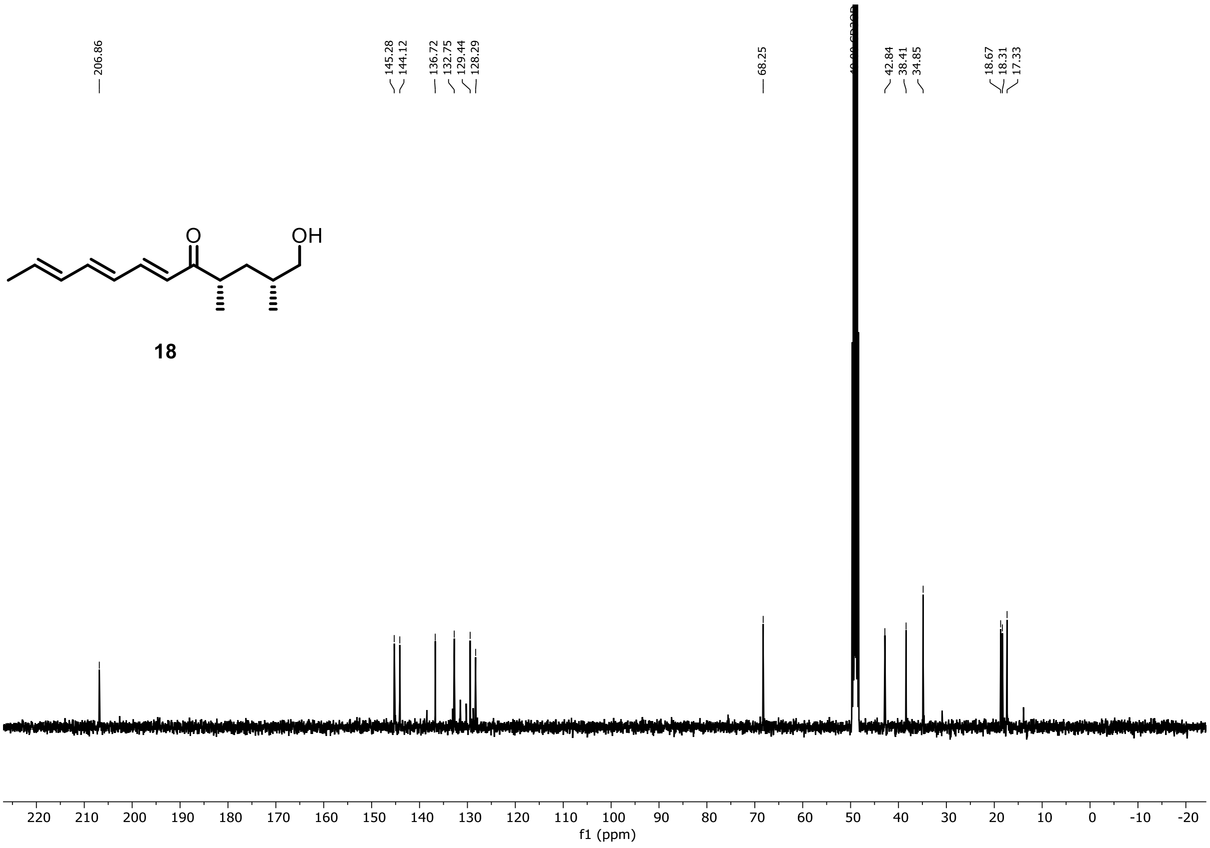
206.86
145.28 144.12 136.72 132.75 129.44 128.29
68.25
42.84 38.41 34.85
18.67 18.31 17.33
O
OH
18
220 210 200 190 180 170 160 150 140 130 120 110 100 90 80 70 60 50 40 30 20 10 0 -10 -20
f1 (ppm)

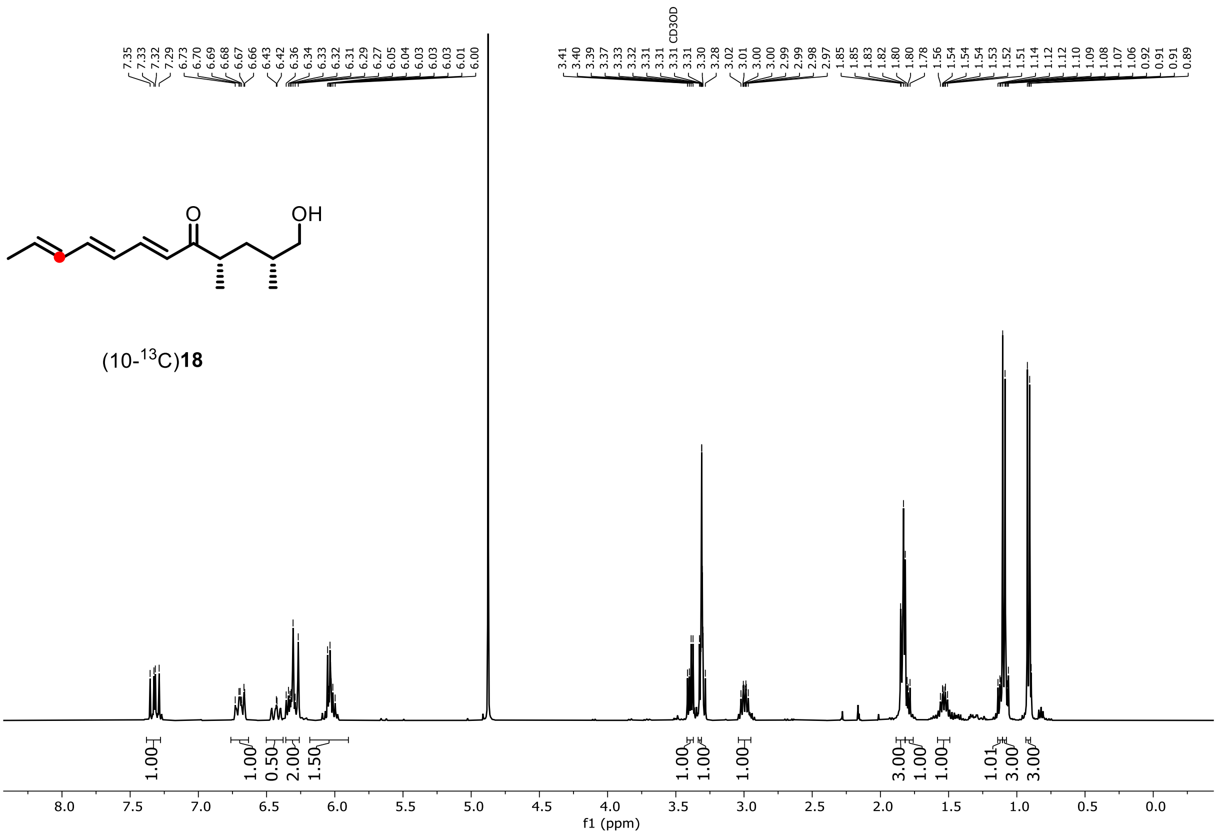
7.35
7.33
7.32
7.29
6.73
6.70
6.69
6.68
6.67
6.66
6.43
6.42
6.36
6.34
6.33
6.32
6.31
6.29
6.27
6.05
6.04
6.03
6.03
6.03
6.01
6.00
3.41
3.40
3.39
3.37
3.33
3.32
3.31
3.31 CD3OD
3.31
3.30
3.28
3.02
3.01
3.00
3.00
2.99
2.99
2.98
2.97
1.85
1.85
1.83
1.82
1.80
1.80
1.78
1.56
1.54
1.54
1.54
1.53
1.52
1.51
1.14
1.12
1.10
1.09
1.08
1.07
1.06
0.92
0.91
0.89
O
OH
(10-13C)18
1.00
1.00
0.50
2.00
1.50
1.00
1.00
1.00
3.00
1.00
1.00
1.01
3.00
3.00
8.0
7.5
7.0
6.5
6.0
5.5
5.0
4.5
4.0
3.5
3.0
2.5
2.0
1.5
1.0
0.5
0.0
f1 (ppm)

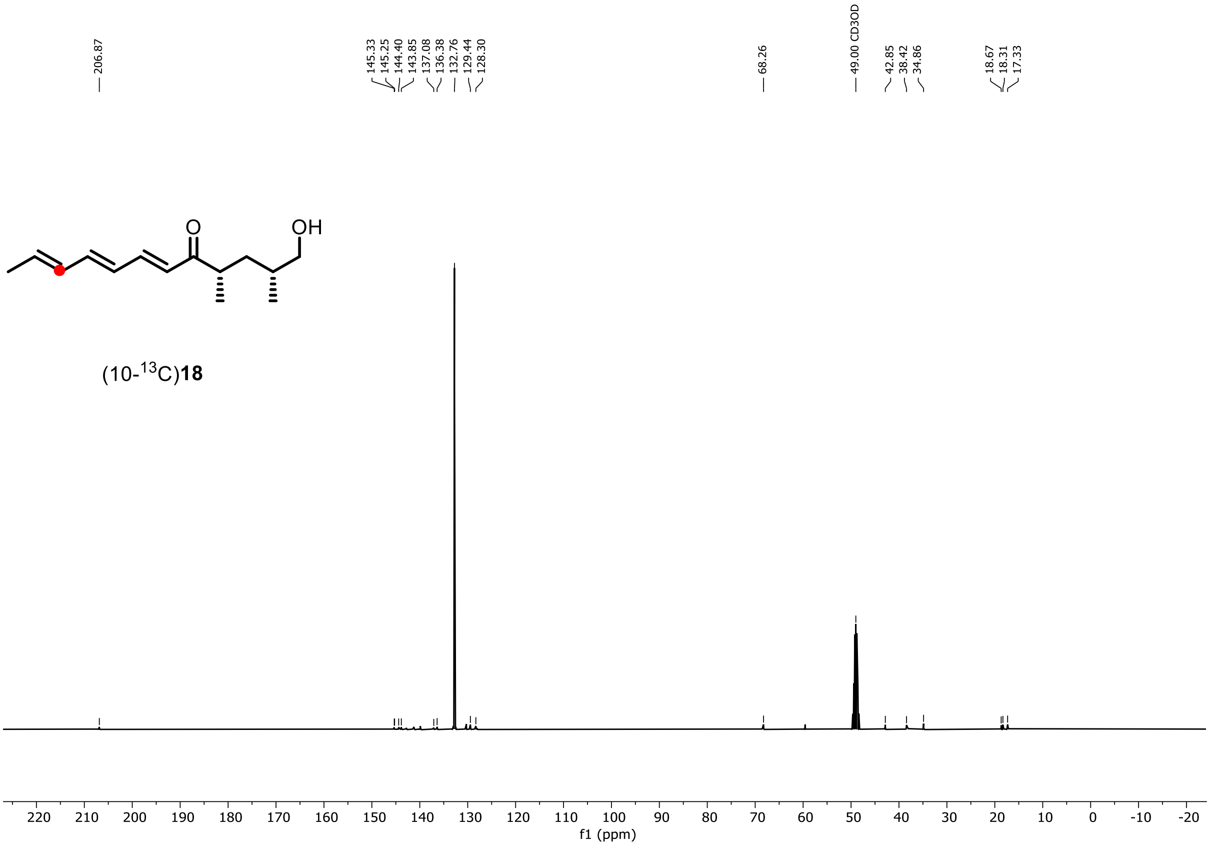
206.87
145.33
145.25
144.40
143.85
137.08
136.38
132.76
129.44
128.30
68.26
49.00 CD3OD
42.85
38.42
34.86
18.67
18.31
17.33
O
OH
(10-13C)18
220
210
200
190
180
170
160
150
140
130
120
110
100
90
80
70
60
50
40
30
20
10
0
-10
-20
f1 (ppm)

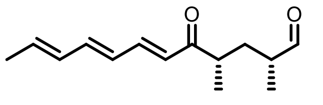
O
O

19

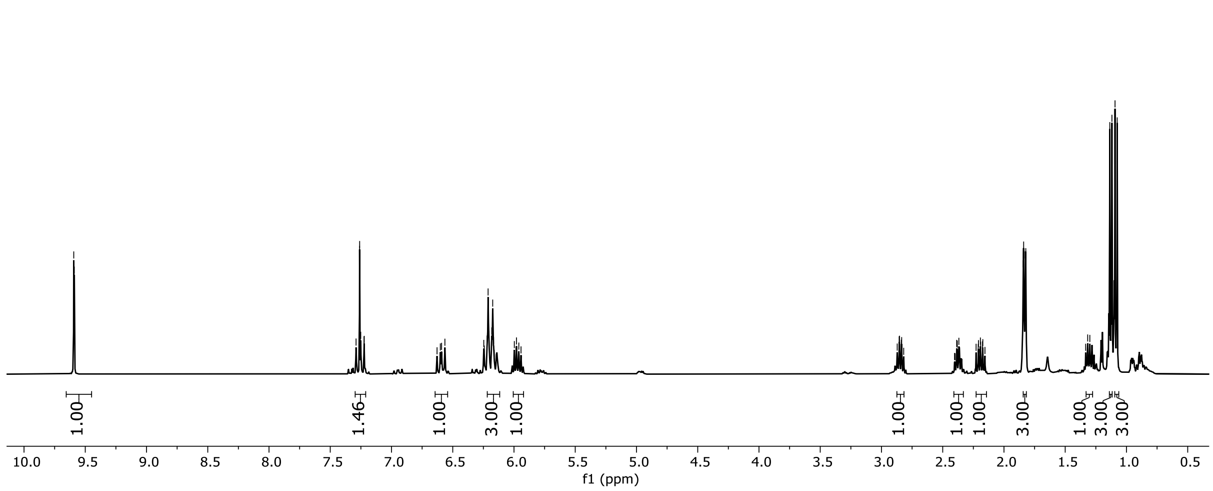
9.59
9.59
7.29
7.26
7.26 CDCl3
7.25
7.22
7.22
7.22
6.63
6.60
6.59
6.56
6.25
6.24
6.22
6.21
6.20
6.18
6.18
6.17
6.17
6.16
6.00
5.98
5.96
5.94
2.87
2.86
2.85
2.84
2.84
2.82
2.40
2.40
2.39
2.38
2.37
2.23
2.21
2.21
2.19
2.19
2.18
2.17
2.16
1.84
1.84
1.83
1.82
1.33
1.32
1.30
1.14
1.12
1.09
1.08
1.00
1.46
1.00
3.00
1.00
1.00
1.00
1.00
3.00
1.00
3.00
3.00
10.0 9.5 9.0 8.5 8.0 7.5 7.0 6.5 6.0 5.5 5.0 4.5 4.0 3.5 3.0 2.5 2.0 1.5 1.0 0.5
f1 (ppm)

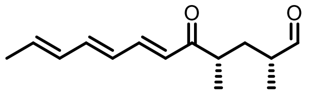
O
O

19

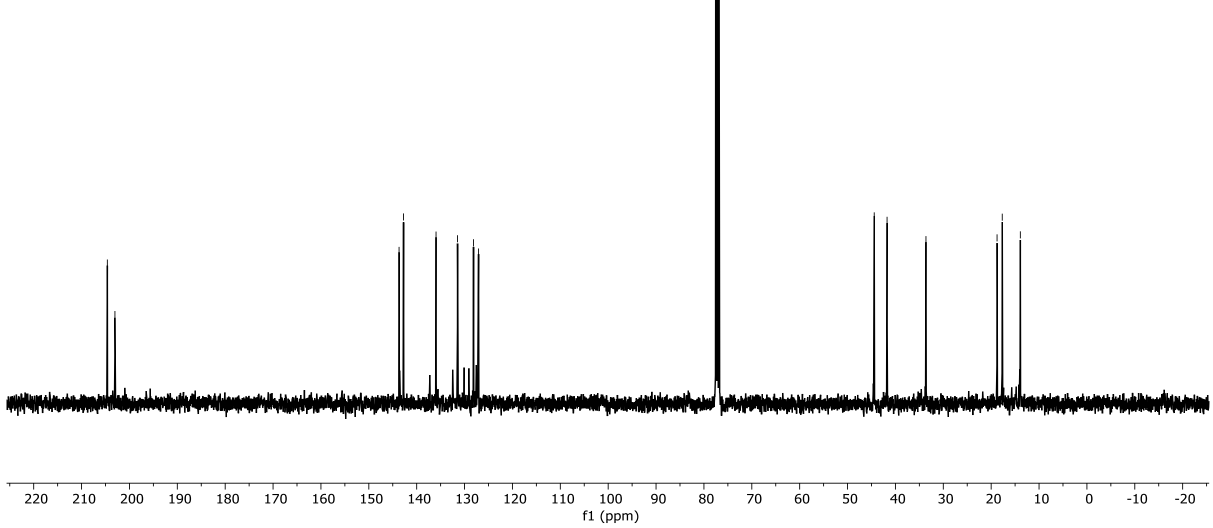
204.62
203.03
143.68
142.74
135.96
131.46
128.13
127.07
44.43
41.74
33.62
18.77
17.69
13.90
220 210 200 190 180 170 160 150 140 130 120 110 100 90 80 70 60 50 40 30 20 10 0 -10 -20
f1 (ppm)

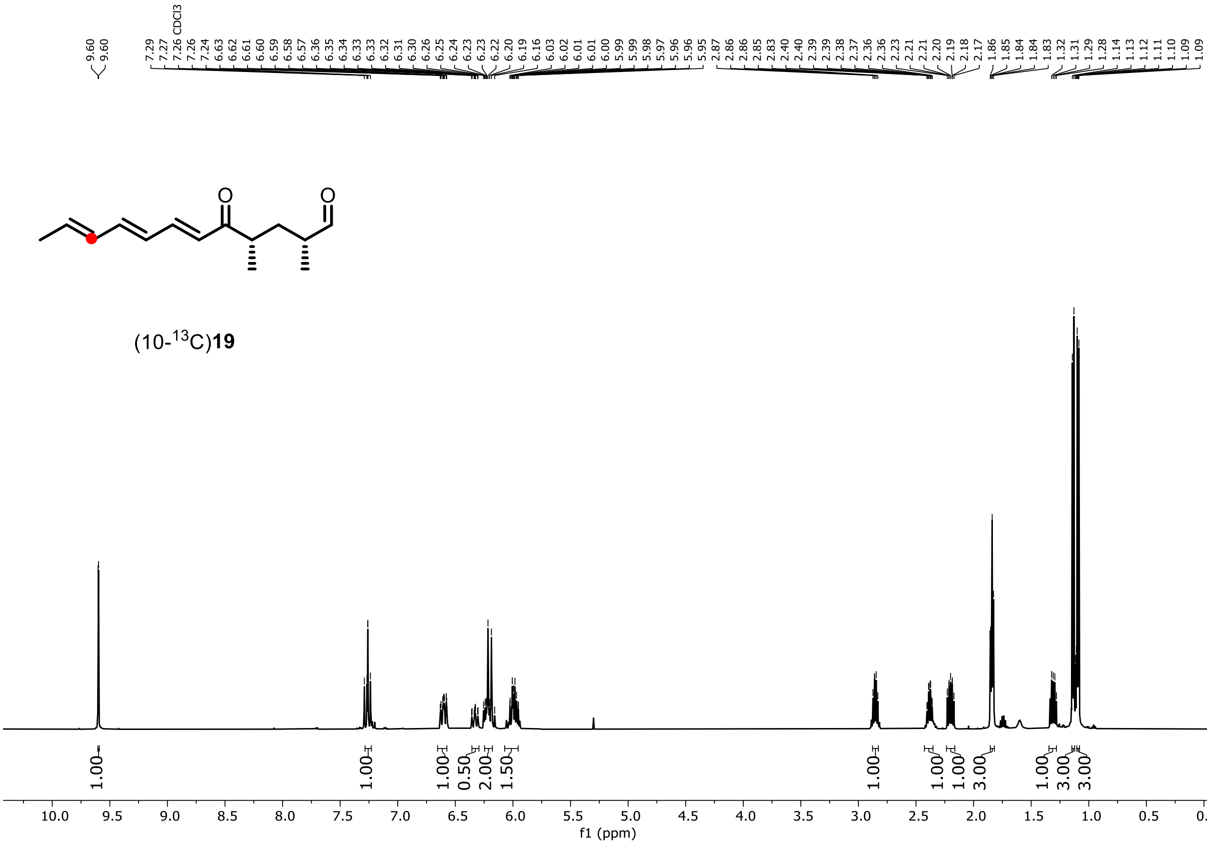
(10-13C)19
9.60
9.60
7.26 CDCl3
1.00
1.00
1.00
0.50
2.00
1.50
1.00
1.00
1.00
3.00
1.00
3.00
3.00
f1 (ppm)

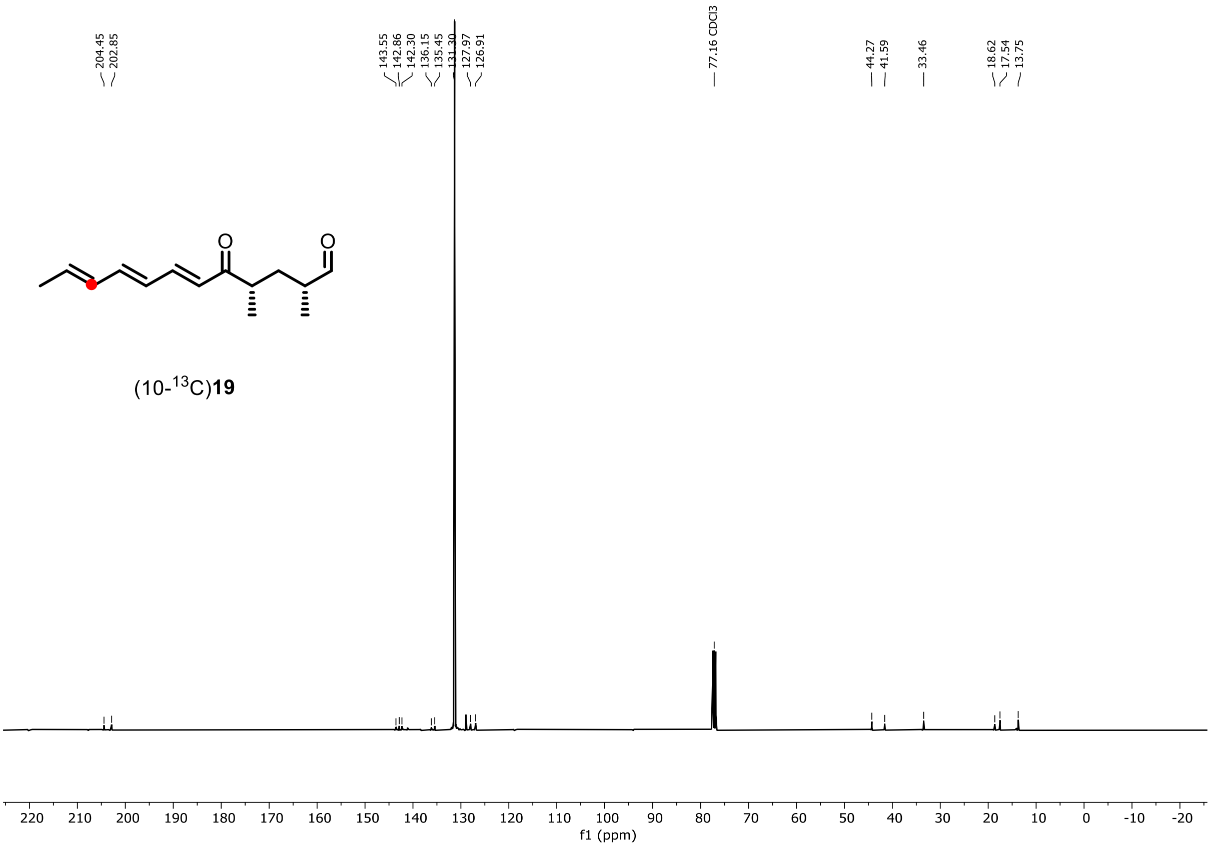
(10-13C)19
204.45
202.85
143.55
142.86
142.30
136.15
135.45
131.30
127.97
126.91
77.16 CDCl3
44.27
41.59
33.46
18.62
17.54
13.75
f1 (ppm)

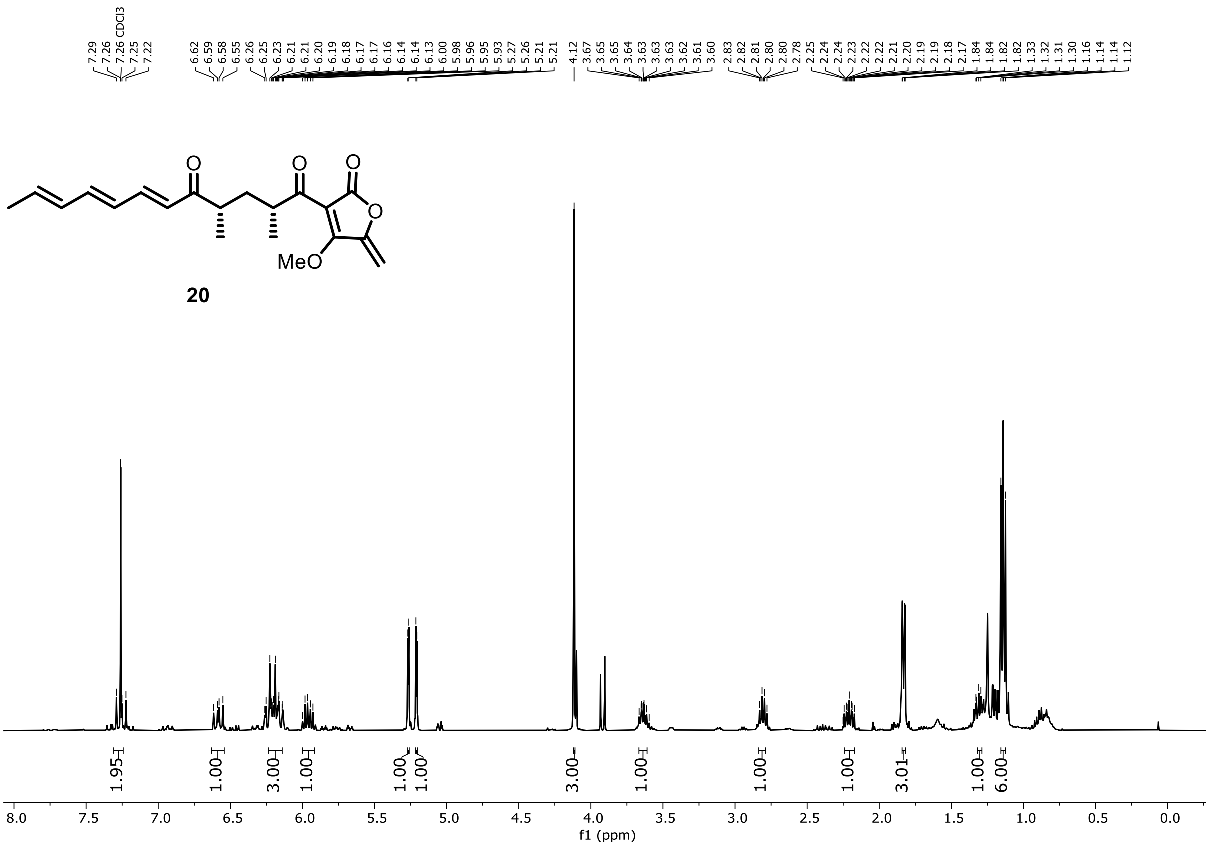
7.29
7.26
7.26 CDCl3
7.25
7.22
6.62
6.59
6.58
6.55
6.26
6.25
6.23
6.21
6.21
6.20
6.19
6.18
6.17
6.17
6.16
6.14
6.14
6.13
6.00
5.98
5.96
5.95
5.93
5.27
5.26
5.21
5.21
4.12
3.67
3.65
3.64
3.63
3.63
3.63
3.62
3.61
3.60
2.83
2.82
2.81
2.80
2.80
2.78
2.25
2.24
2.24
2.23
2.22
2.22
2.22
2.21
2.20
2.19
2.19
2.18
2.17
1.84
1.84
1.82
1.82
1.33
1.32
1.31
1.30
1.16
1.14
1.14
1.12
O
O
O
O
MeO
20
1.95
1.00
3.00
1.00
1.00
1.00
3.00
1.00
1.00
1.00
3.01
1.00
6.00
f1 (ppm)

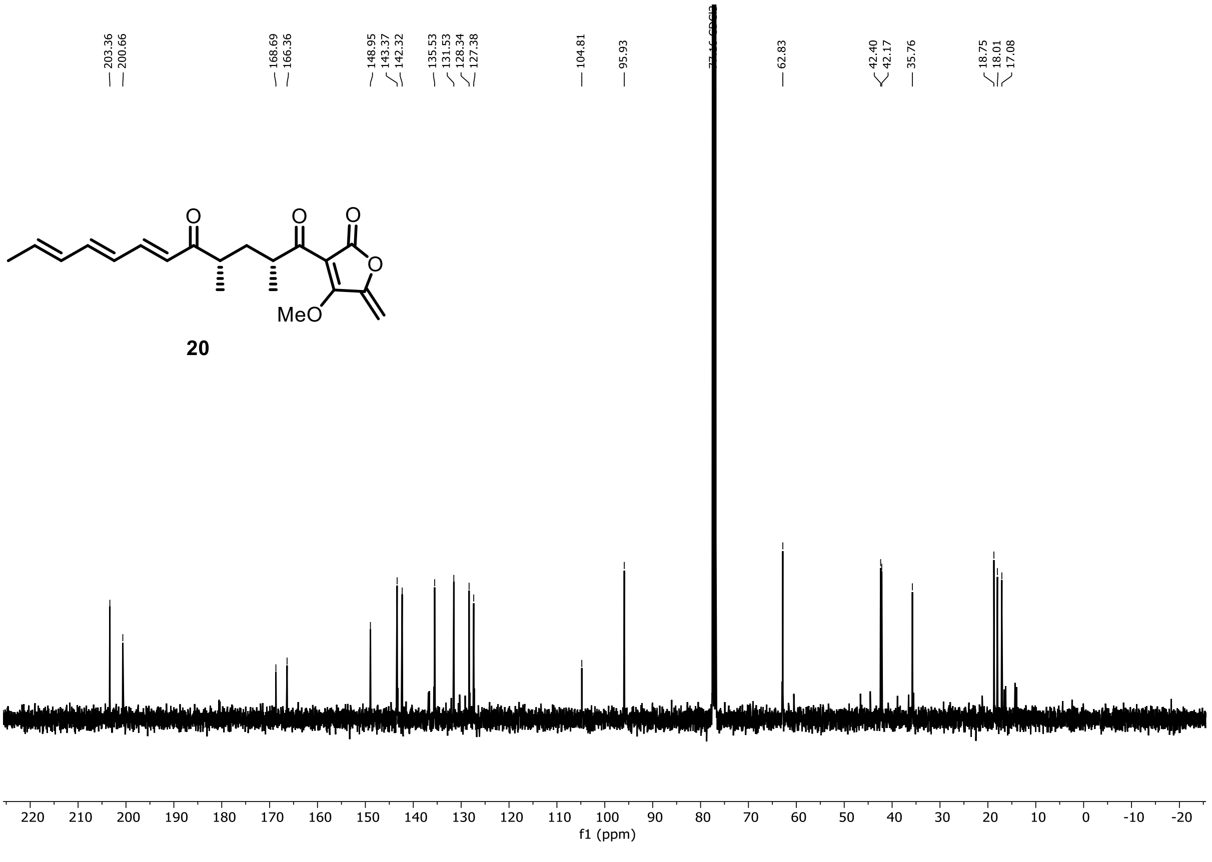
203.36
200.66
168.69
166.36
148.95
143.37
142.32
135.53
131.53
128.34
127.38
104.81
95.93
62.83
42.40
42.17
35.76
18.75
18.01
17.08
O
O
O
O
MeO
20
f1 (ppm)

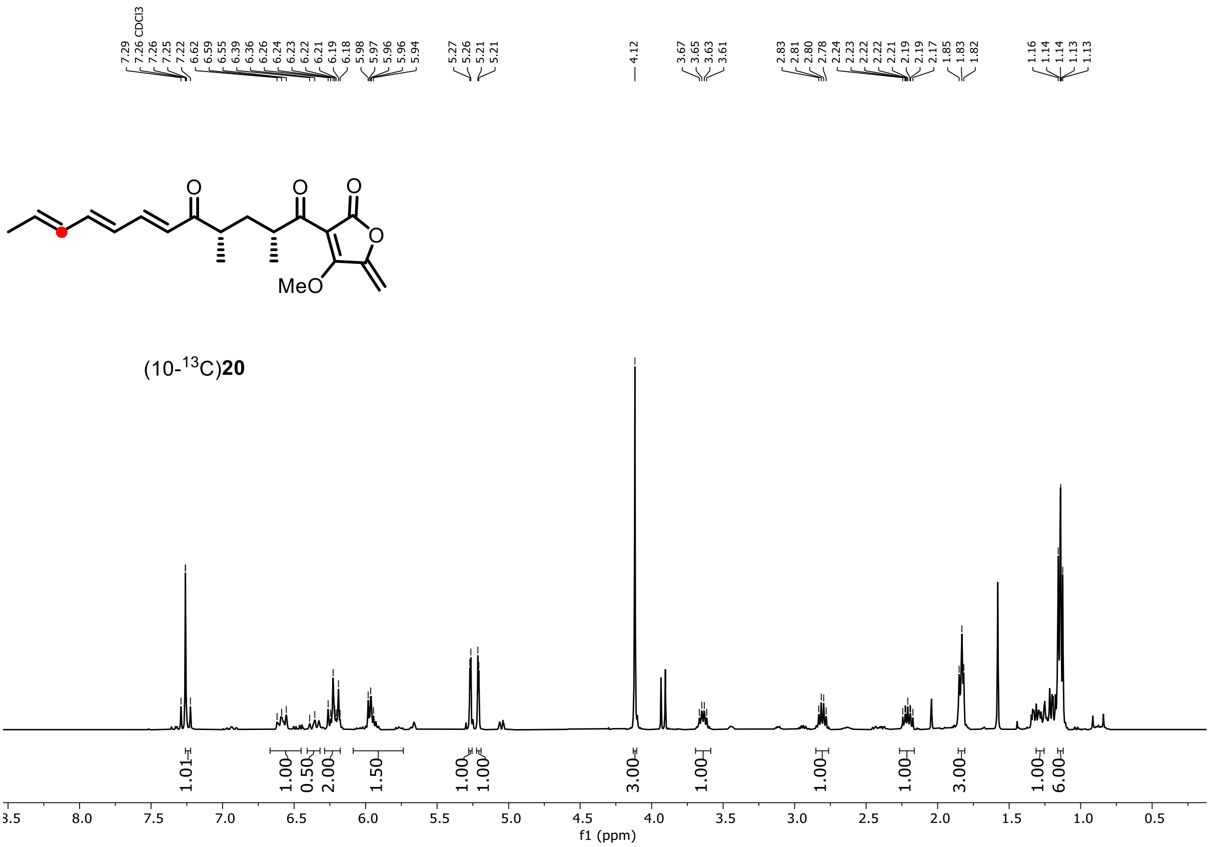

(10-^{13}C)**20**

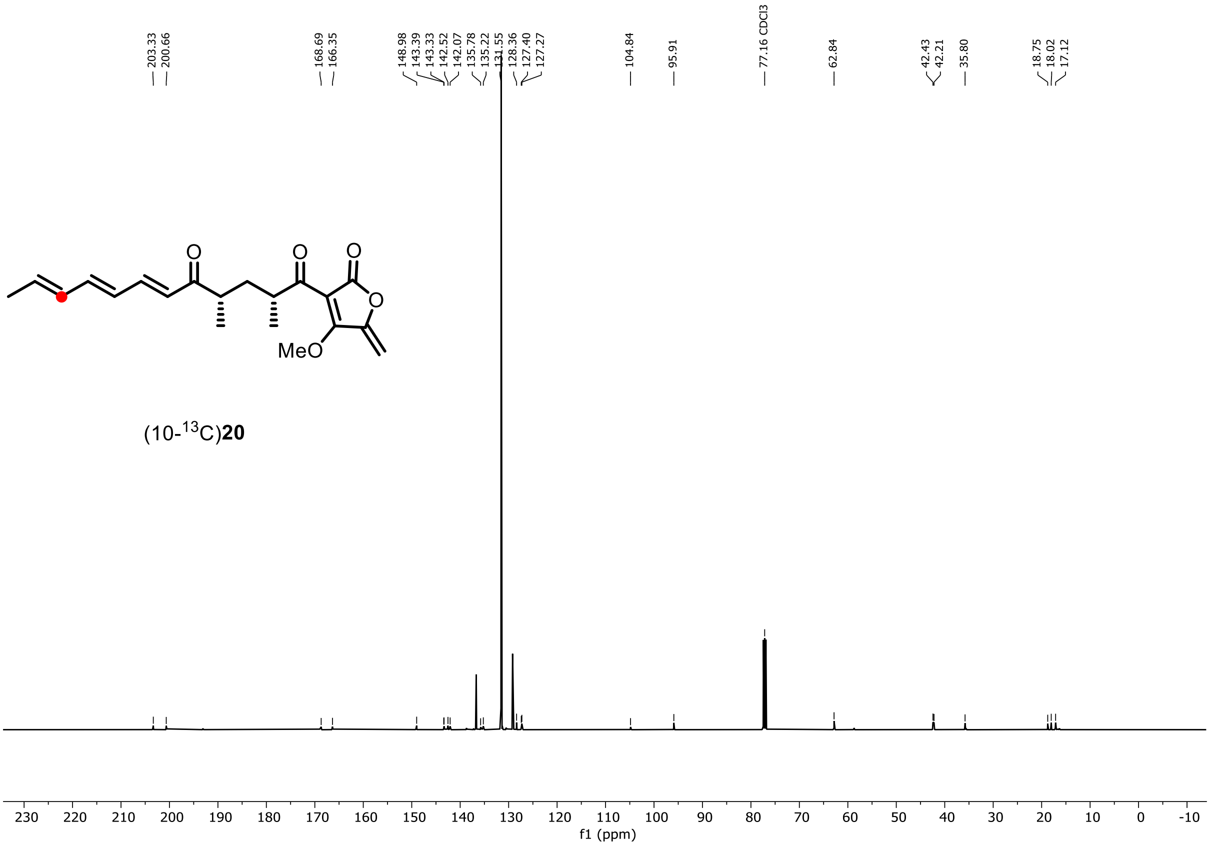

(10-^{13}C)**20**

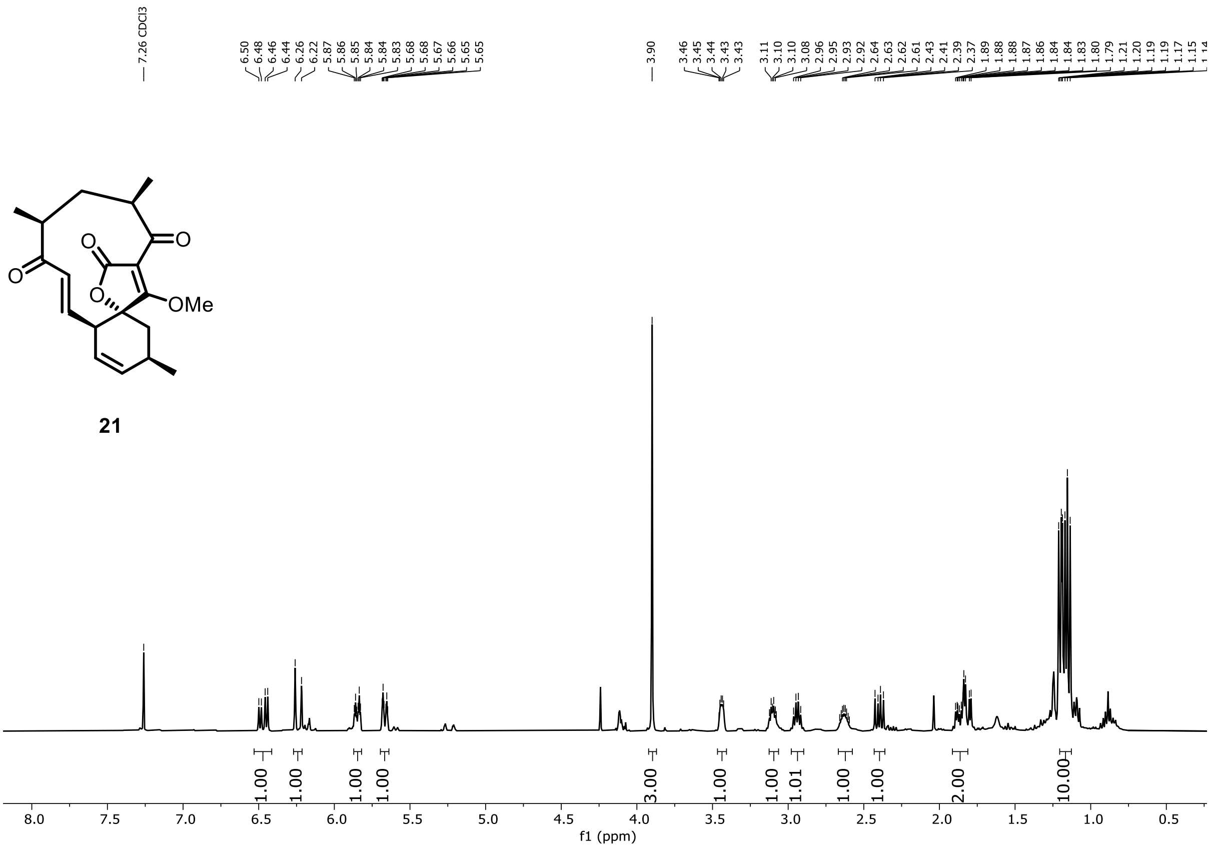
7.26 CDCl3
6.50
6.48
6.46
6.44
6.26
6.22
5.87
5.86
5.85
5.84
5.84
5.83
5.68
5.68
5.67
5.66
5.65
5.65
3.90
3.46
3.45
3.44
3.43
3.43
3.11
3.10
3.10
3.08
2.96
2.95
2.93
2.92
2.64
2.63
2.62
2.61
2.43
2.41
2.39
2.37
1.89
1.88
1.88
1.87
1.86
1.84
1.84
1.83
1.80
1.79
1.21
1.20
1.19
1.17
1.15
O
O
O
O
OMe
21
1.00
1.00
1.00
1.00
3.00
1.00
1.00
1.01
1.00
1.00
2.00
10.00
8.0
7.5
7.0
6.5
6.0
5.5
5.0
4.5
4.0
3.5
3.0
2.5
2.0
1.5
1.0
0.5
f1 (ppm)

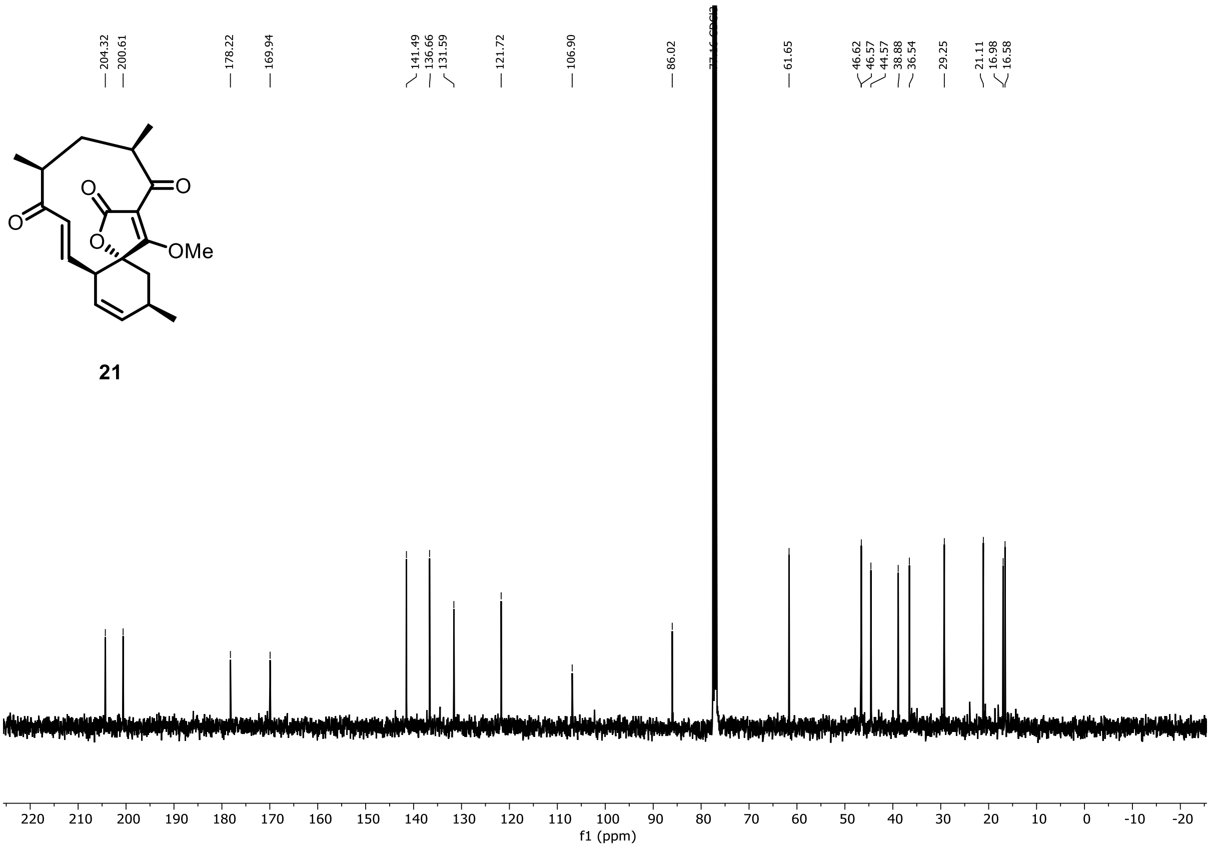
204.32
200.61
178.22
169.94
141.49
136.66
131.59
121.72
106.90
86.02
61.65
46.62
46.57
44.57
38.88
36.54
29.25
21.11
16.98
16.58
O
O
O
O
OMe
21
220
210
200
190
180
170
160
150
140
130
120
110
100
90
80
70
60
50
40
30
20
10
0
-10
-20
f1 (ppm)

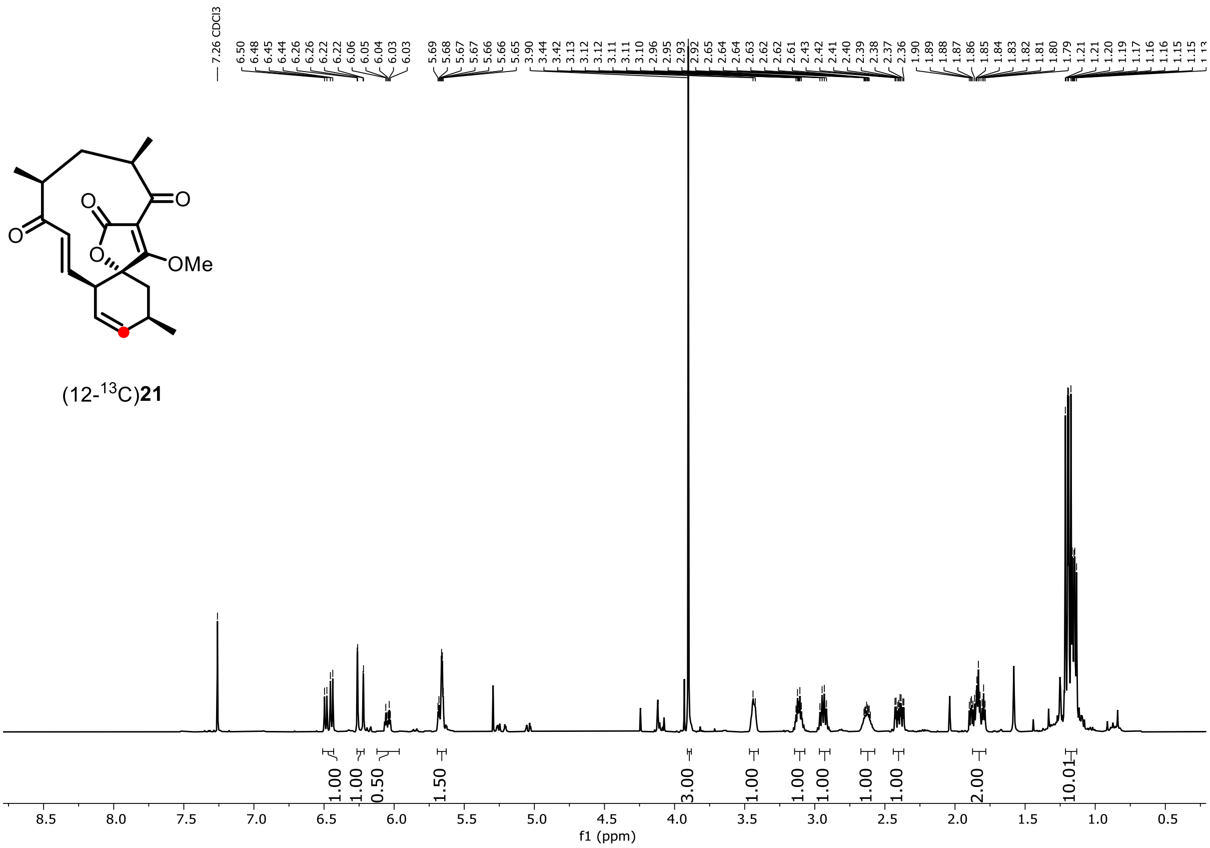
7.26 CDCl3
6.50 6.48 6.45 6.44 6.26 6.26 6.22 6.22 6.06 6.05 6.04 6.03 6.03
5.69 5.68 5.67 5.67 5.66 5.66 5.65
3.90 3.44 3.42 3.13 3.12 3.12 3.11 3.11 3.10 2.96 2.95 2.93 2.92 2.65 2.64 2.64 2.63 2.62 2.62 2.61 2.43 2.42 2.41 2.40 2.39 2.38 2.37 2.36
1.90 1.89 1.88 1.87 1.86 1.85 1.84 1.83 1.82 1.81 1.80 1.79 1.21 1.21 1.20 1.19 1.17 1.16 1.15 1.15 [illegible]
O
O
O
OMe
(12-^{13}C)21
1.00
1.00
0.50
1.50
3.00
1.00
1.00
1.00
1.00
1.00
2.00
10.01
8.5 8.0 7.5 7.0 6.5 6.0 5.5 5.0 4.5 4.0 3.5 3.0 2.5 2.0 1.5 1.0 0.5
f1 (ppm)

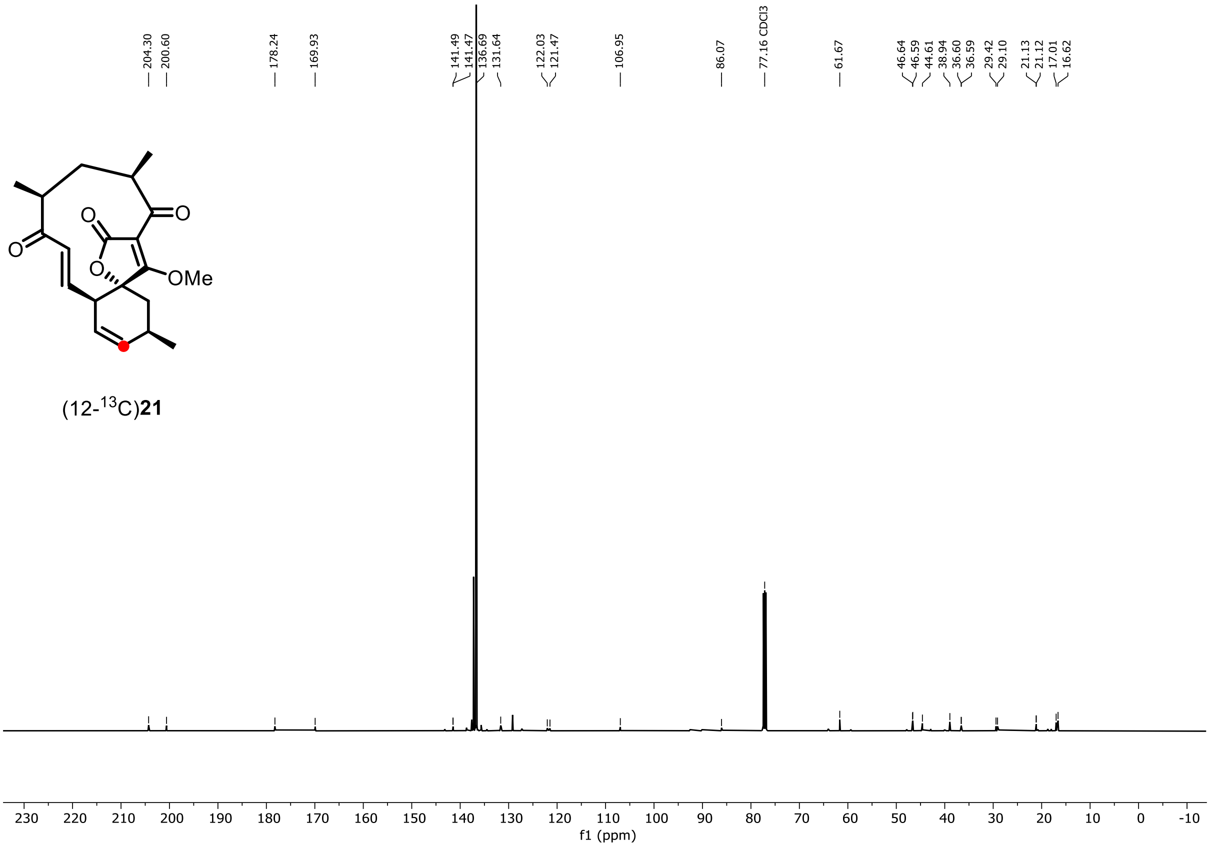
204.30
200.60
178.24
169.93
141.49 141.47 136.69 131.64
122.03 121.47
106.95
86.07
77.16 CDCl3
61.67
46.64 46.59 44.61 38.94 36.60 36.59 29.42 29.10 21.13 21.12 17.01 16.62
O
O
O
OMe
(12-^{13}C)21
230 220 210 200 190 180 170 160 150 140 130 120 110 100 90 80 70 60 50 40 30 20 10 0 -10
f1 (ppm)

SUPPORTING INFORMATION

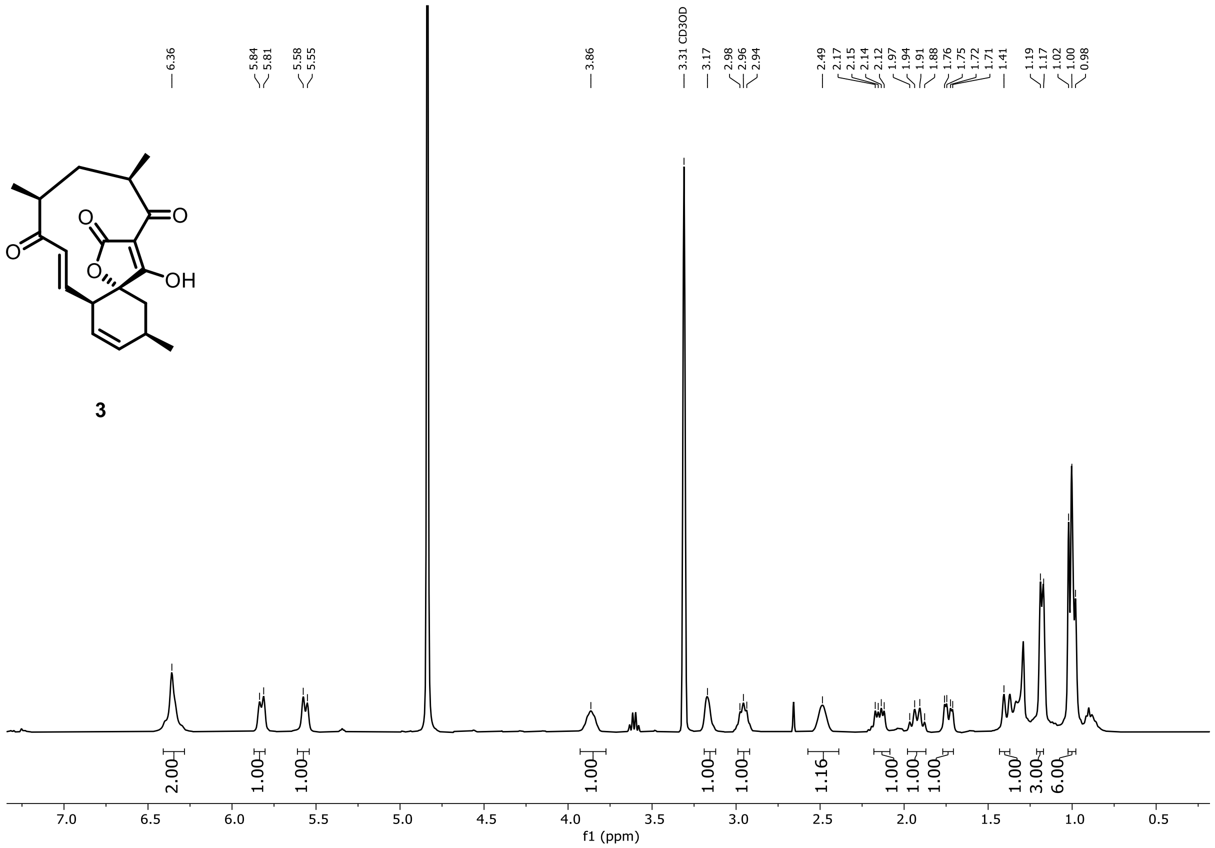

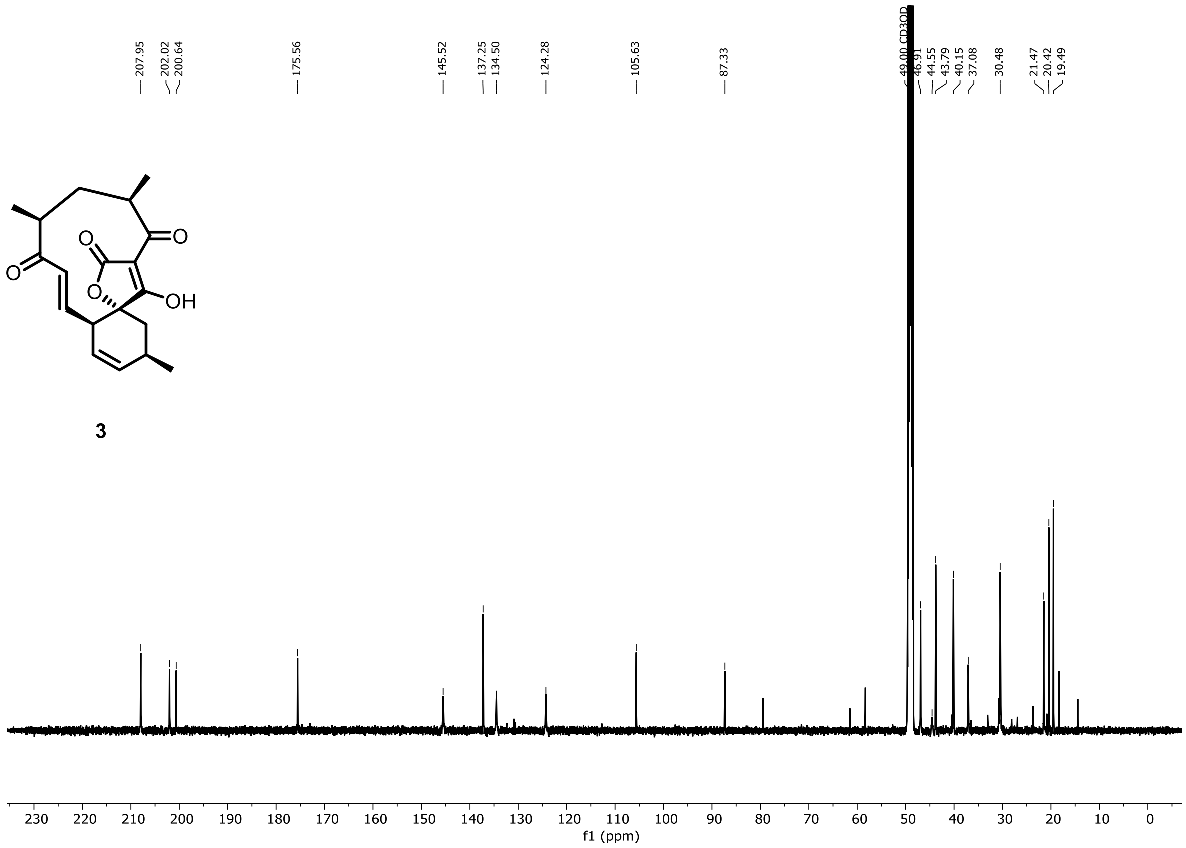

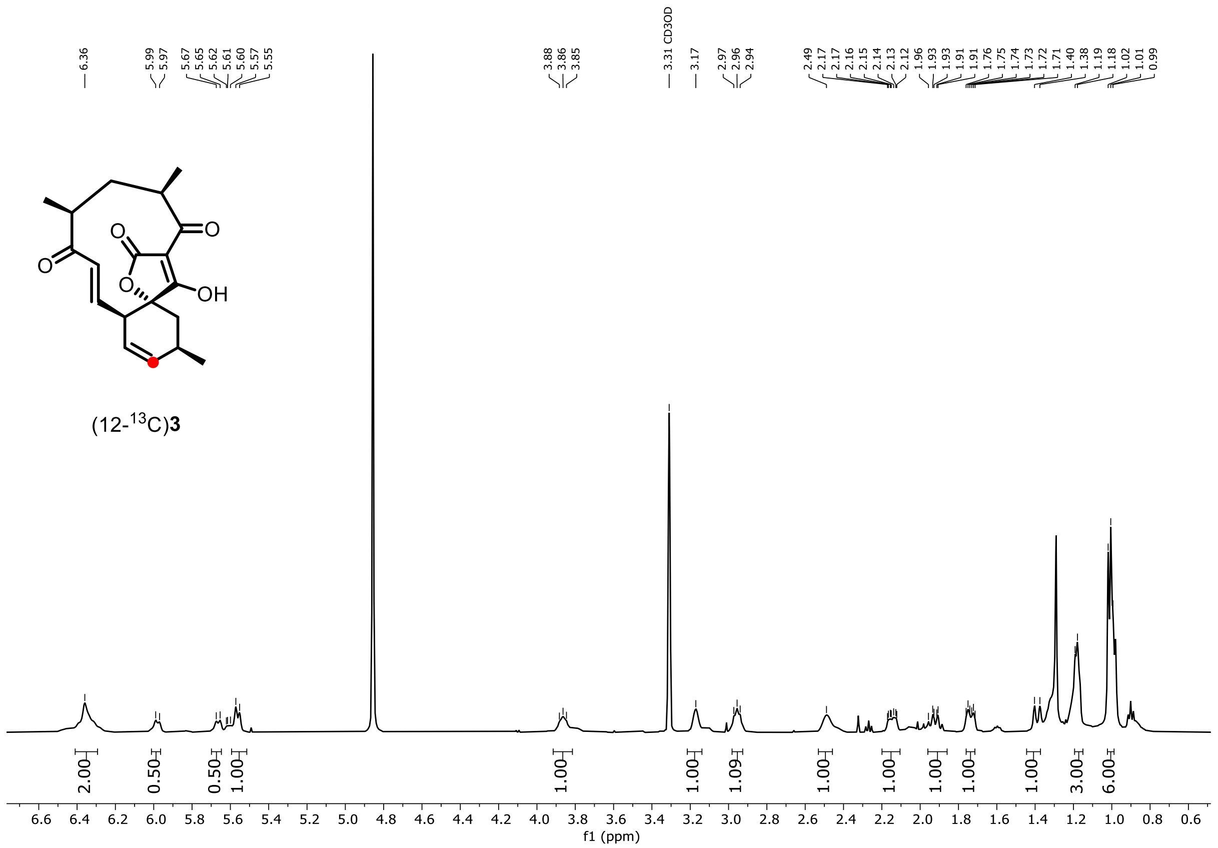
6.36
5.99
5.97
5.67
5.65
5.62
5.61
5.60
5.57
5.55
3.88
3.86
3.85
3.31 CD3OD
3.17
2.97
2.96
2.94
2.49
2.17
2.17
2.16
2.15
2.14
2.13
2.12
1.96
1.93
1.93
1.91
1.91
1.76
1.75
1.74
1.73
1.72
1.71
1.40
1.38
1.19
1.18
1.02
1.01
0.99
(12-13C)3
2.00
0.50
0.50
1.00
1.00
1.00
1.09
1.00
1.00
1.00
1.00
1.00
3.00
6.00
6.6 6.4 6.2 6.0 5.8 5.6 5.4 5.2 5.0 4.8 4.6 4.4 4.2 4.0 3.8 3.6 3.4 3.2 3.0 2.8 2.6 2.4 2.2 2.0 1.8 1.6 1.4 1.2 1.0 0.8 0.6
f1 (ppm)

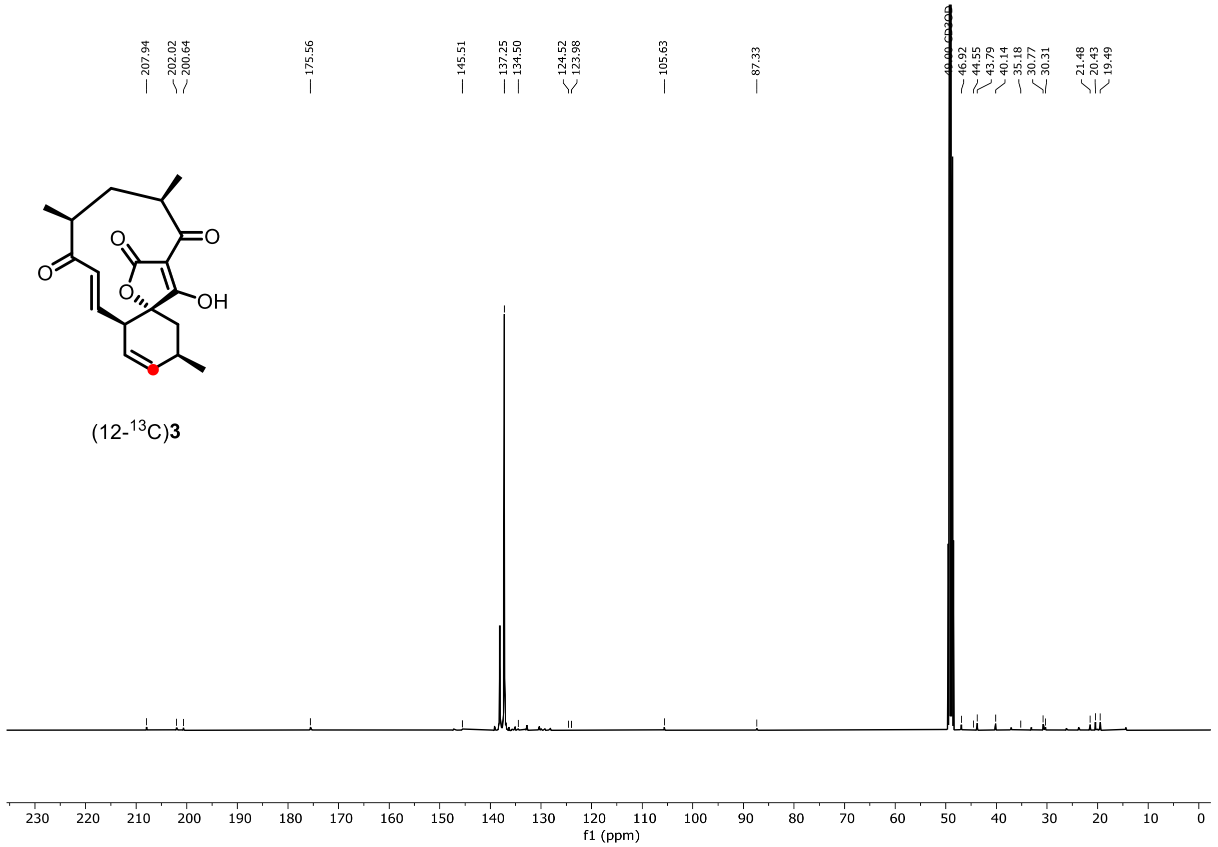
207.94
202.02
200.64
175.56
145.51
137.25
134.50
124.52
123.98
105.63
87.33
49.00 CD3OD
46.92
44.55
43.79
40.14
35.18
30.77
30.31
21.48
20.43
19.49
(12-13C)3
230 220 210 200 190 180 170 160 150 140 130 120 110 100 90 80 70 60 50 40 30 20 10 0
f1 (ppm)

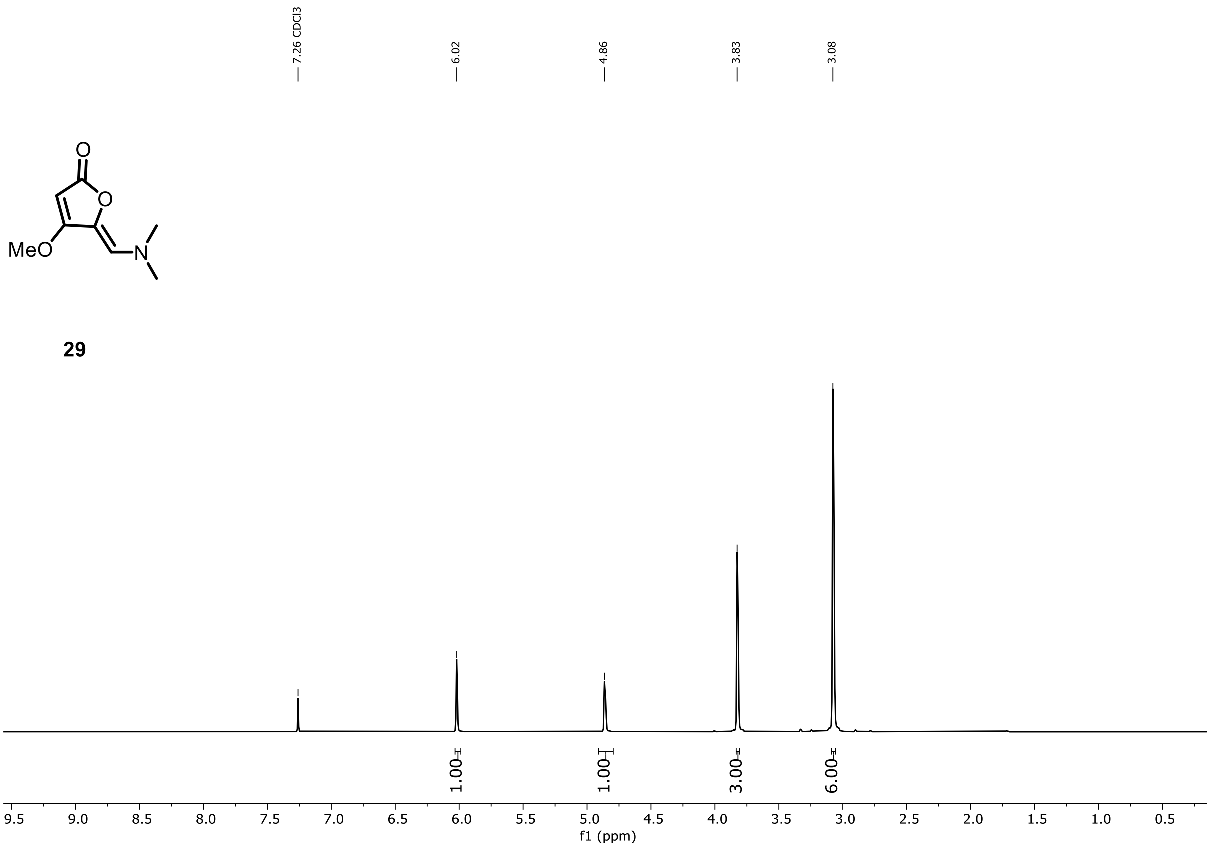
7.26 CDCl3
6.02
4.86
3.83
3.08
O
O
MeO
N
29
1.00
1.00
3.00
6.00
9.5 9.0 8.5 8.0 7.5 7.0 6.5 6.0 5.5 5.0 4.5 4.0 3.5 3.0 2.5 2.0 1.5 1.0 0.5
f1 (ppm)

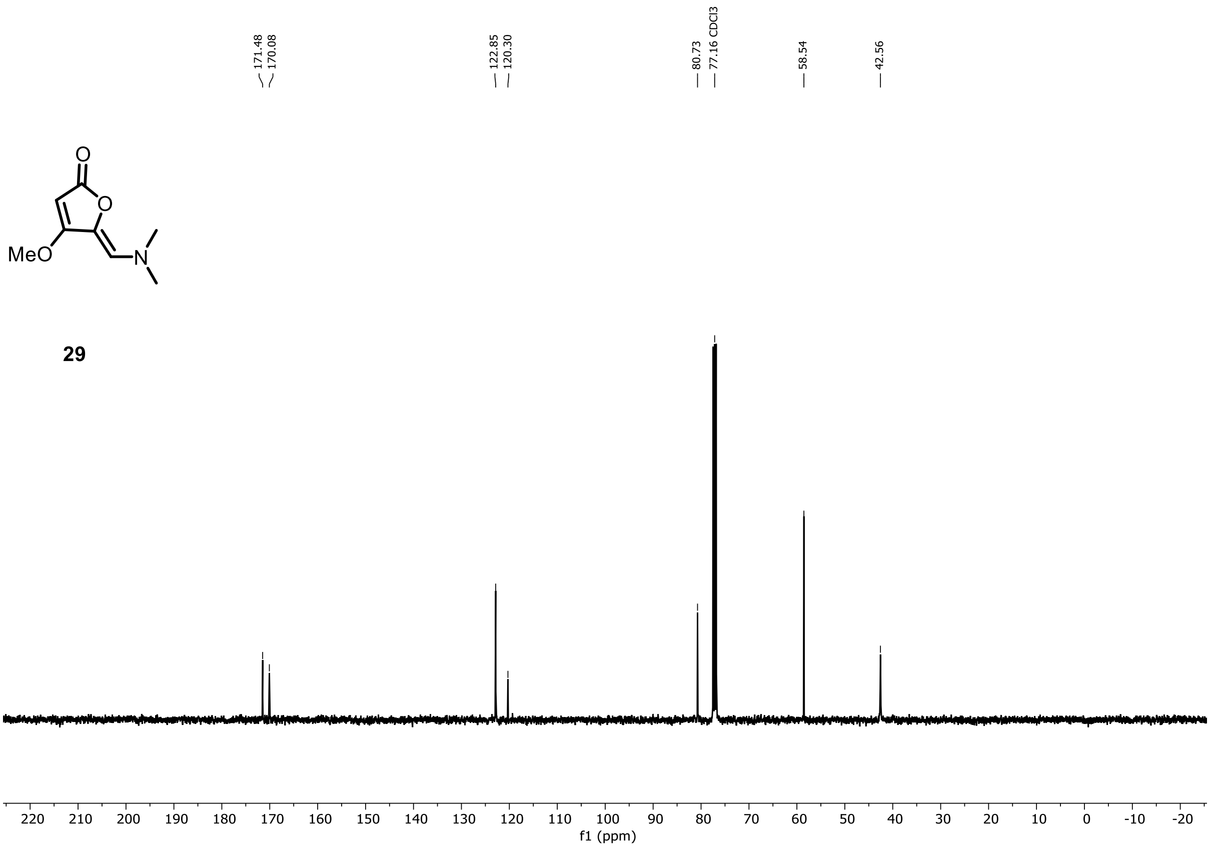
171.48
170.08
122.85
120.30
80.73
77.16 CDCl3
58.54
42.56
O
O
MeO
N
29
220 210 200 190 180 170 160 150 140 130 120 110 100 90 80 70 60 50 40 30 20 10 0 -10 -20
f1 (ppm)

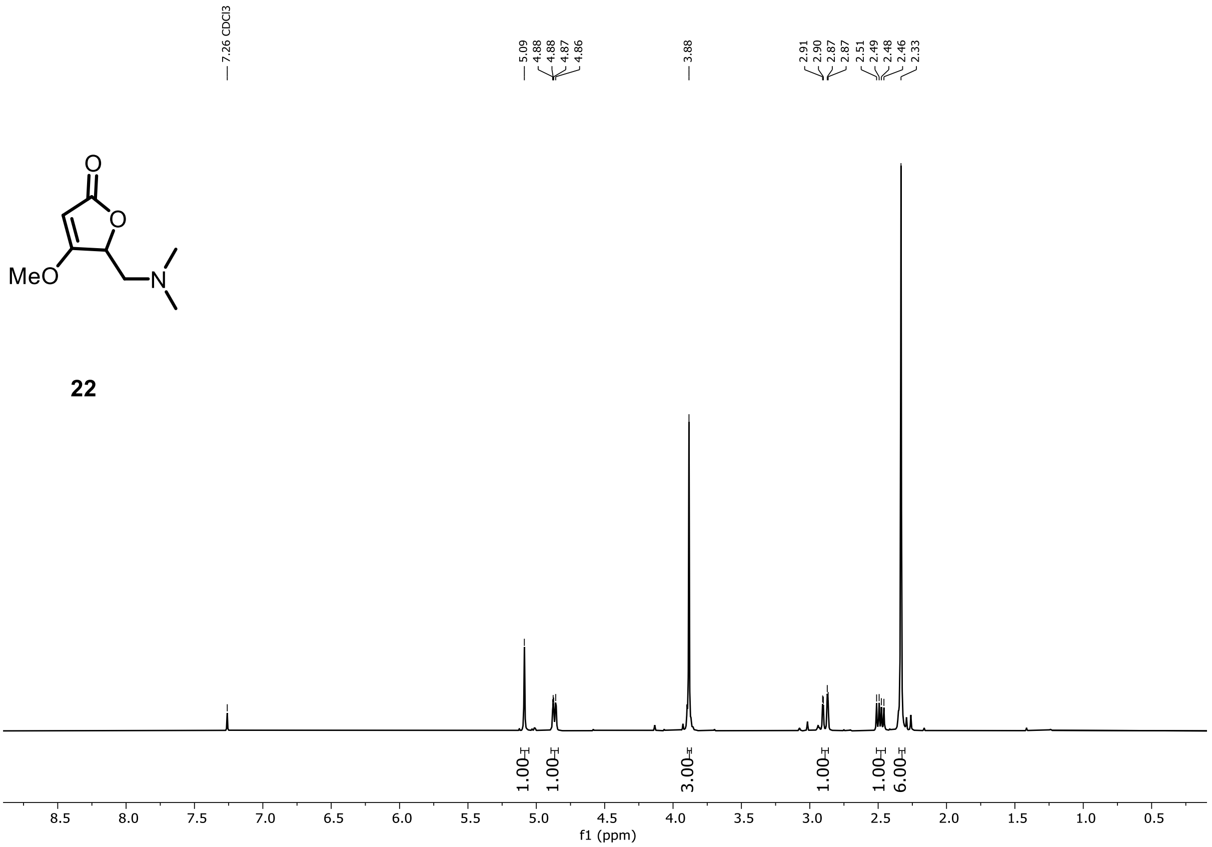
O
O
MeO
N
22
7.26 CDCl3
5.09
4.88
4.88
4.87
4.86
3.88
2.91
2.90
2.87
2.87
2.51
2.49
2.48
2.46
2.33
1.00
1.00
3.00
1.00
1.00
6.00
8.5 8.0 7.5 7.0 6.5 6.0 5.5 5.0 4.5 4.0 3.5 3.0 2.5 2.0 1.5 1.0 0.5
f1 (ppm)

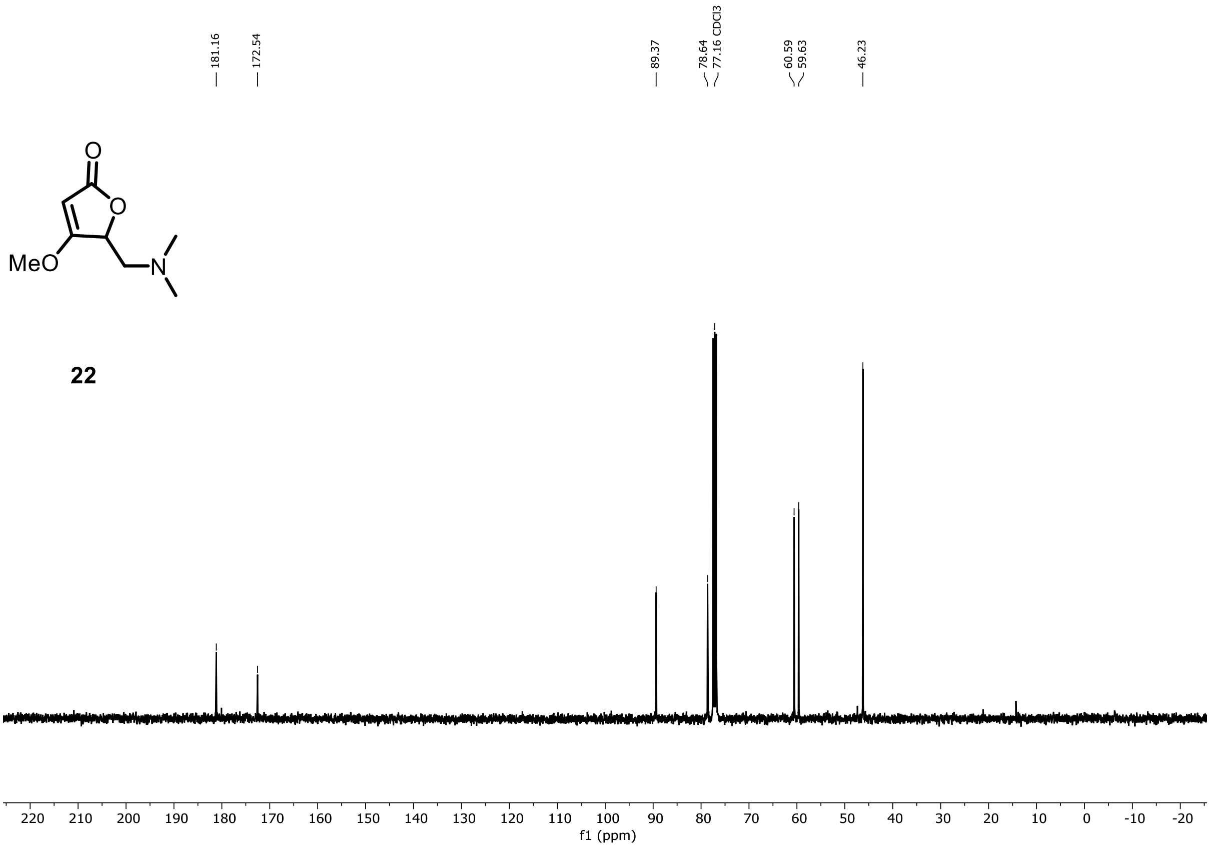
O
O
MeO
N
22
181.16
172.54
89.37
78.64
77.16 CDCl3
60.59
59.63
46.23
220 210 200 190 180 170 160 150 140 130 120 110 100 90 80 70 60 50 40 30 20 10 0 -10 -20
f1 (ppm)

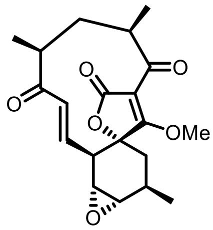

30

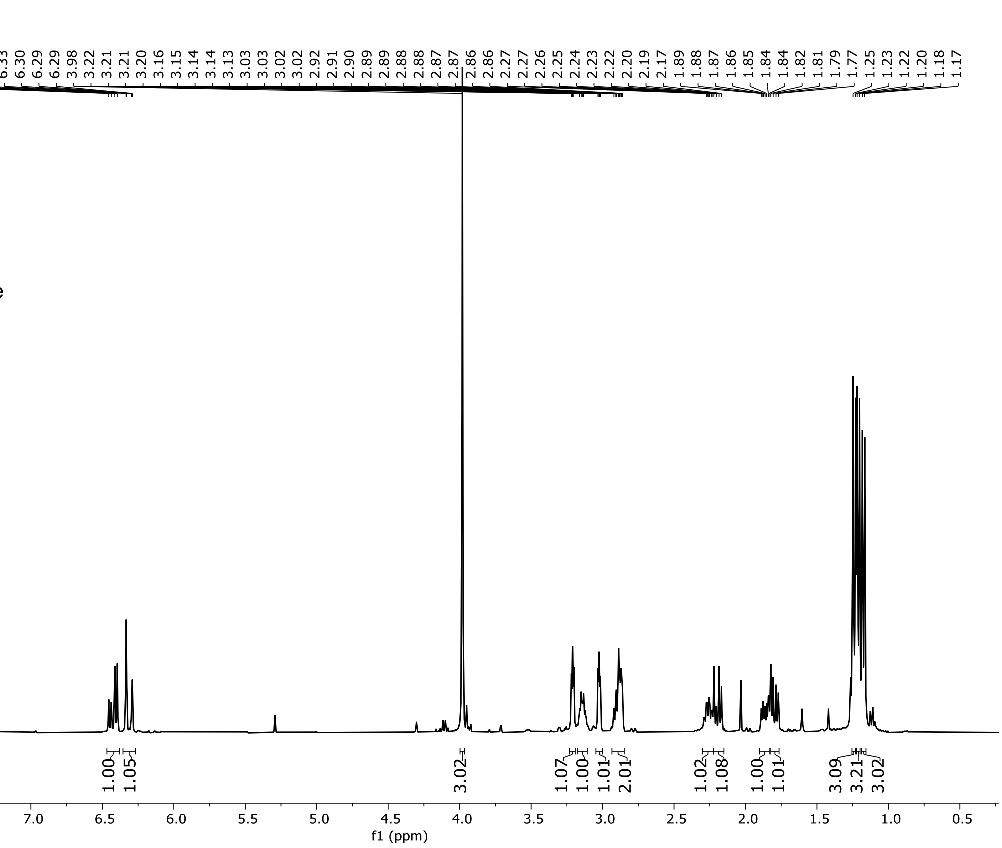
7.26 CDCl3
6.45
6.44
6.41
6.40
6.33
6.33
6.30
6.29
6.29
3.98
3.22
3.21
3.21
3.20
3.16
3.15
3.14
3.14
3.13
3.03
3.03
3.02
3.02
2.92
2.91
2.90
2.89
2.89
2.88
2.88
2.87
2.87
2.86
2.86
2.27
2.27
2.26
2.25
2.24
2.23
2.22
2.22
2.20
2.19
2.17
1.89
1.88
1.87
1.86
1.85
1.84
1.84
1.82
1.81
1.79
1.77
1.25
1.23
1.22
1.20
1.18
1.17
1.00
1.05
3.02
1.07
1.00
1.01
2.01
1.02
1.08
1.00
1.01
3.09
3.21
3.02
8.5
8.0
7.5
7.0
6.5
6.0
5.5
5.0
4.5
4.0
3.5
3.0
2.5
2.0
1.5
1.0
0.5
f1 (ppm)

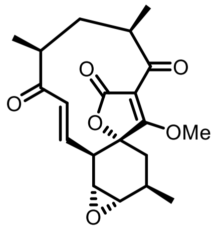

30

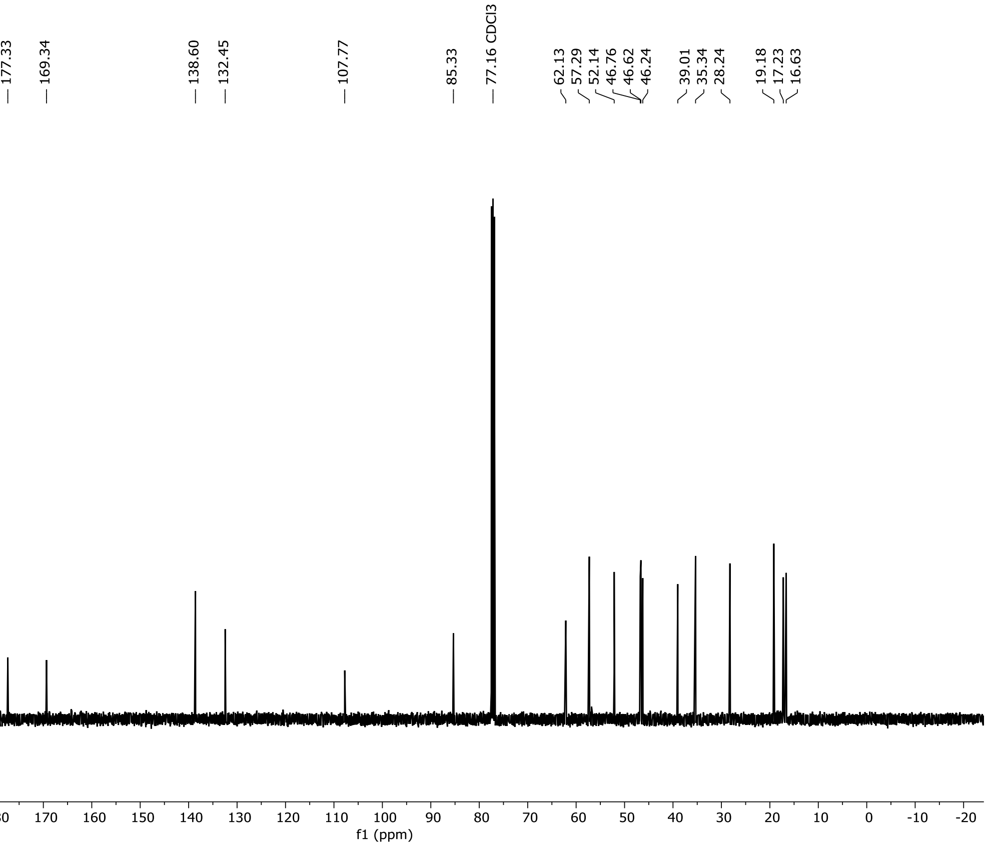
203.82
200.16
177.33
169.34
138.60
132.45
107.77
85.33
77.16 CDCl3
62.13
57.29
52.14
46.76
46.62
46.24
39.01
35.34
28.24
19.18
17.23
16.63
220
210
200
190
180
170
160
150
140
130
120
110
100
90
80
70
60
50
40
30
20
10
0
-10
-20
f1 (ppm)

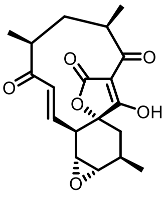
O
O
O
OH
O

4

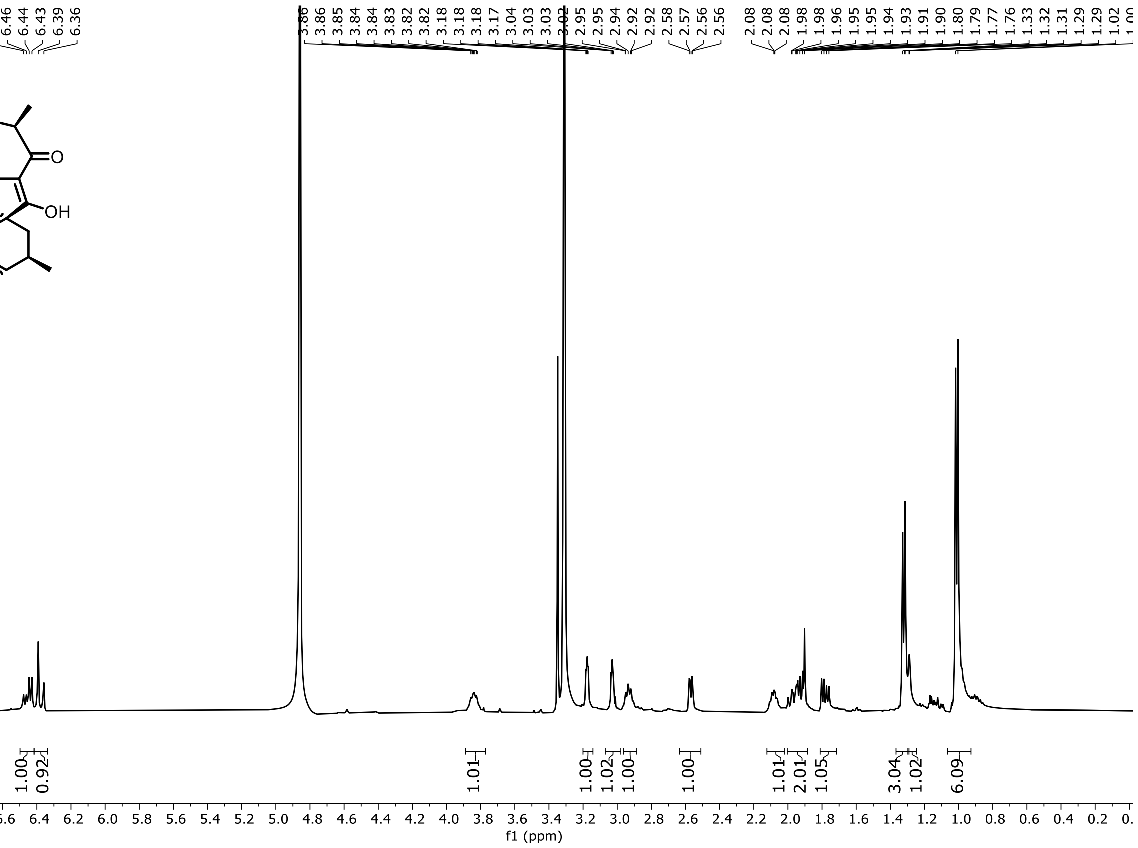
6.48
6.46
6.44
6.43
6.39
6.36
3.86
3.85
3.84
3.84
3.83
3.82
3.82
3.18
3.18
3.18
3.17
3.04
3.03
3.03
3.02
2.95
2.95
2.94
2.92
2.92
2.92
2.58
2.57
2.56
2.56
2.08
2.08
2.08
1.98
1.98
1.96
1.95
1.95
1.94
1.93
1.91
1.90
1.80
1.79
1.77
1.76
1.33
1.32
1.31
1.29
1.29
1.02
1.00
0.92
1.01
1.00
1.02
1.00
1.00
1.01
2.01
1.05
3.04
1.02
6.09
7.0 6.8 6.6 6.4 6.2 6.0 5.8 5.6 5.4 5.2 5.0 4.8 4.6 4.4 4.2 4.0 3.8 3.6 3.4 3.2 3.0 2.8 2.6 2.4 2.2 2.0 1.8 1.6 1.4 1.2 1.0 0.8 0.6 0.4 0.2
f1 (ppm)

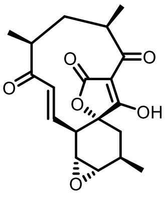
O
O
O
OH
O

4

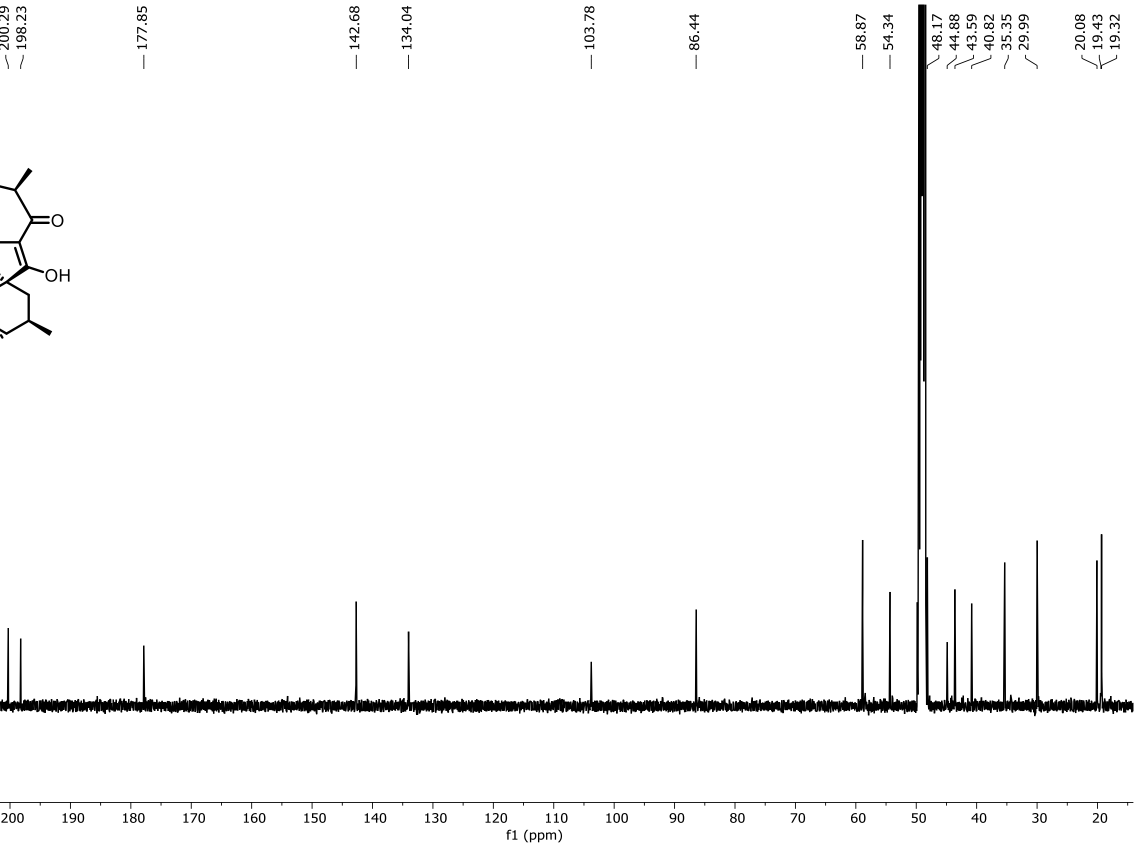
207.68
200.29
198.23
177.85
142.68
134.04
103.78
86.44
58.87
54.34
48.17
44.88
43.59
40.82
35.35
29.99
20.08
19.43
19.32
210 200 190 180 170 160 150 140 130 120 110 100 90 80 70 60 50 40 30 20
f1 (ppm)
